# Matched Analogue Analysis of Structural Responses Before and After Covalent Bond Formation in Wild-Type and A775_G776insYVMA HER2

**DOI:** 10.3390/ijms27209057

**Published:** 2026-10-13

**Authors:** Keke Fan, Yanan Xu, Qiwei Ye, Mingliang Zeng, Hongwang Xiao, Shanyan Mo, Sha Hu, Liming Hu

**Affiliations:** 1 Beijing Key Laboratory of Environmental and Viral Oncology, College of Chemistry and Life Science, Beijing University of Technology, Beijing 100124, China; 2 Beijing Key Laboratory for Green Catalysis and Separation, Beijing University of Technology, Beijing 100124, China; 3 Beijing Academy of Artificial Intelligence (BAAI), Beijing 100080, China

**Keywords:** HER2, A775_G776insYVMA, irreversible inhibitors, matched analogue pairs, reaction-compatible geometry, molecular dynamics, OpenComplex2, covalent adduct

## Abstract

Endpoint structure–activity relationships (SARs) for irreversible inhibitors can integrate reversible binding, covalent chemistry, conformational behavior, and cellular factors, making it difficult to assign a potency change to a single molecular event. We reanalyzed a previously reported series of irreversible inhibitors of wild-type (WT) HER2 and HER2 A775_G776insYVMA (HER2^YVMA^) as six matched analogue pairs. Three complementary analyses were kept physically distinct. Pre-reaction molecular dynamics (MD) trajectories were used to quantify the fraction of frames satisfying a predefined near-attack-conformation (NAC)-like distance–angle criterion; continuous structural descriptors calculated from OpenComplex2 (OC2)-generated covalently linked HER2–inhibitor structures were used to characterize structural differences after covalent linkage; and covalent-adduct MD trajectories were used to characterize post-bond-formation dynamics. The Michael addition step itself was neither simulated nor measured. Cyclization of compound **3** to compound **9c** increased the NAC-like frame fraction in HER2^WT^ from 0.0217% to 0.358% but decreased it in HER2^YVMA^ from 0.745% to 0.333%, with the overall direction preserved across all six sensitivity definitions. In HER2^YVMA^, the modification distinguishing compounds **4a** and **5a** increased the NAC-like fraction from 2.620% to 6.244% despite a loss of cellular potency. The analogous tail modification increased this fraction in the **8c**/**10e** series, whereas the HER2^YVMA^ **9c**/**11f** response was starting conformation sensitive. The distribution of OC2-generated covalently linked structures across the predefined structural coordinate and the final-adduct MD readouts likewise varied with HER2 context and scaffold, although some differential effects were sensitive to the coordinate reference. These analyses complement experimental SARs but do not provide a measure of reaction kinetics or thermodynamic populations.

## 1. Introduction

Structure–activity relationships (SARs) guide lead optimization by relating defined chemical changes to measured biological activity [1,2,3]. For irreversible inhibitors, however, an endpoint such as IC_50_ can reflect reversible recognition, conformational behavior, covalent chemistry, and cellular exposure [4,5,6]. The endpoint alone therefore does not identify which of these processes accounts for an observed SAR change [7,8]. Here, we asked whether structural analyses on either side of covalent bond formation could provide additional context for matched medicinal chemistry changes.

A simplified kinetic scheme for irreversible inhibition is *E* + *I* ⇌ *EI* → *E* − *I* [7,8,9,10]. Endpoint IC_50_ does not separately resolve formation of the reversible complex, the chemical step that forms the covalent bond, or the behavior of the final adduct [7,8,9,10]. We therefore kept three structural questions separate: geometric accessibility before bond formation, structural differences among already covalently linked complexes, and dynamics of the final covalent adduct. The NAC-like analysis was used only to describe pre-reaction geometry [11,12], whereas covalent-adduct MD readouts were used only to characterize post-bond-formation dynamics.

The three structural analyses were designed to interrogate different physical stages of irreversible inhibition rather than to serve as interchangeable computational methods. Pre-reaction MD was used to quantify reaction-compatible geometric accessibility before bond formation. Structural samples of covalently linked HER2–inhibitor complexes generated using OpenComplex2 (OC2, https://opencomplex.baai.ac.cn) were used to examine structural redistribution after covalent linkage, and covalent-adduct MD was used to characterize final-adduct dynamics. Accordingly, the analyses were used to test whether the structural response to a defined chemical perturbation was preserved across physical stages rather than to construct a single continuous mechanistic pathway [13]. Fractions assigned to discrete regions of the OC2 analysis coordinate were used only as operational summaries of the generated structures and were not interpreted as equilibrium populations or thermodynamic preferences [13].

HER2^WT^ and HER2 A775_G776insYVMA (HER2^YVMA^) [14,15,16] provide a defined system in which to ask whether the consequence of the same chemical change depends on HER2 context [17,18,19,20]. Our 2024 *Journal of Medicinal Chemistry* (*JMC*) study reported the structure-based optimization of an irreversible HER2 inhibitor series that included compounds **3** and **4a**, the cyclized 8/9 series, and the subsequent 10/11 series [21]. The study also reported HER2^WT^ biochemical potency, antiproliferative activity in Ba/F3 cells expressing HER2^YVMA^, activity in NCI-N87 and BT474 cells, pharmacokinetics (PK), and xenograft efficacy [21]. Within this chemical lineage, compound **4a** replaces the C5 methyl group of compound **3** with a phenyl substituent, whereas compounds **8c** and **9c** introduce pharmacophore-core cyclization. These relationships define analogue pairs in which the structural consequences of individual medicinal chemistry changes can be compared.

We analyzed six primary matched analogue pairs [2,3], rather than ranking the individual compounds, consistent with matched molecular pair analysis [1,22,23]. The analysis addressed three questions: whether the same modification produces different structural changes in HER2^WT^ and HER2^YVMA^; whether a corresponding tail modification behaves similarly in two cyclized scaffolds; and whether responses observed before bond formation are preserved in analyses of covalently linked complexes and final adducts. Experimental potency values were retained as independent reference endpoints and were not combined with the structural measurements. This design avoids treating readouts with different physical meanings as interchangeable.

## 2. Results

### 2.1. Published SAR Provides Six Matched Analogue Comparisons

All experimental measurements considered here were taken from the previously published *JMC* dataset, including HER2^WT^ biochemical kinase IC_50_ values, Ba/F3 HER2^YVMA^ antiproliferative IC_50_ values, activity in NCI-N87 and BT474 cells, PK, and xenograft data [21]. The original optimization progressed from C5 pocket filling and pharmacophore-core cyclization to modification of the tail adjacent to the Michael acceptor. Because HER2^WT^ potency and HER2^YVMA^ potency were measured in different assay formats, ΔpIC_50_ values were calculated only within each assay. The PK and xenograft results were considered separately as translational context.

Seven compounds from the original *JMC* series [21] were selected to represent the major medicinal chemistry transformations across the optimization sequence and define six primary matched pairs. Replacing the C5 methyl group of compound **3** with a phenyl group gives compound **4a** and tests a pocket-directed modification. Compounds **8c** and **9c** are two cyclization-derived analogues of compound **3**. Compound **5a** incorporates a tail modification adjacent to the Michael acceptor on the flexible quinazoline scaffold of compound **4a**. The corresponding class of tail modification is present in compounds **10e** and **11f** relative to compounds **8c** and **9c**, respectively, allowing the effect of this change to be compared in two cyclized scaffolds. Selection was based on the predefined medicinal chemistry transformations in the published lineage rather than on the OC2- or MD-derived structural response results reported here.

Replacing the C5 methyl group of compound **3** with the phenyl substituent in compound **4a** improved potency in both assays (HER2^WT^ ΔpIC_50_, +0.683; HER2^YVMA^ ΔpIC_50_, +0.666). The cyclized analogues **8c** and **9c** were also more potent than compound **3** in both assays, although the within-assay changes were larger in the WT biochemical assay. Because the assay formats differ, we did not compare the magnitudes of these changes quantitatively between HER2^WT^ and HER2^YVMA^. The tail modification in compound **5a** reduced potency relative to compound **4a** in both assays. In contrast, compounds **8c** and **10e** showed opposite potency changes in the two assays: the WT enzyme IC_50_ increased from 1.55 to 2.96 nM (ΔpIC_50_ = −0.281), whereas the HER2^YVMA^ Ba/F3 IC_50_ decreased from 10.80 to 1.49 nM (ΔpIC_50_ = +0.860). Compound **11f** was more potent than compound **9c** in both assays, with the larger within-assay change observed in the HER2^YVMA^ cellular assay. These published measurements provide the experimental reference for the structural comparisons below [21].

**Figure 1:**
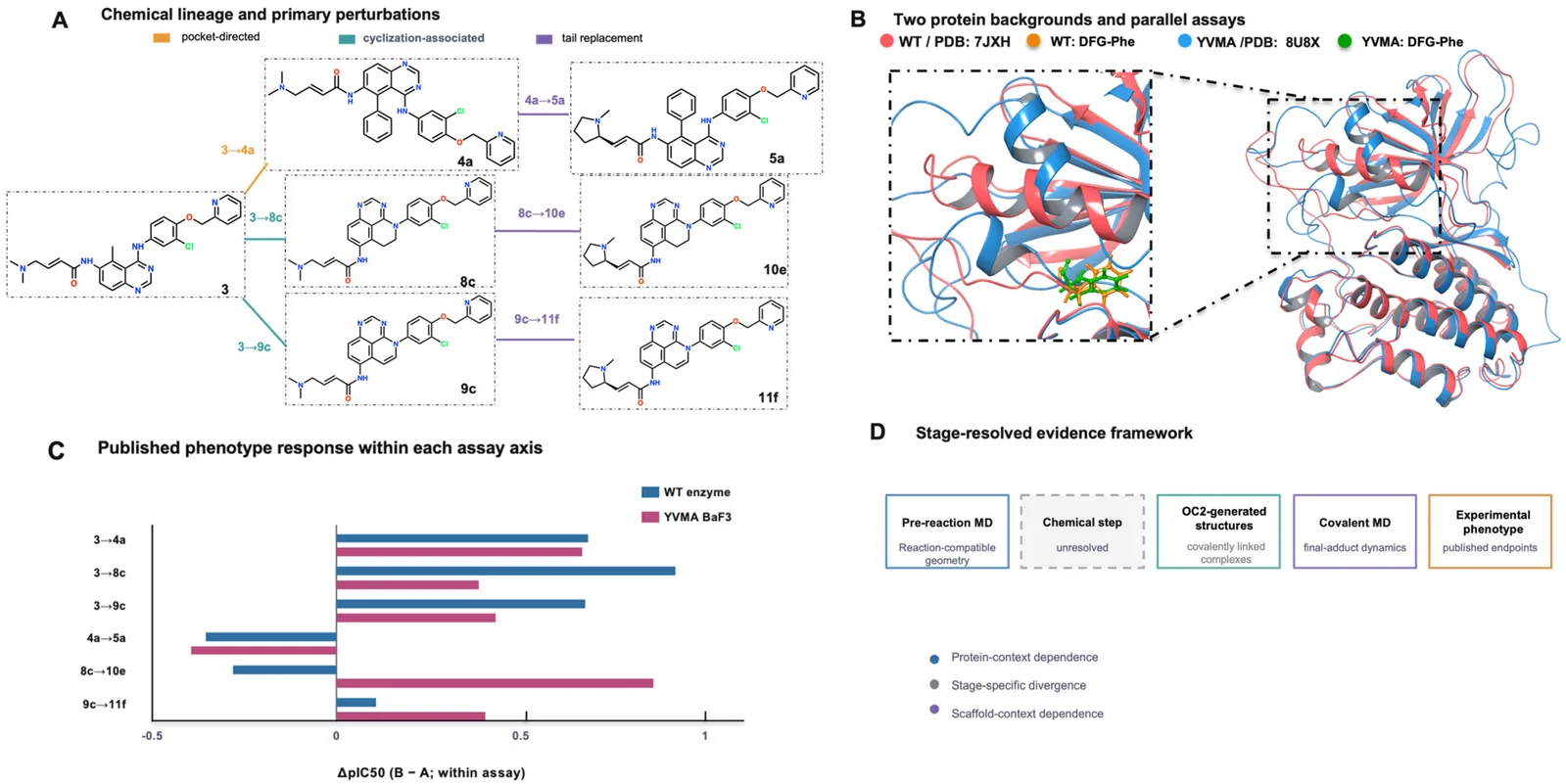
Transformation-centricperturbation–responseframeworkfor irreversible HER2 inhibition. (**A**) Chemical lineage and six primary matched analogue pairs, including replacement of the C5 methyl group in **3** with a phenyl group to give **4a**, cyclization-associated modifications of **3** leading to **8c** and **9c**, and tail modifications of **4a**, **8c**, and **9c** to afford **5a**, **10e**, and **11f**, respectively. (**B**) Structural backgrounds used for comparison: HER2^WT^ (PDB ID: 7JXH) and HER2^YVMA^ (PDB ID: 8U8X). (**C**) Published phenotype responses of the six analogue transformations in HER2^WT^ biochemical and HER2^YVMA^ Ba/F3 cellular assays, shown as within-assay ΔpIC_50_ values (Table S17). (**D**) Stage-resolved structural analysis workflow, including pre-reaction MD-derived reaction-compatible geometry, OC2-generated covalently linked structures, covalent-adduct MD, and published experimental endpoints. Panel C was newly plotted from the numerical potency values reported in Ref. [21].

For each matched pair, compound A denotes the initial analogue and compound B the modified analogue. Within the predefined analysis coordinate, Δ*P*_i_(*WT*) = *P*_i_(*WT*, *B*) − *P*_i_(*WT*, *A*) and Δ*P*_i_(*YVMA*) = *P*_i_(*YVMA*, *B*) − *P*_i_(*YVMA*, *A*). We then defined *Γ*_i_ = Δ*P*_i_(*YVMA*) − Δ*P*_i_(*WT*), which describes how the change in the assigned fraction differs between the two HER2 contexts. Unless otherwise specified, Γ refers to the full-data estimate calculated using all OC2-generated structures, whereas the equal-*N* estimate of Γ denotes the same quantity recalculated after equalizing the number of structures across conditions and was used only as a sample-size sensitivity analysis. For S1–S5 assignment fractions, ΔP and Γ are reported in percentage points (pp); for continuous structural descriptors, the analogous differential response, Γ*_j_* = Δ*D_j_*(YVMA) − Δ*D_j_*(WT), retains the unit of the corresponding descriptor (Å). For the experimental endpoints, ΔpIC_50_ values were calculated only within each assay, preserving the distinction between the HER2^WT^ biochemical measurement and the HER2^YVMA^ cellular measurement (Figure 1 and Table 1).

### 2.2. Matched Analogue Comparisons Identify Context-Dependent Differences in Pre-Reaction Geometry

We defined reaction-compatible geometry using a near-attack-conformation (NAC)-like distance–angle criterion [11,12]. The primary definition required a Cys Sγ–electrophilic Cβ distance ≤ 4.0 Å and an Sγ–Cβ–Cα angle of 90–125°. Sensitivity was assessed with six definitions combining distance thresholds of 3.5, 4.0, and 4.5 Å with angle ranges of 90–125° and 95–120°. The resulting fraction is used only as a measure of geometric accessibility before bond formation. Each compound–protein condition was represented by three independent MD trajectories. The Sγ–Cβ–Cα angular term was used as an operational oblique-approach proxy for nucleophilic approach to the electrophilic β-carbon; it was not interpreted as a transition state geometry, a validated reaction coordinate, reaction probability, or covalent reactivity. The six definitions were used only to test sensitivity to this operational geometric criterion.

#### 2.2.1. C5 Phenyl Substitution Increases the Fraction of Reaction-Compatible Geometries in Both HER2 Contexts

Replacing the C5 methyl group of compound **3** with the phenyl substituent in compound **4a** increased the fraction of MD frames satisfying the primary NAC-like criterion from 0.0217% to 7.486% in HER2^WT^ and from 0.745% to 2.620% in HER2^YVMA^. Local contact analysis also showed more raw protein–ligand contacts for compound **4a** in both HER2 contexts, but this increase was not retained after normalization by ligand heavy-atom number. Thus, the C5 substitution was associated with greater pre-reaction geometric accessibility, whereas the raw contact increase was size-dependent. Structural differences among covalently linked complexes are considered separately below and are not assumed to arise from the same interaction.

#### 2.2.2. Cyclization Alters Pre-Reaction Geometry Differently in HER2^WT^ and HER2^YVMA^

Compounds **3** and **9c** provided the clearest HER2-context-dependent pre-reaction response. Relative to compound **3**, compound **9c** increased the fraction of reaction-compatible frames in HER2^WT^ from 0.0217% to 0.358% but decreased it in HER2^YVMA^ from 0.745% to 0.333%. At the pooled-frame level, the direction of change was retained under all six sensitivity definitions in both contexts (6/6 increases in HER2^WT^ and 6/6 decreases in HER2^YVMA^), although replicate-level directions were not uniform. These pooled results indicate that the same cyclization-associated modification changes the predefined pre-reaction geometry differently in HER2^WT^ and HER2^YVMA^.

The comparison of compounds **3** and **8c** showed a related but more cutoff-sensitive pattern. In HER2^WT^, the fraction increased from 0.0217% to 0.890%, with the same direction under all six definitions. In HER2^YVMA^, the fraction decreased from 0.745% to 0.635% under the primary criterion; decreases were observed at four of six cutoffs and increases at two, both reversals occurring at the broader 4.5 Å distance threshold. The HER2^WT^ direction was therefore retained across all six criterion definitions, whereas the HER2^YVMA^ result depended more strongly on the geometric definition.

#### 2.2.3. Pre-Reaction Geometry and Cellular Potency Diverge for Compounds **4a** and **5a**

In HER2^YVMA^, the tail modification distinguishing compounds **4a** and **5a** increased the fraction of reaction-compatible frames from 2.620% to 6.244%. The increase was observed under all six sensitivity definitions and in all three independent trajectories. The published Ba/F3 HER2^YVMA^ IC_50_, however, increased from 5.66 to 14.06 nM, indicating lower endpoint potency. For this matched pair, greater geometric accessibility by the predefined NAC-like criterion did not coincide with greater cellular potency.

#### 2.2.4. The Corresponding Tail Modification Gives Different Pre-Reaction Responses in the Two Cyclized Series

For compounds **8c** and **10e**, the fraction of reaction-compatible frames increased from 0.890% to 1.567% in HER2^WT^ and from 0.635% to 3.792% in HER2^YVMA^. The increase in HER2^YVMA^ was retained under all six sensitivity definitions. In this cyclized series, the tail modification was therefore associated with both greater pre-reaction geometric accessibility in HER2^YVMA^ and improved published cellular potency.

The corresponding tail modification behaved differently in the series containing compounds **9c** and **11f**. The reaction-compatible fraction increased in HER2^WT^ from 0.358% to 2.320% under all six definitions but decreased in HER2^YVMA^ for the original **11f** starting conformation from 0.333% to 0.0017% under all six definitions, even though the published HER2^YVMA^ Ba/F3 IC_50_ improved from 9.69 to 3.82 nM. For HER2^YVMA^ with compound **11f**, the original starting conformation yielded only one NAC-positive frame among 59,994 frames from three trajectories. This single event occurred in the trajectory with seed 3456, indicating extremely sparse sampling of the predefined geometry under this condition. A second chemically valid HER2^YVMA^–**11f** starting conformation yielded 5.70%, 7.25%, and 5.40% NAC-like frames across three additional 200 ns trajectories (6.12% pooled), demonstrating strong starting conformation sensitivity under the present protocol (Table S5C).

**Figure 2:**
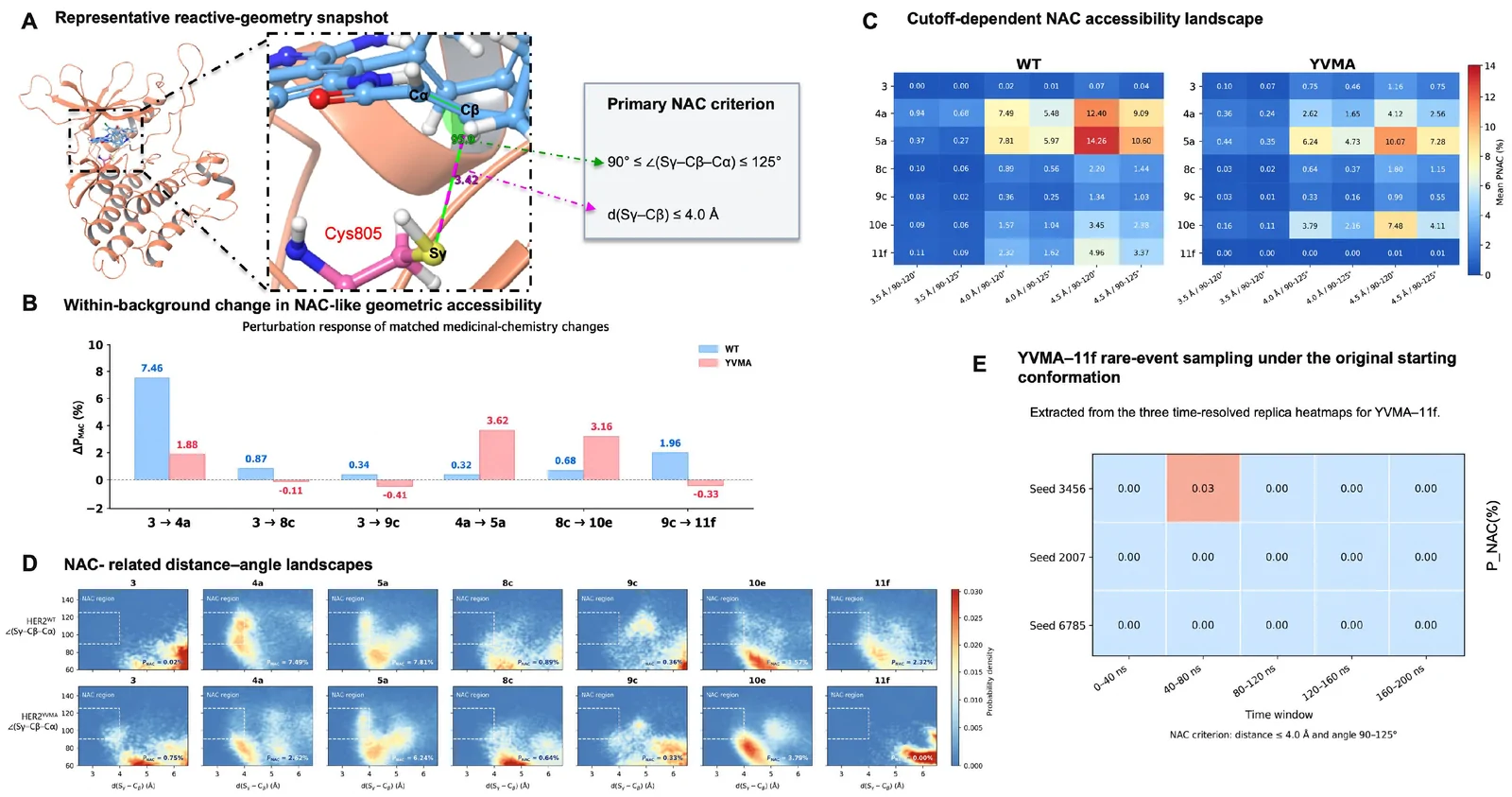
Pre-reaction reaction-compatible geometry analysis (Tables S2–S5). (**A**) Representative reaction-compatible geometry snapshot and the primary NAC-like criterion based on the Cys Sγ–electrophile Cβ distance and Sγ–Cβ–Cα angle. (**B**) Overall pre-reaction accessibility across six matched analogue pairs, showing the fraction of MD frames satisfying the primary NAC-like criterion in HER2^WT^ and HER2^YVMA^. (**C**) Cutoff-dependent sensitivity analysis of NAC accessibility using alternative distance and angle definitions. (**D**) Distance–angle density landscapes of reaction-compatible geometry for HER2^WT^ and HER2^YVMA^ conditions. (**E**) Replica-level analysis of rare reaction-compatible geometry sampling for HER2^YVMA^–**11f**. The Sγ–Cβ–Cα angle is an operational oblique-approach descriptor and is not intended to represent a transition state geometry or validated reaction coordinate.

For both tail modification pairs, joint distributions of the Cys–electrophile distance and attack angle were calculated from all trajectory frames (59,994 frames per condition). Because reaction-compatible geometry was exceptionally rare for HER2^YVMA^ with compound **11f** in the original starting conformation, the analysis also included the raw event count, episode duration, block convergence, and autocorrelation to distinguish an isolated event from sustained sampling within a trajectory (Figure 2 and Figures S1–S3).

### 2.3. Structural Descriptors from OC2-Generated Covalently Linked Structures Show Context-Dependent Perturbation Responses

We next asked whether the structural response to a matched chemical perturbation observed before bond formation was preserved after covalent linkage. To compare structures in which the covalent bond was already present, we defined a common analysis coordinate from 918 apo HER2^WT^ structures derived from the PDB ID: 7JXH starting structure [24]. Four continuous descriptors were used: DFG_RMSD_ref_A, alphaC_RMSD_ref_A, Lys51_Glu68_min_distance_A, and Ploop_RMSD_ref_A [25,26,27]. HER2^YVMA^ structures and OC2-generated structures from covalently linked HER2–inhibitor complexes were projected into this fixed coordinate and were not used to define its boundaries. External checks against experimental HER2 structures 3RCD/7PCD (WT) and 8VB5 (YVMA) supported conformational coverage beyond the starting templates (Figure S16; Table S18).

Continuous structural descriptors were used as the primary readouts of structural perturbation response across the OC2-generated structures, whereas discrete clustering served only as a coarse operational summary of their distribution within the predefined structural coordinate [28,29,30]. Cluster number QC was performed across *k* = 2–10. A *k* = 2 partition provided a coarse grouping of the generated structures (silhouette ≈ 0.414; ARI ≈ 0.988), whereas *k* = 5/S1–S5 provided a higher-resolution operational grouping (silhouette ≈ 0.243; ARI ≈ 0.612). The *k* = 5 solution was used only to summarize assignment within this analysis coordinate; it was not treated as a thermodynamic state model. Higher-*k* sensitivity analyses at *k* = 6–8 mainly subdivided or refined the existing S1–S5 groups rather than revealing an additional qualitatively distinct structural group (Table S8).

Interpretation therefore relied primarily on the continuous DFG RMSD, αC RMSD, P-loop RMSD, Lys–Glu geometry, and related descriptors. The *k* = 2 grouping provided coarse support, and S1–S5 values were reported as the fractions of OC2-generated structures assigned to the predefined *k* = 5 regions. We use S1–S5 only as operational labels and do not infer thermodynamic stabilization or destabilization from changes in these assignment fractions.

For compounds **3** and **4a**, raw local protein–ligand contact readouts increased in both HER2^WT^ and HER2^YVMA^, whereas heavy-atom-normalized contact density decreased in both backgrounds. In the *k* = 5 representation, the fraction assigned to S1 increased by 1.38 percentage points (pp) in HER2^WT^ and decreased by 8.82 pp in HER2^YVMA^, giving Γ_S1_ = −10.20 pp. The direction was retained in the equal-*N* sensitivity analysis. Thus, the same matched modification was accompanied by different changes in the distribution of OC2-generated structures across the predefined coordinate in the two HER2 contexts. The raw contact change and the reduction in S1 assignment in HER2^YVMA^ co-occurred, but these data do not establish a causal relationship between them.

HER2-context-dependent descriptor changes were also observed for the cyclization-derived analogues. For compounds **3** and **8c**, the S1 assignment fraction changed by −0.15 pp in HER2^WT^ and −9.26 pp in HER2^YVMA^ (Γ = −9.11 pp; equal-*N* Γ = −8.60 pp). For compounds **3** and **9c**, the corresponding changes were −4.37 pp and −8.48 pp in HER2^WT^ and HER2^YVMA^, respectively (Γ = −4.11 pp; equal-*N* Γ = −3.60 pp). The continuous Lys–Glu descriptor showed the same general context dependence: the change for compounds **3** and **8c** was −0.296 Å in HER2^WT^ and +0.485 Å in HER2^YVMA^, whereas the change for compounds **3** and **9c** was +0.300 Å in HER2^WT^ and +0.654 Å in HER2^YVMA^. The continuous descriptors provide the primary evidence for different structural responses in the two HER2 contexts, whereas the discrete grouping is retained only as a supporting representation of structural redistribution (Tables S6B and S7).

For compounds **4a** and **5a**, the S1 assignment fraction changed by −4.25 pp in HER2^WT^ and approximately +0.52 pp in HER2^YVMA^, giving Γ_S1_ = +4.77 pp. In HER2^YVMA^, descriptor changes across the OC2-generated covalently linked structures occurred alongside increased pre-reaction geometric accessibility but lower endpoint potency, indicating that structural responses at different physical stages need not vary in parallel with one another or with the final activity measurement. These S1 values describe assignment within the operational k = 5 coordinate and do not imply thermodynamic stabilization or destabilization.

For the two tail modification pairs, the differential changes in S1–S5 assignment were smaller and more sensitive to equal-*N* analysis. For compounds **8c** and **10e**, the full-data Γ values for S1–S5 were (−1.13, −0.05, −0.45, +0.14, +1.49) pp; for compounds **9c** and **11f**, they were (−3.79, +1.67, +0.13, +2.07, −0.08) pp, with some assignments changing direction after equal-*N* analysis. Repeated resampling of the continuous descriptors gave median Γ values of −0.016 (DFG), −0.048 (αC), −0.257 (Lys–Glu), +0.005 (P-loop), and −0.007 Å (C5-shell) for compounds **8c** and **10e**, with direction-retention values of 0.784, 0.876, 0.960, 0.544, and 0.760, respectively (Table S9). For compounds **9c** and **11f**, the corresponding median Γ values were +0.057, +0.026, +0.545, +0.005, and −0.023 Å, with direction-retention values of 0.996, 0.683, 1.000, 0.548, and 0.962. These results place greater interpretive weight on the continuous descriptor changes than on any single *k* = 5 assignment fraction. Near-zero Γ values were not interpreted as meaningful directional changes.

All 18,138 OC2-generated structures across the 14 covalently linked HER2–inhibitor conditions were included in the analysis. The reported S1–S5 fractions are the proportions of structures assigned to the corresponding regions of the predefined coordinate; ΔP and Γ describe matched-condition changes in these assignment fractions.

**Figure 3:**
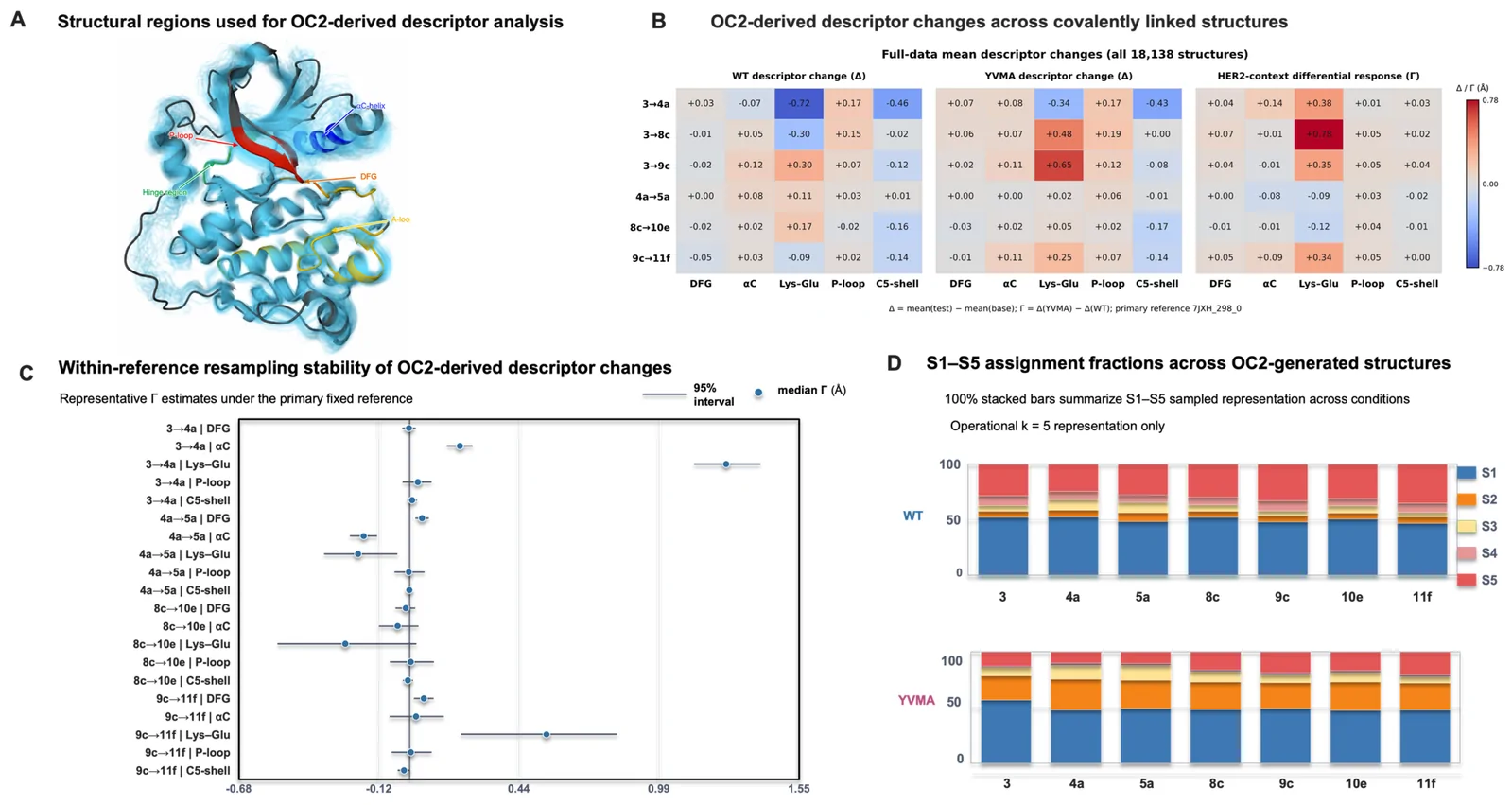
OC2-derived continuous descriptor changes in the full dataset of covalently linked HER2–inhibitor complexes. (**A**) Structural regions used for descriptor analysis. (**B**) Descriptor changes for six matched analogue transformations in HER2^WT^ and HER2^YVMA^, with context-dependent response (Γ). (**C**) Within-reference resampling consistency of representative Γ estimates, shown as medians and 95% intervals. (**D**) S1–S5 assignment fractions across OC2-generated structures in HER2^WT^ and HER2^YVMA^; S1–S5 denote an operational *k* = 5 grouping. Panel (**C**) assesses within-set resampling stability under the primary coordinate reference and should not be interpreted as coordinate reference robustness; alternative reference sensitivity is reported separately in Table S10.

We assessed sensitivity to sample size, cluster representation, and coordinate reference. Repeated equal-*N* and resampling analyses provided effect-size and direction-retention estimates. Descriptor-space out-of-distribution (OOD) analysis yielded OOD_95_ = 1.972 and OOD_99_ = 2.834, with an OOD_99_ fraction below 5% in all 14 conditions. Recalculation using the alternative apo HER2^WT^ reference 7JXH_261_2.pdb reversed the direction of some Γ values, indicating that a subset of differential descriptor changes depended on the coordinate reference. Resampling stability and coordinate reference sensitivity were treated as distinct analyses; retention of a direction under repeated resampling does not imply robustness to the choice of coordinate reference (Table S10 and Figures S4–S7) (Figure 3).

### 2.4. Covalent-Adduct MD Characterizes Post-Bond-Formation Dynamics

Covalent-adduct MD simulations were initiated from the final adduct, with the Cys–inhibitor bond already formed, to characterize dynamics after bond formation. The cross-condition analysis included 14 compound–protein combinations, each represented by three independent replicas, for a total of 42 retained trajectories.

For compounds **3** and **4a**, the covalent-adduct trajectories showed more local protein–ligand contacts with compound **4a** in both HER2^WT^ and HER2^YVMA^, together with changes in ligand and protein RMSD and regional structural descriptors. These final-adduct measurements were evaluated separately from the pre-reaction geometry and from descriptors calculated from the OC2-generated covalently linked structures, so similarities across analyses were not assigned a common physical meaning.

Final-adduct accommodation of compound **3** relative to compounds **8c** and **9c** was evaluated using ligand and protein RMSD, DFG-, αC-, and P-loop dynamics, Lys–Glu geometry, C5/back-pocket shell distance, and protein–ligand contacts. The main-text interpretation emphasizes local accommodation and kinase regulatory geometry; global protein RMSD and related measurements are treated as complementary descriptors.

For compounds **4a** and **5a**, the retained trajectories showed similar geometries to the established covalent bond. This observation supports consistent covalent mapping across the two systems but does not account for their different endpoint potencies. It further illustrates the distinction between pre-reaction geometric accessibility and the structural behavior of the final adduct.

Topology validation verified the expected Sγ–Cβ single-bond connectivity in all 14 covalent HER2^WT^ and HER2^YVMA^ systems. The electrophile state, adduct connectivity, atom types, and partial charges were also checked for consistency before the final-adduct analyses (Table S14).

All final-adduct comparisons used the same 0–200 ns analysis window, with independent trajectories as the statistical units. Identical seed labels in different molecular systems were treated only as trajectory indices rather than paired replicas, and statistical summaries were calculated from trajectory-level readouts.

The covalent-adduct trajectories for compounds **8c** and **10e** and for compounds **9c** and **11f** were analyzed separately to determine whether the corresponding tail modification produced similar post-bond-formation responses in the two cyclized scaffolds.

**Figure 4:**
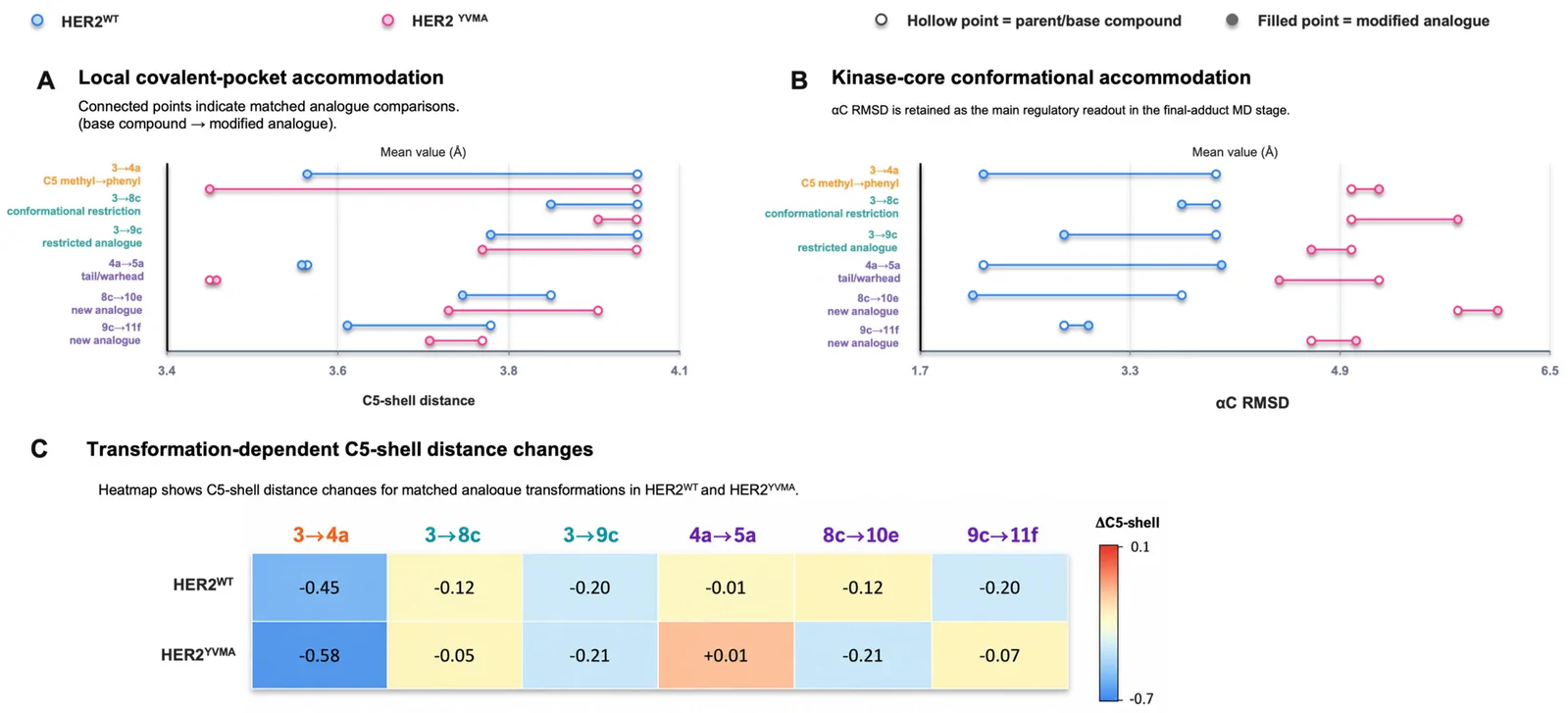
Covalent-adduct MD reveals post-bond-formation structural accommodation (Tables S11 and S12). (**A**) C5-shell distance across six matched analogue transformations in HER2^WT^ and HER2^YVMA^. (**B**) Kinase-core conformational adaptation assessed by αC RMSD. (**C**) Transformation-dependent changes in C5-shell distance (ΔC5-shell) in HER2^WT^ and HER2^YVMA^. Hollow and filled symbols indicate parent/base and modified analogues, respectively.

Chemical state validation was performed separately for the pre-reaction and covalently linked systems. All 14 pre-reaction systems retained the electrophile-adjacent carbon double bond; SG and HG atoms were present for HER2^WT^ Cys805 and HER2^YVMA^ Cys809; and the ligand formal charge was +1 in every case, consistent with the protonation and formal-charge states assigned by Epik. Covalent systems were validated for Sγ–Cβ single-bond connectivity, adduct connectivity, and parameter assignment (Figure 4 and Figures S8–S15).

### 2.5. Matched Modifications Give Different Responses Across HER2 Contexts, Scaffolds, and Structural Analyses

We compared the results according to the structural question addressed by each analysis: pre-reaction MD quantified NAC-like geometry before bond formation [11,12]; continuous structural descriptors calculated from OC2-generated covalently linked structures characterized structural differences after covalent linkage [13]; covalent-adduct MD characterized dynamics of the final adduct [31]; and the experimental assays provided independent endpoint measurements. Keeping these readouts separate permits matched chemical changes to be compared across analyses without implying that the measurements have the same physical meaning.

A consistent correspondence between any single structural measurement and potency was not detected across the six matched pairs. Instead, the response to a chemical perturbation depended on HER2 context, scaffold, and physical stage. These results argue against a universal one-to-one mapping between any individual structural readout and the final pharmacological phenotype.

**Figure 5:**
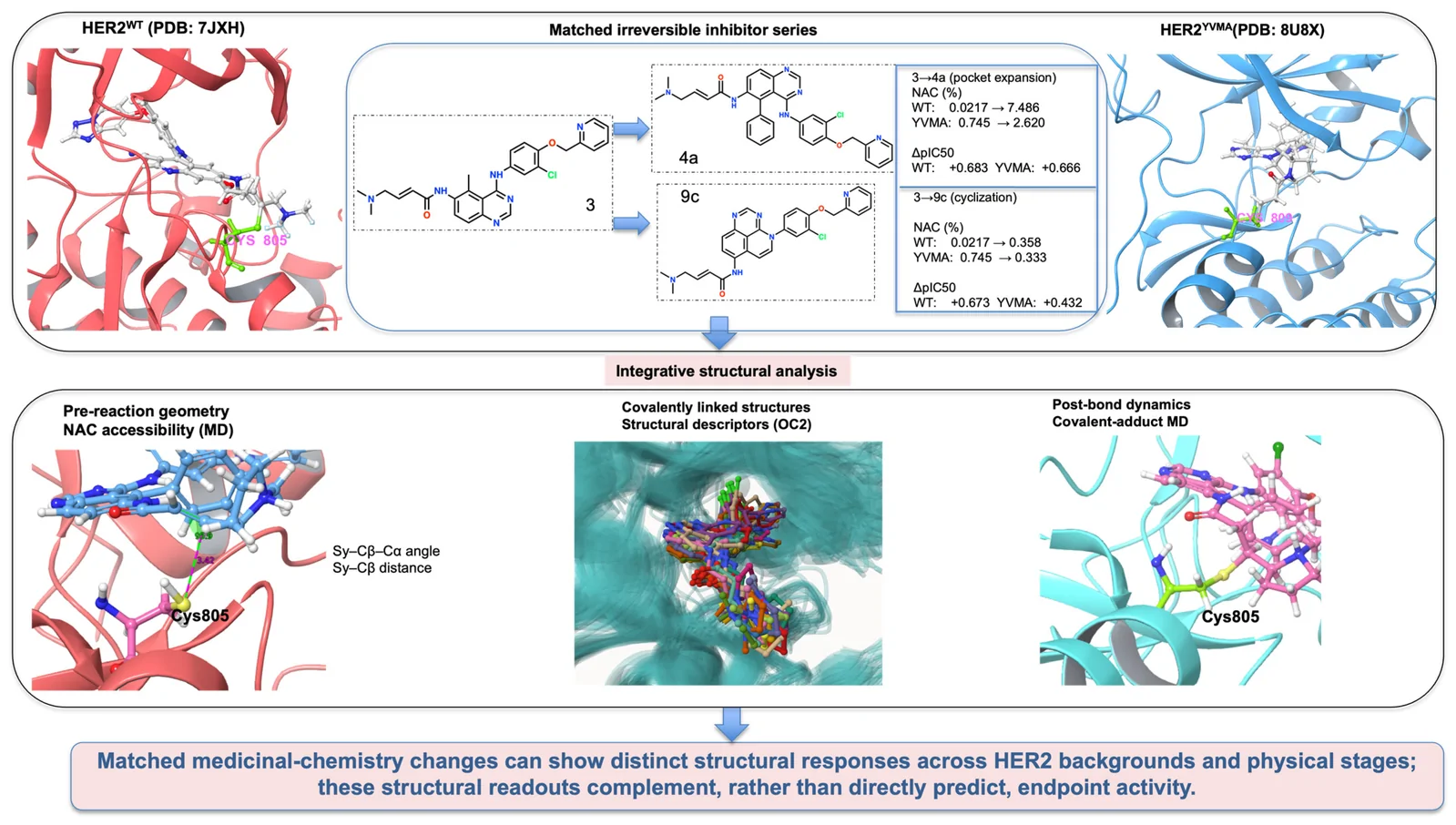
Matched analogue analysis reveals HER2-context-dependent structural responses in irreversible HER2 inhibition. Representative matched analogue comparisons in HER2^WT^ and HER2^YVMA^ are summarized across pre-reaction MD-derived reaction-compatible geometry, OC2-generated covalently linked structures, covalent-adduct MD, and published experimental endpoints. Experimental potency is shown as an independent within-assay reference; no quantitative cross-assay mutation effect is inferred, and the structural readouts are not presented as direct predictors of endpoint activity.

Accordingly, pre-reaction geometry, structural descriptors calculated from OC2-generated covalently linked structures, covalent-adduct dynamics, and endpoint activity are reported as distinct measurements rather than combined into a weighted or composite mechanistic score (Table 2 and Figure 5).

## 3. Discussion

### 3.1. The Same Chemical Modification Can Have Different Structural Consequences in HER2^WT^ and HER2^YVMA^

Among the matched pairs, compounds **3** and **9c** provided the clearest example of dependence on HER2 context. The cyclization-associated modification increased the overall reaction-compatible fraction in HER2^WT^ but decreased it in HER2^YVMA^. Compounds **3** and **8c** showed a related pattern, although the HER2^YVMA^ response was more sensitive to the cutoff definition. These comparisons indicate that a medicinal chemistry change can alter pre-reaction geometry differently in HER2^WT^ and HER2^YVMA^. This interpretation is consistent with broader evidence that kinase mutations can reshape conformational ensembles [17,29,32,33] and alter the availability of inhibitor-compatible states [34,35,36].

Endpoint activity did not mirror this pre-reaction response. Compound **9c** was more potent than compound **3** in both assays even though the reaction-compatible fraction changed in opposite directions in HER2^WT^ and HER2^YVMA^. A context-dependent structural response therefore need not produce an analogous reversal in endpoint potency. Because the HER2^YVMA^ result for compound **8c** was cutoff sensitive, that comparison provides supporting rather than primary evidence for this interpretation.

### 3.2. Pre-Reaction Geometry Does Not Track Endpoint Potency Monotonically

The comparison of compounds **4a** and **5a** illustrates why the NAC-like metric should not be treated as a surrogate for potency or covalent reaction rate [11,12]. In HER2^YVMA^, compound **5a** sampled the predefined reaction-compatible geometry more frequently than compound **4a**, yet its published Ba/F3 potency was lower. The NAC-like analysis therefore reports only geometric accessibility within the specified distance–angle criterion [11,12].

Endpoint potency for an irreversible inhibitor can depend on reversible binding, warhead reactivity [37,38,39,40], the chemical reaction step [7,8,9,10], cellular exposure, and the behavior of the covalent adduct, among other factors [4,5,6,41]. The discordance between compounds **4a** and **5a** is therefore consistent with the expectation that a single pre-reaction geometric descriptor will not vary monotonically with the final activity measurement.

### 3.3. The Effect of a Matched Tail Modification Depends on Scaffold Context

The comparisons of compounds **8c** and **10e** and of compounds **9c** and **11f** show that the corresponding tail modification can have different structural consequences in different cyclized scaffolds.

From a medicinal chemistry perspective, this result argues against treating a substituent effect as an intrinsic property of the substituent alone [22,23,42]. The consequence of a chemical change depends on the surrounding scaffold and protein environment. A modification that is favorable in one series may therefore alter geometry or conformational sampling differently in another, even when the nominal chemical change is similar.

### 3.4. Implications for Interpreting SAR of Irreversible Inhibitors

The central implication is that the endpoint SAR can integrate molecular events occurring at different points in an irreversible inhibition process [4,5,6]. Pre-reaction MD trajectories quantify a predefined geometry before bond formation [11,12]; descriptors calculated from OC2-generated covalently linked structures provide a separate comparison after covalent linkage [13]; and covalent-adduct MD trajectories describe dynamics of the final adduct [31]. In addition, the coordinate used to analyze the OC2-generated structures was defined from apo HER2^WT^, so the S1–S5 assignment fractions are comparative summaries within that coordinate rather than thermodynamic populations.

These measurements have different physical meanings. The NAC-like criterion reports pre-reaction geometric accessibility within a specified distance–angle definition [11,12]. Continuous structural descriptors calculated from OC2-generated covalently linked structures characterize differences among these model-generated structures [13]. Trajectory-derived readouts from covalent-adduct MD characterize post-bond-formation dynamics [31]. IC_50_ integrates additional biochemical or cellular processes [7,8,9,10]. The HER2^WT^ biochemical IC_50_ and HER2^YVMA^ Ba/F3 IC_50_ were obtained in different assay formats and must likewise be interpreted separately.

The same distinction applies to translational endpoints. In the original *JMC* study, compound **10e** produced 110.7% tumor growth inhibition in a Ba/F3 HER2^YVMA^ xenograft model but had an oral bioavailability of 7.43%. Compound **11f** produced 17.8% tumor growth inhibition, and the original report attributed its weaker in vivo performance in part to poor solubility [21]. PK and xenograft outcomes depend on exposure, solubility, and other factors beyond kinase-site geometry; they therefore provide context rather than direct validation of any individual structural descriptor.

The experimental SAR remains the basis for medicinal chemistry decisions, and matched molecular pair analysis provides a formal way to relate defined structural changes to property changes [1,2,3]. The additional structural analyses used here separately characterize pre-reaction geometric accessibility, structural differences among OC2-generated covalently linked complexes, and final-adduct dynamics, while the experimental endpoint is interpreted independently as an integrated pharmacological measurement.

### 3.5. Limitations

Several factors define the scope of the present analysis. The Michael addition chemical step itself was not simulated, and the NAC-like criterion was used only as a geometric accessibility measure; it does not resolve cysteine activation, proton transfer, intrinsic electrophilicity, or the reaction energy barrier. OC2 structures provide model-generated representations of covalently linked complexes. External comparisons with experimental HER2 structures were used only as conformational coverage diagnostics, not as evidence of thermodynamic or equilibrium populations; independent repeat-inference variability was not evaluated separately from the within-ensemble resampling analyses, and some differential descriptor responses were sensitive to the coordinate reference. The HER2^YVMA^–**11f** NAC result was also sensitive to the starting conformation. In addition, WT and YVMA simulations were initiated from different experimental templates, and this study was limited to seven compounds with assay-specific activity endpoints. These considerations limit mechanistic attribution but do not preclude the use of the analyses as comparative structural readouts for the predefined matched transformations. The pre-reaction NAC-like results are therefore conditional on the modeled neutral-thiol microstate.

## 4. Materials and Methods

### 4.1. Experimental Data Sources and Potency Measurements

Compound structures, HER2^WT^ biochemical kinase potency, antiproliferative potency in Ba/F3 cells expressing HER2^YVMA^, NCI-N87 and BT474 data, PK, and in vivo efficacy were taken from the published 2024 *JMC* study [21]. Because the HER2^WT^ kinase assay and the HER2^YVMA^ Ba/F3 cellular assay use different formats, IC_50_ values were converted to pIC_50_ and ΔpIC_50_ was calculated only within each assay as pIC_50_(B) − pIC_50_(A). NCI-N87/BT474, PK, and xenograft data were used only as phenotypic and translational context (Table S17).

### 4.2. Definition of Matched Analogue Pairs

The six primary matched pairs were compounds **3** and **4a**, compounds **3** and **8c**, compounds **3** and **9c**, compounds **4a** and **5a**, compounds **8c** and **10e**, and compounds **9c** and **11f**. The last two pairs introduce the same class of tail modification in different cyclized scaffold contexts and were used to assess transferability across scaffolds. These compounds were selected to span the predefined medicinal chemistry transformations in the published lineage, rather than on the basis of the OC2- or MD-derived structural response results reported here.

### 4.3. HER2 Structures, Residue Numbering, and Atom Mapping

PDB ID: 7JXH was used for HER2^WT^, with Cys805 as the reactive residue [24]. PDB ID: 8U8X was used for HER2^YVMA^, in which the corresponding reactive residue is Cys809 [20]. For the OC2 sequence inputs used here, the WT and YVMA kinase-domain sequences differed by the four-residue YVMA insertion. In the experimental structures used for the Maestro-based workflows, 7JXH additionally contains a short N-terminal construct segment that is spatially remote from the binding-site region. Trajectory and geometry analyses followed the residue numbering of the respective structures. The ligand electrophilic Cβ and adjacent Cα atoms were mapped according to homologous ligand topology (Table S13 and Figure S19).

### 4.4. Pre-Reaction Molecular Dynamics Simulations

Pre-reaction MD simulations of HER2^WT^ and HER2^YVMA^ were performed without a covalent bond between the reactive cysteine and the inhibitor. Each compound–protein combination was represented by three independent trajectories with seed labels 2007, 3456, and 6785; identical seed labels across different molecular systems served only as indices for otherwise independent replicas. For HER2^YVMA^–**11f**, an additional set of three 200 ns trajectories was generated from a second chemically valid starting conformation to assess starting conformation sensitivity (Table S5C). Each 200 ns trajectory used for geometric analysis contained approximately 19,998 measurement frames, and frame-level measurements were summarized as trajectory-level fractions. Reaction-compatible geometry was defined using the NAC-like Cys Sγ–electrophilic Cβ distance and Sγ–Cβ–Cα attack angle [11,12]. The primary criterion was d ≤ 4.0 Å and an angle of 90–125°, with sensitivity analysis across distance thresholds of 3.5, 4.0, and 4.5 Å and angle ranges of 90–125° and 95–120°. HER2^WT^ Cys805 and HER2^YVMA^ Cys809 were modeled as neutral thiols, and system preparation in Maestro used an Epik pH range centered at 7.4 (±2.0); ligand protonation, tautomeric state, and formal charge were recorded. A common neutral-thiol microstate was used for Cys805/Cys809 across the pre-reaction systems so that matched comparisons probed geometric accessibility under a fixed modeled chemical state; cysteine deprotonation and proton transfer were not represented by this setup (Table S1).

Systems were constructed with the OPLS4 force field [43] and SPC water. Each system was placed in an orthorhombic periodic box with a 10 Å buffer in the a, b, and c directions. The Minimize Volume option in Desmond System Builder was applied independently to each system, allowing final box dimensions to vary. Systems were neutralized according to net charge and supplemented with 0.15 M NaCl. Production simulations were run in Schrödinger/Desmond 2025-4 [44] in the NPT ensemble at 300 K and an isotropic pressure of 1.01325 bar. Parameters included MTK ensemble control, thermostat τ = 1.0 ps, barostat τ = 2.0 ps, a 200,000 ps (200 ns) production time, [0.002, 0.002, 0.006] ps multiple-time-step integration, a 9.0 Å nonbonded cutoff, a 10 ps trajectory interval, a 1.2 ps energy-sequence interval, and a 240.06 ps checkpoint interval. Initial velocities were randomized, and replicas used custom seeds 2007, 3456, and 6785.

Before production, the standard Desmond NPT relaxation protocol was applied [44]. The sequence comprised an auto/setup stage; 100 ps of Brownian-dynamics NVT relaxation at 10 K ([0.001, 0.001, 0.003] ps time steps; positional-restraint force constant, 50.0 kcal mol^−1^ Å^−2^, on solute heavy atoms); 12 ps of NVT at 10 K (Langevin thermostat τ = 0.1 ps; velocity randomization interval, 1.0 ps); 12 ps of restrained NPT at 10 K (Langevin τ = 0.1 ps; barostat τ = 50 ps); 12 ps of restrained NPT at the target simulation temperature (thermostat τ = 0.1 ps; barostat τ = 50 ps); and 24 ps of unrestrained NPT relaxation (thermostat τ = 0.1 ps; barostat τ = 2.0 ps). This relaxation was followed by the 200 ns NPT production simulation (Figures S17 and S18).

### 4.5. Reaction-Compatible Geometry and NAC-like Analysis

Reaction-compatible geometry was defined using the NAC-like Cys Sγ–electrophilic Cβ distance and Sγ–Cβ–Cα attack angle [11,12]. The primary criterion was d ≤ 4.0 Å and an angle of 90–125°. Sensitivity analysis used six definitions combining distance thresholds of 3.5, 4.0, and 4.5 Å with angle ranges of 90–125° and 95–120°. Measurements were generated for every trajectory frame and summarized as trajectory-level fractions, with each independent trajectory treated as one replicate. For HER2^YVMA^ with compound 11f in the original starting conformation, rare-event analysis additionally included the raw positive-frame count, episode duration, distance and angle distributions, joint density, block convergence, and autocorrelation. The angular term was used as an operational oblique-approach proxy for nucleophilic approach to the electrophilic β-carbon; the 90–125° window was not treated as a transition state geometry or as a validated or quantitative reaction coordinate. The nearby distance and angle definitions were evaluated only as sensitivity tests of this operational criterion.

### 4.6. Generation of OC2 Structural Samples

OpenComplex2 (OC2) [13] was used to generate structural samples of HER2–inhibitor complexes in which the covalent linkage between the reactive cysteine and inhibitor was already present. The OC2 model used here was trained on publicly available structural data released before 1 January 2021; the HER2 reference structures 7JXH and 8U8X were released after this cutoff and were therefore not part of the training data. Covalently linked complexes were encoded through the molecular-graph input, with the ligand and reactive cysteine involved in the adduct represented at atomic resolution and the remaining protein represented at residue/group resolution; predictions were performed without MSA input. Across the 14 covalently linked HER2–inhibitor conditions, all 18,138 OC2-generated structures were included in the downstream analyses without post-generation quality-based filtering. For a fixed molecular input, each OC2 structure was generated as an independent draw from the same model-defined structural distribution, rather than by temporal propagation from a preceding structure. Thus, OC2 outputs constitute an unordered set of independent structural samples rather than a time-correlated trajectory, and their relative frequencies were not interpreted as thermodynamic or kinetic populations (Table S16).

### 4.7. Construction of the Analysis Coordinate for OC2-Generated Structures

A total of 918 apo HER2^WT^ structures generated by OC2 from the 7JXH starting condition were aligned using 212 common stable kinase-core Cα positions (analysis sequence indices 10–23, 33–58, 78–139, and 191–300). The descriptors DFG_RMSD_ref_A, alphaC_RMSD_ref_A, Lys51_Glu68_min_distance_A, and Ploop_RMSD_ref_A were then calculated using kinase conformation features established in prior structural classification work [25,26,27]. These four descriptors defined the clustering coordinate. The C5-shell distance was analyzed separately as an auxiliary continuous structural descriptor and was not included in the clustering model. StandardScaler was fit only to the apo HER2^WT^ structures. KMeans used n_clusters = 5, random_state = 42, and n_init = 30. The DFG/αC/P-loop RMSD descriptors used 7JXH_298_0.pdb as the fixed coordinate reference, selected as the apo structure closest to the mean aligned kinase-core conformation. HER2^YVMA^ structures and OC2-generated structures from covalently linked HER2–inhibitor systems were projected into this fixed coordinate without refitting the cluster boundaries.

### 4.8. Continuous Structural Descriptors and Operational Cluster Assignments

Cluster number analysis was performed for *k* = 2–10 using the same standardized four-descriptor representation and clustering protocol. Clustering stability was assessed using the adjusted Rand index (ARI). Higher-*k* sensitivity analyses at *k* = 6–8 compared cluster assignments and the median values of the four descriptors with the *k* = 5 representation to assess whether increasing *k* mainly subdivided the existing S1–S5 groups. Continuous structural descriptors were the primary readouts, and *k* = 5/S1–S5 was used for higher-resolution operational visualization. For each primary matched pair, Δ*P*_i_(*WT*), Δ*P*_i_(*YVMA*), and *Γ*_i_ = Δ*P*_i_(*YVMA*) − Δ*P*_i_(*WT*) were calculated within the same operational coordinate.

### 4.9. Robustness and Sensitivity Analyses of OC2-Derived Structural Descriptors

Robustness analyses of descriptors calculated from the OC2-generated structures included repeated equal-*N* analysis, repeated resampling/bootstrap, direction retention, alternative *k* values, out-of-distribution (OOD) analysis based on assignment distance, a C5-shell/hinge geometry-proxy subset, coordinate reference sensitivity, and finite sampling robustness. OOD analyses reported OOD_95_, OOD_99_, and per-structure assignment distances. Reference sensitivity was assessed by recalculating descriptors for the covalently linked complexes using the alternative apo HER2^WT^ reference 7JXH_261_2.pdb. Because some Γ values changed direction with the reference choice, these analyses were used to identify differential descriptor changes that depend on the coordinate reference. For compounds 10e and 11f, hinge proximity was evaluated using the homologous N37-to-Met N distance as an auxiliary OC2-derived structural descriptor (Tables S13 and S15).

### 4.10. Covalent-Adduct Molecular Dynamics Simulations

Covalent-adduct MD simulations were initiated from the final adduct with an established Cys–inhibitor bond [31]. Cross-condition comparisons included 14 compound–protein combinations, each represented by three independent replicas, for a total of 42 retained trajectories analyzed over a common 0–200 ns window. Readouts included ligand heavy-atom RMSD, protein Cα RMSD, C5/back-pocket shell distance, DFG-, αC-, and P-loop dynamics, Lys–Glu geometry, protein–ligand contacts, and local contact network descriptors. Protein–ligand contacts followed the Schrödinger 2025-4 Simulation Interaction Diagram definitions: H bonds, distance cutoff ≤ 2.5 Å with donor/acceptor angular criteria ≥120°/≥90°; π-cation contacts, distance cutoff ≤ 4.5 Å; nonspecific hydrophobic contacts, distance cutoff ≤ 3.6 Å; ionic contacts, distance cutoff ≤ 3.7 Å; halogen bonds, distance cutoff ≤ 3.5 Å; and water-bridge H bonds, distance cutoff ≤ 2.8 Å with donor/acceptor angular criteria ≥110°/≥90°. π–π interactions were classified using the corresponding Schrödinger face-to-face or face-to-edge aromatic-stacking criteria.

### 4.11. Chemical State and Topology Validation

Chemical state and topology validation of the covalent systems included Sγ–Cβ single-bond connectivity, the electrophile state, adduct connectivity, atom types, partial charges, and bond, angle, and torsion parameter assignments [31]. All 14 compound–protein systems passed these consistency checks, supporting the connectivity and parameterization used for the subsequent covalent-adduct analyses. The covalent linkage and associated bonded topology were prepared in the Schrödinger/Maestro–Desmond workflow and inspected in Maestro before production MD.

### 4.12. Statistical Analysis and Interpretation

For analyses of OC2-generated structures, uncertainty was assessed using effect sizes from the full dataset together with resampling and alternative representation analyses. For pre-reaction MD-derived reaction-compatible geometry, independent trajectories were the statistical units. Each condition contained n = 3 trajectories; frame-level measurements were summarized at the trajectory level and were not treated as independent replicates. Reported summaries included individual replica values, condition means, SD or range, directional consistency, six-definition sensitivity, and rare-event analysis. Readouts from different parts of the inhibition process were analyzed separately and were not combined into a single mechanistic score.

## 5. Conclusions

We reanalyzed a previously reported irreversible HER2 inhibitor series as six matched analogue pairs and compared structural readouts before and after covalent bond formation in HER2^WT^ and HER2^YVMA^. Cyclization of compound **3** to compound **9c** showed opposite pooled changes in the fraction of reaction-compatible MD frames in the two HER2 contexts. In HER2^YVMA^, the tail modification distinguishing compounds **4a** and **5a** increased pre-reaction geometric accessibility despite lower endpoint potency, whereas the corresponding tail modifications in the two cyclized series produced different pre-reaction responses.

The data indicate that the structural consequence of a medicinal chemistry modification can depend on HER2 context, scaffold, and the structural question being examined. Their value is to identify matched chemical changes for which pre-reaction geometric readouts, structural descriptors calculated from OC2-generated covalently linked structures, final-adduct MD readouts, and experimental potency provide different information.

## Figures and Tables

**Table 1:** Six primary matched analogue pairs and their published potency responses.

Matched Pair	Chemical Modification or Scaffold Context	HER2^WT^ Kinase IC_50_, Initial and Modified (nM)	HER2^WT^ ΔpIC_50_	Ba/F3 HER2^YVMA^ IC_50_, Initial and Modified (nM)	HER2^YVMA^ ΔpIC_50_
**3** vs. **4a**	C5 methyl replaced by phenyl; pocket-directed modification	12.87 to 2.67	+0.683	26.22 to 5.66	+0.666
**3** vs. **8c**	Pharmacophore-core cyclization	12.87 to 1.55	+0.919	26.22 to 10.80	+0.385
**3** vs. **9c**	Alternative pharmacophore-core cyclization	12.87 to 2.73	+0.673	26.22 to 9.69	+0.432
**4a** vs. **5a**	Tail modification on the flexible quinazoline scaffold	2.67 to 6.03	−0.354	5.66 to 14.06	−0.395
**8c** vs. **10e**	Corresponding tail modification on the **8c** cyclized scaffold	1.55 to 2.96	−0.281	10.80 to 1.49	+0.860
**9c** vs. **11f**	Corresponding tail modification on the **9c** cyclized scaffold	2.73 to 2.13	+0.108	9.69 to 3.82	+0.404

Note: For each pair, the first value corresponds to the initial analogue and the second to the modified analogue. Experimental IC_50_ values were taken from the published HER2 irreversible-inhibitor study [21]; Δ*pIC*_50_ = *pIC*_50_(*B*) − *pIC*_50_(*A*); positive values indicate increased potency within the corresponding assay. HER2^WT^ values were obtained in a biochemical kinase assay, whereas HER2^YVMA^ values were obtained in a Ba/F3 antiproliferative assay. Because the assay formats differ, the two sets of values were interpreted separately and were not combined into a cross-assay mutation ΔΔpIC_50_.

**Table 2:** Comparison of the six matched analogue pairs across structural readouts and published potency (Tables S2–S12 and S17).

Matched Pair	NAC-like Fraction: HER2^WT^	NAC-like Fraction: HER2^YVMA^	OC2 Descriptor Response	Covalent-Adduct MD	Published ΔpIC_50_ (HER2^WT^/HER2^YVMA^)
**3→4a**	0.0217% → 7.486%	0.745% → 2.620%	Background-dependent	Context-dependent	+0.683/+0.666
**3→8c**	0.0217% → 0.890%	0.745% → 0.635%	Background-dependent	Context-dependent	+0.919/+0.385
**3→9c**	0.0217% → 0.358%	0.745% → 0.333%	Background-dependent	Broadly concordant	+0.673/+0.432
**4a→5a**	7.486% → 7.807%	2.620% → 6.244%	Background-dependent; mixed across readouts	Mixed	−0.354/−0.395
**8c→10e**	0.890% → 1.567%	0.635% → 3.792%	Scaffold-dependent; some reference-sensitive effects	Mixed	−0.281/+0.860
**9c→11f**	0.358% → 2.320%	Starting conformation sensitive	Scaffold-dependent; some reference-sensitive effects	Broadly concordant	+0.108/+0.404

Note: Pre-reaction MD-derived geometry uses the primary NAC-like criterion d(Sγ–Cβ) ≤ 4.0 Å and ∠Sγ–Cβ–Cα = 90–125°. Direction counts in parentheses refer to the six-definition sensitivity analysis. The OC2 column summarizes changes in continuous structural descriptors calculated from OC2-generated covalently linked structures. Detailed descriptor values, resampling and reference sensitivity analyses, and the *k* = 5 operational grouping are provided in the Supporting Information. The covalent-adduct MD column summarizes post-bond-formation structural responses across the matched analogue comparisons. Each condition is based on three independent replicas, and all comparisons use the same 0–200 ns window. The published potency column reports within-assay ΔpIC_50_ values in HER2^WT^/HER2^YVMA^ order. Detailed numerical values are provided in the Supporting Information.

## Data Availability

The Supporting Information contains the principal processed data underlying the reported analyses, including trajectory-level NAC-like summaries, OC2-derived descriptor and assignment summaries, robustness analyses, atom-mapping information, covalent-adduct MD summary statistics, and external structure coverage results. The OC2 structural samples, trajectory-level summary datasets, and analysis scripts are available from the corresponding author upon reasonable request. No public repository accession is currently associated with these materials.

## Supplementary Materials

The following supporting information can be downloaded at https://www.mdpi.com/article/10.3390/ijms27209057/s1.

## Abbreviations

ARI, adjusted Rand index; DFG, Asp–Phe–Gly motif; HER2, human epidermal growth factor receptor 2; MD, molecular dynamics; NAC-like criterion, near-attack-conformation-like distance–angle criterion; NPT, constant number of particles, pressure, and temperature ensemble; NVT, constant number of particles, volume, and temperature ensemble; OC2, OpenComplex2; OOD, out-of-distribution; OOD95/OOD99, 95th/99th percentile OOD thresholds; PDB, Protein Data Bank; PK, pharmacokinetics; pp, percentage points; RMSD, root-mean-square deviation; S1–S5, operational k = 5 structural regions; SAR, structure–activity relationship; WT, wild type; YVMA, A775_G776insYVMA insertion variant of HER2; αC, αC helix.
