# Supplementary material for: Matched Analogue Analysis of Structural Responses Before and After Covalent Bond Formation in Wild-Type and A775_G776insYVMA HER2
Source: Int J Mol Sci. 2026 Oct 13;27(20):9057. doi: 10.3390/ijms27209057 (PMC13705266; doi:10.3390/ijms27209057)
Supplement: Supplementary file 1 [file ijms-27-09057-s001.zip › ijms-4607533-supplementary.pdf]

SUPPORTING INFORMATION

Matched Analogue Analysis of Structural Responses Before and After Covalent Bond Formation in Wild-Type and A775_G776insYVMA HER2

Keke Fan [1], Yanan Xu [1,2], Qiwei Ye [3], Mingliang Zeng [3], Hongwang Xiao [3], Shanyan Mo [1], Sha Hu [1,3,*] and Liming Hu [1,*]

1 Beijing Key Laboratory of Environmental and Viral Oncology, College of Chemistry and Life Science, Beijing University of Technology, Beijing 100124, China
2 Beijing Key Laboratory for Green Catalysis and Separation, Beijing University of Technology, Beijing 100124, China
3 Beijing Academy of Artificial Intelligence (BAAI), Beijing 100080, China
* Correspondence: husha637@bjut.edu.cn (S.H.); huliming@bjut.edu.cn (L.H.)

S1. Supplementary Methods

S1.1 Experimental perturbation definition

The analysis was organized around six predefined medicinal-chemistry perturbations, represented by the matched analogue pairs 3 and 4a, 3 and 8c, 3 and 9c, 4a and 5a, 8c and 10e, and 9c and 11f. These perturbations were treated as the primary analysis units. $HER2^{WT}$ biochemical potency and Ba/F3-HER2YVMA cellular potency were retained as parallel phenotype axes and were not combined into a formal cross-assay mutation term.

S1.2 Reaction-compatible geometry analysis

Pre-reaction molecular dynamics trajectories were analyzed using predefined distance-angle criteria to quantify reaction-compatible geometry. The resulting fraction was interpreted as pre-reactive geometric accessibility. Individual trajectories rather than individual frames were treated as statistical units. Each 200 ns trajectory was additionally divided into five consecutive 40 ns windows for visualization of temporal heterogeneity, and P_NAC was calculated within each window; these windowed values were not treated as independent replicates. The Sγ–Cβ–Cα angular term was treated as an operational oblique-approach proxy for

nucleophilic approach to the electrophilic β-carbon, not as a transition-state geometry, validated reaction coordinate, reaction probability, or covalent reactivity. The six cutoff combinations were used only as criterion-sensitivity tests. Pre-reaction comparisons used the same modeled neutral-thiol cysteine microstate across systems, so the reported geometric-accessibility fractions are conditional on that microstate.

S1.3 OC2 generated-structure analysis

OC2-generated covalently linked HER2–inhibitor structures were analyzed using continuous structural descriptors as the primary readouts, including DFG RMSD, αC RMSD, Lys51–Glu68 minimum distance, and P-loop RMSD. Additional k=5 assignments were used only as a higher-resolution operational representation of sampled structures and were not interpreted as equilibrium populations or thermodynamic states. Higher-k sensitivity was additionally assessed at k = 6–8 by comparing cluster sizes and median values of the four descriptors with the k = 5 representation.

S1.4 External experimental-structure coverage analysis

External conformational coverage of the apo OC-generated structural sets was assessed against independent HER2 structures 3RCD and 7PCD (WT) and 8VB5 (YVMA). For each comparison, the starting template (7JXH or 8U8X) and all OC-generated apo structures were aligned using the same reference-specific Cα correspondence: a corrected 232-Cα core for 3RCD, a 238-Cα common core for 7PCD, and a QC-defined fixed 209-Cα kinase core for 8VB5. Because the broader 8U8X–8VB5 comparison was sensitive to activation-loop deviations, the fixed 209-Cα core was used for the final YVMA analysis. These comparisons were used solely as conformational-coverage diagnostics, not as evidence of thermodynamic or equilibrium populations.

S2. Supplementary Tables

Table S1. Pre-reaction HER2–inhibitor system and chemical-state validation.

All 14 pre-reaction systems retained an intact electrophilic double bond and an unlinked neutral cysteine thiol. Binary fields are reported as 1/0 from the master validation table.

System	Protein	Compound	Reactive Cys	Warhead atoms	Warhead bond order	Warhead bond length (Å)	Cys SG present	Cys HG present	Sγ–ligand bond present	Ligand formal charge	Charge matches Epik	Full system formal charge
WT-3	WT	3	Cys805	C5–C6	2	1.3309	True	True	False	1	True	0
WT-4a	WT	4a	Cys805	C5–C6	2	1.3578	True	True	False	1	True	0
WT-5a	WT	5a	Cys805	C31–C30	2	1.3689	True	True	False	1	True	0
WT-8c	WT	8c	Cys805	C5–C6	2	1.3974	True	True	False	1	True	0
WT-9c	WT	9c	Cys805	C5–C6	2	1.3731	True	True	False	1	True	0
WT-10e	WT	10e	Cys805	C27–C26	2	1.3491	True	True	False	1	True	0
WT-11f	WT	11f	Cys805	C27–C26	2	1.3259	True	True	False	1	True	0
YVMA-3	YVMA	3	Cys809	C5–C6	2	1.3466	True	True	False	1	True	0
YVMA-4a	YVMA	4a	Cys809	C5–C6	2	1.36	True	True	False	1	True	0
YVMA-5a	YVMA	5a	Cys809	C31–C30	2	1.3533	True	True	False	1	True	0
YVMA-8c	YVMA	8c	Cys809	C5–C6	2	1.3882	True	True	False	1	True	0
YVMA-9c	YVMA	9c	Cys809	C5–C6	2	1.3964	True	True	False	1	True	0
YVMA-10e	YVMA	10e	Cys809	C27–C26	2	1.3807	True	True	False	1	True	0
YVMA-11f	YVMA	11f	Cys809	C27–C26	2	1.3212	True	True	False	1	True	0

Table S2. Replica-level pre-reaction NAC-like geometry under the primary criterion.

Primary criterion: Sγ–Cβ distance ≤4.0 Å and Sγ–Cβ–Cα angle 90–125°. Each condition contains three independent 200 ns trajectories.

Protein	PDB	Compound	Seed	Frames	NAC-positive frames	P_NAC (%)	Distance cutoff (Å)	Angle range (°)
WT	7JXH	10e	2007	19998	110	0.550055	4	90–125
WT	7JXH	10e	3456	19998	223	1.1151	4	90–125
WT	7JXH	10e	6785	19998	607	3.0353	4	90–125
WT	7JXH	11f	2007	19998	255	1.2751	4	90–125
WT	7JXH	11f	3456	19998	229	1.1451	4	90–125
WT	7JXH	11f	6785	19998	908	4.5405	4	90–125
WT	7JXH	3	2007	19998	0	0	4	90–125
WT	7JXH	3	3456	19998	7	0.035004	4	90–125
WT	7JXH	3	6785	19998	6	0.030003	4	90–125
WT	7JXH	4a	2007	19998	492	2.4602	4	90–125
WT	7JXH	4a	3456	19998	1528	7.6408	4	90–125
WT	7JXH	4a	6785	19998	2471	12.356	4	90–125
WT	7JXH	5a	2007	19998	3000	15.002	4	90–125
WT	7JXH	5a	3456	19998	1347	6.7357	4	90–125
WT	7JXH	5a	6785	19998	337	1.6852	4	90–125
WT	7JXH	8c	2007	19998	62	0.310031	4	90–125
WT	7JXH	8c	3456	19998	345	1.7252	4	90–125
WT	7JXH	8c	6785	19998	127	0.635064	4	90–125
WT	7JXH	9c	2007	19998	63	0.315032	4	90–125
WT	7JXH	9c	3456	19998	3	0.015002	4	90–125
WT	7JXH	9c	6785	19998	149	0.745075	4	90–125
YVMA	8U8X	10e	2007	19998	433	2.1652	4	90–125
YVMA	8U8X	10e	3456	19998	591	2.9553	4	90–125
YVMA	8U8X	10e	6785	19998	1251	6.2556	4	90–125
YVMA	8U8X	11f	2007	19998	0	0	4	90–125
YVMA	8U8X	11f	3456	19998	1	0.005001	4	90–125
YVMA	8U8X	11f	6785	19998	0	0	4	90–125
YVMA	8U8X	3	2007	19998	400	2.0002	4	90–125
YVMA	8U8X	3	3456	19998	10	0.050005	4	90–125
YVMA	8U8X	3	6785	19998	37	0.185019	4	90–125
YVMA	8U8X	4a	2007	19998	986	4.9305	4	90–125
YVMA	8U8X	4a	3456	19998	562	2.8103	4	90–125
YVMA	8U8X	4a	6785	19998	24	0.120012	4	90–125
YVMA	8U8X	5a	2007	19998	2177	10.886	4	90–125
YVMA	8U8X	5a	3456	19998	1255	6.2756	4	90–125
YVMA	8U8X	5a	6785	19998	314	1.5702	4	90–125

YVMA	8U8X	8c	2007	19998	177	0.885089	4	90–125
YVMA	8U8X	8c	3456	19998	117	0.585059	4	90–125
YVMA	8U8X	8c	6785	19998	87	0.435044	4	90–125
YVMA	8U8X	9c	2007	19998	59	0.29503	4	90–125
YVMA	8U8X	9c	3456	19998	19	0.09501	4	90–125
YVMA	8U8X	9c	6785	19998	122	0.610061	4	90–125

Table S3. Condition-level pre-reaction NAC-like geometry summary.

Condition means and dispersion are calculated across the three independent trajectories; trajectory frames are not treated as independent replicates.

Protein	Compound	Seed 2007 P_NAC (%)	Seed 3456 P_NAC (%)	Seed 6785 P_NAC (%)	Mean P_NAC (%)	SD (%)	Median (%)	Min (%)	Max (%)	Frames/replica	n replicas	Primary criterion
WT	3	0	0.035004	0.030003	0.021669	0.018932	0.030003	0	0.035004	19998	3	d≤4 Å; angle 90–125°
WT	4a	2.4602	7.6408	12.356	7.4857	4.9498	7.6408	2.4602	12.356	19998	3	d≤4 Å; angle 90–125°
WT	5a	15.002	6.7357	1.6852	7.8074	6.7226	6.7357	1.6852	15.002	19998	3	d≤4 Å; angle 90–125°
WT	8c	0.310031	1.7252	0.635064	0.890089	0.741239	0.635064	0.310031	1.7252	19998	3	d≤4 Å; angle 90–125°
WT	9c	0.315032	0.015002	0.745075	0.358369	0.366961	0.315032	0.015002	0.745075	19998	3	d≤4 Å; angle 90–125°
WT	10e	0.550055	1.1151	3.0353	1.5668	1.3027	1.1151	0.550055	3.0353	19998	3	d≤4 Å; angle 90–125°
WT	11f	1.2751	1.1451	4.5405	2.3202	1.9239	1.2751	1.1451	4.5405	19998	3	d≤4 Å; angle 90–125°
YVMA	3	2.0002	0.050005	0.185019	0.745075	1.0891	0.185019	0.050005	2.0002	19998	3	d≤4 Å; angle 90–125°
YVMA	4a	4.9305	2.8103	0.120012	2.6203	2.4109	2.8103	0.120012	4.9305	19998	3	d≤4 Å; angle 90–125°
YVMA	5a	10.886	6.2756	1.5702	6.244	4.658	6.2756	1.5702	10.886	19998	3	d≤4 Å; angle 90–125°
YVMA	8c	0.885089	0.585059	0.435044	0.635064	0.229152	0.585059	0.435044	0.885089	19998	3	d≤4 Å; angle 90–125°
YVMA	9c	0.29503	0.09501	0.610061	0.333367	0.259657	0.29503	0.09501	0.610061	19998	3	d≤4 Å; angle 90–125°
YVMA	10e	2.1652	2.9553	6.2556	3.792	2.1698	2.9553	2.1652	6.2556	19998	3	d≤4 Å; angle 90–125°
YVMA	11f	0	0.005001	0	0.001667	0.002887	0	0	0.005001	19998	3	d≤4 Å; angle 90–125°

Table S4. Six-cutoff sensitivity analysis of pre-reaction NAC-like geometry.

Sensitivity matrix uses distance thresholds of 3.5, 4.0 and 4.5 Å combined with angle windows of 90–125° and 95–120°. Values are not independent validation experiments.

Protein	Compound	Cutoff	Seed 2007 P_NAC (%)	Seed 3456 P_NAC (%)	Seed 6785 P_NAC (%)	Mean P_NAC (%)	SD (%)	n replicas
WT	3	3.5 Å / 90–125°	0	0	0.005001	0.001667	0.002887	3
WT	3	3.5 Å / 95–120°	0	0	0	0	0	3
WT	3	4.0 Å / 90–125°	0	0.035004	0.030003	0.021669	0.018932	3
WT	3	4.0 Å / 95–120°	0	0.020002	0.010001	0.010001	0.010001	3
WT	3	4.5 Å / 90–125°	0	0.105011	0.10001	0.06834	0.059237	3
WT	3	4.5 Å / 95–120°	0	0.065007	0.040004	0.035004	0.03279	3
WT	4a	3.5 Å / 90–125°	0.220022	1.1101	1.5002	0.943428	0.65614	3
WT	4a	3.5 Å / 95–120°	0.180018	0.70007	1.1501	0.676734	0.485469	3
WT	4a	4.0 Å / 90–125°	2.4602	7.6408	12.356	7.4857	4.9498	3
WT	4a	4.0 Å / 95–120°	1.9902	5.2955	9.1559	5.4805	3.5864	3
WT	4a	4.5 Å / 90–125°	4.5505	12.566	20.097	12.405	7.7745	3
WT	4a	4.5 Å / 95–120°	3.5854	9.0359	14.641	9.0876	5.5282	3
WT	5a	3.5 Å / 90–125°	0.725073	0.285029	0.090009	0.366703	0.325314	3
WT	5a	3.5 Å / 95–120°	0.545055	0.190019	0.070007	0.26836	0.247023	3
WT	5a	4.0 Å / 90–125°	15.002	6.7357	1.6852	7.8074	6.7226	3
WT	5a	4.0 Å / 95–120°	11.626	4.9655	1.3101	5.9673	5.2305	3
WT	5a	4.5 Å / 90–125°	25.823	12.566	4.3854	14.258	10.818	3
WT	5a	4.5 Å / 95–120°	19.487	9.0509	3.2653	10.601	8.2212	3
WT	8c	3.5 Å / 90–125°	0.005001	0.265027	0.035004	0.101677	0.142258	3
WT	8c	3.5 Å / 95–120°	0.005001	0.170017	0.015002	0.06334	0.092521	3
WT	8c	4.0 Å / 90–125°	0.310031	1.7252	0.635064	0.890089	0.741239	3
WT	8c	4.0 Å / 95–120°	0.145015	1.0801	0.450045	0.558389	0.476869	3
WT	8c	4.5 Å / 90–125°	1.7852	3.1503	1.6752	2.2036	0.821762	3
WT	8c	4.5 Å / 95–120°	0.945095	2.0702	1.3151	1.4435	0.573432	3
WT	9c	3.5 Å / 90–125°	0.040004	0	0.040004	0.026669	0.023096	3
WT	9c	3.5 Å / 95–120°	0.020002	0	0.030003	0.016668	0.015277	3
WT	9c	4.0 Å / 90–125°	0.315032	0.015002	0.745075	0.358369	0.366961	3
WT	9c	4.0 Å / 95–120°	0.205021	0	0.540054	0.248358	0.272623	3
WT	9c	4.5 Å / 90–125°	1.0251	0.035004	2.9503	1.3368	1.4824	3
WT	9c	4.5 Å / 95–120°	0.70007	0.005001	2.3752	1.0268	1.2184	3
WT	10e	3.5 Å / 90–125°	0.025003	0.015002	0.225023	0.088342	0.118474	3
WT	10e	3.5 Å / 95–120°	0.015002	0.015002	0.160016	0.06334	0.083724	3
WT	10e	4.0 Å / 90–125°	0.550055	1.1151	3.0353	1.5668	1.3027	3
WT	10e	4.0 Å / 95–120°	0.315032	0.745075	2.0702	1.0434	0.914836	3

WT	10e	4.5 Å / 90–125°	1.6952	2.3952	6.2656	3.452	2.4617	3
WT	10e	4.5 Å / 95–120°	1.0451	1.5502	4.5555	2.3836	1.8978	3
WT	11f	3.5 Å / 90–125°	0.060006	0.030003	0.245025	0.111678	0.116452	3
WT	11f	3.5 Å / 95–120°	0.030003	0.030003	0.19502	0.085009	0.095272	3
WT	11f	4.0 Å / 90–125°	1.2751	1.1451	4.5405	2.3202	1.9239	3
WT	11f	4.0 Å / 95–120°	0.890089	0.820082	3.1403	1.6168	1.3198	3
WT	11f	4.5 Å / 90–125°	3.1553	2.6303	9.1059	4.9638	3.5967	3
WT	11f	4.5 Å / 95–120°	2.1602	1.9202	6.0256	3.3687	2.3041	3
YVMA	3	3.5 Å / 90–125°	0.255026	0	0.055006	0.103344	0.134209	3
YVMA	3	3.5 Å / 95–120°	0.175018	0	0.030003	0.06834	0.093595	3
YVMA	3	4.0 Å / 90–125°	2.0002	0.050005	0.185019	0.745075	1.0891	3
YVMA	3	4.0 Å / 95–120°	1.2551	0.040004	0.075008	0.456712	0.691668	3
YVMA	3	4.5 Å / 90–125°	3.0753	0.115012	0.285029	1.1584	1.6622	3
YVMA	3	4.5 Å / 95–120°	2.0602	0.075008	0.125013	0.753409	1.132	3
YVMA	4a	3.5 Å / 90–125°	0.715072	0.355036	0.005001	0.358369	0.355047	3
YVMA	4a	3.5 Å / 95–120°	0.470047	0.260026	0	0.243358	0.235466	3
YVMA	4a	4.0 Å / 90–125°	4.9305	2.8103	0.120012	2.6203	2.4109	3
YVMA	4a	4.0 Å / 95–120°	3.0303	1.8702	0.060006	1.6535	1.497	3
YVMA	4a	4.5 Å / 90–125°	7.3757	4.5805	0.390039	4.1154	3.516	3
YVMA	4a	4.5 Å / 95–120°	4.5105	2.9253	0.245025	2.5603	2.156	3
YVMA	5a	3.5 Å / 90–125°	0.640064	0.575058	0.09501	0.43671	0.297701	3
YVMA	5a	3.5 Å / 95–120°	0.510051	0.475048	0.075008	0.353369	0.241702	3
YVMA	5a	4.0 Å / 90–125°	10.886	6.2756	1.5702	6.244	4.658	3
YVMA	5a	4.0 Å / 95–120°	8.5409	4.5255	1.1151	4.7271	3.717	3
YVMA	5a	4.5 Å / 90–125°	15.757	11.156	3.2853	10.066	6.3067	3
YVMA	5a	4.5 Å / 95–120°	12.001	7.5758	2.2752	7.2841	4.8695	3
YVMA	8c	3.5 Å / 90–125°	0.035004	0.035004	0.020002	0.030003	0.008661	3
YVMA	8c	3.5 Å / 95–120°	0.020002	0.010001	0.015002	0.015002	0.005001	3
YVMA	8c	4.0 Å / 90–125°	0.885089	0.585059	0.435044	0.635064	0.229152	3
YVMA	8c	4.0 Å / 95–120°	0.630063	0.270027	0.220022	0.373371	0.223704	3
YVMA	8c	4.5 Å / 90–125°	2.4902	1.9802	0.930093	1.8002	0.795504	3
YVMA	8c	4.5 Å / 95–120°	1.8552	1.0901	0.505051	1.1501	0.677065	3
YVMA	9c	3.5 Å / 90–125°	0.030003	0.005001	0.060006	0.03167	0.027541	3
YVMA	9c	3.5 Å / 95–120°	0.005001	0.005001	0.025003	0.011668	0.011548	3
YVMA	9c	4.0 Å / 90–125°	0.29503	0.09501	0.610061	0.333367	0.259657	3
YVMA	9c	4.0 Å / 95–120°	0.140014	0.050005	0.290029	0.160016	0.121256	3
YVMA	9c	4.5 Å / 90–125°	1.2801	0.365037	1.3101	0.985099	0.537199	3
YVMA	9c	4.5 Å / 95–120°	0.805081	0.185019	0.660066	0.550055	0.324339	3
YVMA	10e	3.5 Å / 90–125°	0.10001	0.120012	0.260026	0.160016	0.087187	3
YVMA	10e	3.5 Å / 95–120°	0.065007	0.09501	0.170017	0.110011	0.054089	3
YVMA	10e	4.0 Å / 90–125°	2.1652	2.9553	6.2556	3.792	2.1698	3

YVMA	10e	4.0 Å / 95–120°	0.955096	2.1102	3.4103	2.1585	1.2283	3
YVMA	10e	4.5 Å / 90–125°	3.7854	6.1656	12.486	7.4791	4.4967	3
YVMA	10e	4.5 Å / 95–120°	1.6602	4.4004	6.2706	4.1104	2.3189	3
YVMA	11f	3.5 Å / 90–125°	0	0	0	0	0	3
YVMA	11f	3.5 Å / 95–120°	0	0	0	0	0	3
YVMA	11f	4.0 Å / 90–125°	0	0.005001	0	0.001667	0.002887	3
YVMA	11f	4.0 Å / 95–120°	0	0	0	0	0	3
YVMA	11f	4.5 Å / 90–125°	0	0.035004	0	0.011668	0.020209	3
YVMA	11f	4.5 Å / 95–120°	0	0.020002	0	0.006667	0.011548	3

Threshold mean P_NAC wide view

Protein	Compound	3.5 Å / 90–125°	4.0 Å / 95–120°	4.5 Å / 90–125°	4.5 Å / 95–120°
WT	3	0.001667	0.010001	0.06834	0.035004
WT	4a	0.943428	5.4805	12.405	9.0876
WT	5a	0.366703	5.9673	14.258	10.601
WT	8c	0.101677	0.558389	2.2036	1.4435
WT	9c	0.026669	0.248358	1.3368	1.0268
WT	10e	0.088342	1.0434	3.452	2.3836
WT	11f	0.111678	1.6168	4.9638	3.3687
YVMA	3	0.103344	0.456712	1.1584	0.753409
YVMA	4a	0.358369	1.6535	4.1154	2.5603
YVMA	5a	0.43671	4.7271	10.066	7.2841
YVMA	8c	0.030003	0.373371	1.8002	1.1501
YVMA	9c	0.03167	0.160016	0.985099	0.550055
YVMA	10e	0.160016	2.1585	7.4791	4.1104
YVMA	11f	0	0	0.011668	0.006667

Table S5. Distance–angle joint-density coverage and YVMA–11f rare-event summary.

Section A reports histogram coverage for the conditions with stored 20×20 distance–angle joint densities. Section B reports the YVMA–11f rare-event replica summary; one isolated positive frame occurred in seed 3456. Section C reports the starting-conformation sensitivity analysis for YVMA–11f using an alternative chemically valid ligand conformation.

Section A. Distance–angle joint-density coverage summary.

System	Protein	Compound	Frames	Distance bins	Angle bins	Cells	Summed frame count
WT-8c	7JXH	8c	59994	20	20	400	59994
WT-9c	7JXH	9c	59994	20	20	400	59994
WT-10e	7JXH	10e	59994	20	20	400	59994
WT-11f	7JXH	11f	59994	20	20	400	59994
YVMA-8c	8U8X	8c	59994	20	20	400	59994
YVMA-9c	8U8X	9c	59994	20	20	400	59994
YVMA-10e	8U8X	10e	59994	20	20	400	59994
YVMA-11f	8U8X	11f	59994	20	20	400	59994

Section B. YVMA–11f rare-event replica summary

System	Replica	Total frames	Positive NAC frames	Episode count	Single-frame event	Longest episode (frames)	Total positive duration (frames)	Interpretation
YVMA-11f	2007	19998	0	0	n.a.	0	0	no persistent NAC episode observed
YVMA-11f	3456	19998	1	1	YES	1	1	no persistent NAC episode observed
YVMA-11f	6785	19998	0	0	n.a.	0	0	no persistent NAC episode observed
YVMA-9c	2007	19998	59	50	NO	3	59	detectable but rare NAC events
YVMA-9c	3456	19998	19	16	NO	2	19	detectable but rare NAC events
YVMA-9c	6785	19998	122	100	NO	6	122	detectable but rare NAC events

Section C. HER2YVMA–11f starting-conformation sensitivity analysis

Starting conformation	Replica	Total frames	Positive NAC frames	P_NAC (%)	Episode count	Single-frame episodes	Longest episode (frames)	Total positive duration (frames)	Interpretation
Original	2007	19,998	0	0.0000	0	0	0	0	No NAC-like event observed
Original	3456	19,998	1	0.0050	1	1	1	1	Isolated single-frame NAC-like event
Original	6785	19,998	0	0.0000	0	0	0	0	No NAC-like event observed
**Original, pooled**	—	**59,994**	**1**	**0.0017**	**1**	**1**	**1**	**1**	**NAC-like geometry was rarely sampled**
Alternative	2007	19,998	1,140	5.7006	634	380	10	1,140	Recurrent NAC-like sampling
Alternative	3456	19,998	1,450	7.2507	750	428	13	1,450	Recurrent NAC-like sampling
Alternative	6785	19,998	1,080	5.4005	614	408	14	1,080	Recurrent NAC-like sampling
**Alternative, pooled**	—	**59,994**	**3,670**	**6.1173**	**1,998**	**1,216**	**14**	**3,670**	**Markedly increased NAC-like accessibility from the alternative starting state**

Note: NAC-like frames were defined using the primary geometric criterion of Sγ–Cβ distance ≤ 4.0 Å and Sγ–Cβ–Cα angle of 90–125°. The original and alternative simulations used two chemically valid 11f starting conformations consistent with the experimentally synthesized stereochemical structure. Each starting state was evaluated using three independent 200-ns replicas. Episode counts were defined as contiguous runs of NAC-positive frames within each replica; pooled episode counts represent the sum across the three replicas rather than continuity across trajectories. The alternative starting conformation increased the pooled NAC-like population from 1/59,994 frames (0.0017%) to 3,670/59,994 frames (6.1173%), demonstrating substantial starting-conformation sensitivity. Accordingly, the original low NAC population is interpreted as conditional on the sampled starting conformational basin rather than as evidence for a general absence of reaction-compatible conformations.

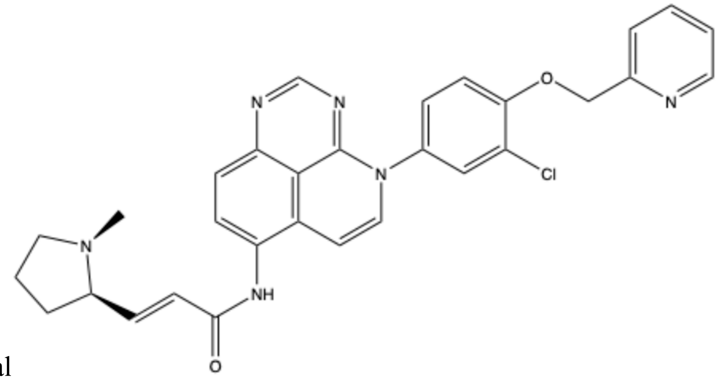

Original

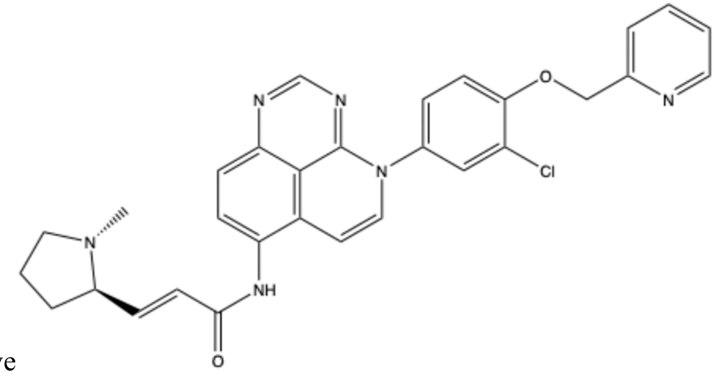

Alternative

Table S6. Condition-level OC2-derived structural descriptors and higher-resolution k=5 sampled representation.

Section A reports condition-level continuous descriptors and S1–S5 assignment fractions for the Equal-N = 1000 sampled representation used as a sample-size sensitivity analysis. Section B reports the corresponding condition-level descriptors and S1–S5 assignment fractions calculated from all retained OC2-generated covalently linked structures. Continuous descriptors are the primary structural readouts, whereas S1–S5 assignments are retained only as a higher-resolution operational representation and are not interpreted as thermodynamic states or equilibrium populations.

Section A. Equal-N = 1000 condition-level descriptor and k = 5 representation

Protein/PDB	Compound	Equal-N	DFG RMSD median (Å)	αC RMSD median (Å)	Lys51–Glu68 min distance median (Å)	P-loop RMSD median (Å)	C5-shell min distance median (Å)	Mapped hinge atom	Mapped hinge median (Å)	S1 (%)	S2 (%)	S3 (%)	S4 (%)	S5 (%)
7JXH	10e	1000	1.5222	3.1232	7.2557	2.3425	3.7626	N37	3.0435	50.8	5.3	6.3	6.8	30.8
7JXH	11f	1000	1.4572	3.1785	7.6973	2.3271	3.6997	N37	3.0375	47	5.6	4.2	8.3	34.9
7JXH	3	1000	1.5504	2.9896	7.4416	2.1898	3.9425	N17	3.0509	52.4	5.2	5.3	8.8	28.3
7JXH	4a	1000	1.6176	2.97	6.1232	2.3496	3.4954	N22	3.0695	52.5	6.3	9.3	7.5	24.4
7JXH	5a	1000	1.6009	3.0441	6.4257	2.427	3.499	N41	3.0695	48.6	7.7	9.5	6.6	27.6
7JXH	8c	1000	1.529	3.0163	6.889	2.3548	3.9255	N16	3.0485	52.4	5.3	5.8	7.1	29.4
7JXH	9c	1000	1.4945	3.1479	8.0835	2.26	3.7846	N16	3.0426	48.2	5.3	4.4	9.4	32.7
8U8X	10e	1000	1.1852	3.911	3.3052	2.8018	3.7638	N37	3.059	47.8	25.8	8.2	1.6	16.6
8U8X	11f	1000	1.1415	4.0198	3.4935	2.7243	3.7212	N37	3.0357	48.3	24.1	5.7	1.9	20
8U8X	3	1000	1.1152	3.8669	3.1921	2.6249	3.9433	N17	3.0488	57.2	21.8	7.1	1	12.9
8U8X	4a	1000	1.1765	4.0026	3.1309	2.8136	3.516	N22	3.0762	48.3	27.6	12.2	1.8	10.1
8U8X	5a	1000	1.2044	3.9196	3.1316	2.8646	3.4982	N41	3.0861	49.6	25.5	13.1	1.6	10.2
8U8X	8c	1000	1.1894	3.9194	3.327	2.8371	3.9287	N16	3.0562	48.6	24.8	9.3	1.4	15.9
8U8X	9c	1000	1.1381	4.026	3.4897	2.7385	3.8505	N16	3.0427	49.4	23.5	7	1.6	18.5

Section B. Full-data condition-level descriptors and k = 5 representation

Protein/PDB	Compound	Full-data N	DFG RMSD mean (Å)	αC RMSD mean (Å)	Lys51–Glu68 min-distance mean (Å)	P-loop RMSD mean (Å)	C5-shell min-distance mean (Å)	Mapped hinge atom	Mapped hinge median (Å)	S1 (%)	S2 (%)	S3 (%)	S4 (%)	S5 (%)
7JXH	3	1072	1.6296	3.2032	7.8259	2.3389	3.9541	N17	3.0502	52.146	5.317	5.224	9.049	28.265
7JXH	4a	1121	1.6560	3.1373	7.1086	2.5060	3.4929	N22	3.0704	53.524	6.601	8.831	7.136	23.907
7JXH	5a	1096	1.6596	3.2210	7.2153	2.5332	3.5032	N41	3.0684	49.270	7.847	9.580	6.569	26.734
7JXH	8c	1079	1.6154	3.2565	7.5302	2.4876	3.9305	N16	3.0480	51.993	5.468	5.931	7.044	29.564
7JXH	9c	1147	1.6105	3.3229	8.1257	2.4073	3.8321	N16	3.0429	47.777	5.580	4.446	9.329	32.868
7JXH	10e	1814	1.5973	3.2773	7.7019	2.4696	3.7729	N37	3.0465	51.047	6.395	5.954	6.781	29.824
7JXH	11f	1765	1.5599	3.3481	8.0372	2.4259	3.6902	N37	3.0374	48.669	5.609	4.363	7.195	34.164
8U8X	3	1109	1.1862	4.0334	4.4005	2.6613	3.9480	N17	3.0485	57.980	21.190	6.853	0.902	13.075
8U8X	4a	1131	1.2532	4.1105	4.0615	2.8353	3.5141	N22	3.0758	49.160	27.144	12.113	1.768	9.814
8U8X	5a	1087	1.2549	4.1100	4.0817	2.8976	3.5046	N41	3.0833	49.678	25.391	13.155	1.564	10.212
8U8X	8c	1094	1.2454	4.1005	4.8853	2.8556	3.9437	N16	3.0544	48.720	24.863	8.958	1.554	15.905
8U8X	9c	1097	1.2066	4.1388	5.0543	2.7842	3.8708	N16	3.0438	49.499	23.245	6.746	1.641	18.870
8U8X	10e	1818	1.2166	4.1161	4.9336	2.8791	3.7746	N37	3.0567	46.645	25.743	8.526	1.430	17.657
8U8X	11f	1708	1.2013	4.2526	5.3043	2.8501	3.7288	N37	3.0399	46.604	24.941	6.792	1.581	20.082

Table S7. Matched-perturbation changes in the k=5 higher-resolution operational representation.

ΔP is the within-background change in sampled representation; Γ = ΔP_YVMA − ΔP_WT. These quantities are descriptive distribution-level measures, not free-energy terms.

Family	Role	Transition	Operational state	WT base (%)	WT test (%)	WT ΔP (pp)	YVMA base (%)	YVMA test (%)	YVMA ΔP (pp)	Γ (pp)
Pocket	primary	3→4a	S1	52.4	52.5	0.1	57.2	48.3	-8.9	-9
Pocket	primary	3→4a	S2	5.2	6.3	1.1	21.8	27.6	5.8	4.7
Pocket	primary	3→4a	S3	5.3	9.3	4	7.1	12.2	5.1	1.1
Pocket	primary	3→4a	S4	8.8	7.5	-1.3	1	1.8	0.8	2.1
Pocket	primary	3→4a	S5	28.3	24.4	-3.9	12.9	10.1	-2.8	1.1
Cyclization	primary	3→8c	S1	52.4	52.4	0	57.2	48.6	-8.6	-8.6
Cyclization	primary	3→8c	S2	5.2	5.3	0.1	21.8	24.8	3	2.9
Cyclization	primary	3→8c	S3	5.3	5.8	0.5	7.1	9.3	2.2	1.7
Cyclization	primary	3→8c	S4	8.8	7.1	-1.7	1	1.4	0.4	2.1
Cyclization	primary	3→8c	S5	28.3	29.4	1.1	12.9	15.9	3	1.9
Cyclization	primary	3→9c	S1	52.4	48.2	-4.2	57.2	49.4	-7.8	-3.6
Cyclization	primary	3→9c	S2	5.2	5.3	0.1	21.8	23.5	1.7	1.6
Cyclization	primary	3→9c	S3	5.3	4.4	-0.9	7.1	7	-0.1	0.8
Cyclization	primary	3→9c	S4	8.8	9.4	0.6	1	1.6	0.6	-0
Cyclization	primary	3→9c	S5	28.3	32.7	4.4	12.9	18.5	5.6	1.2
Tail	primary	4a→5a	S1	52.5	48.6	-3.9	48.3	49.6	1.3	5.2
Tail	primary	4a→5a	S2	6.3	7.7	1.4	27.6	25.5	-2.1	-3.5
Tail	primary	4a→5a	S3	9.3	9.5	0.2	12.2	13.1	0.9	0.7
Tail	primary	4a→5a	S4	7.5	6.6	-0.9	1.8	1.6	-0.2	0.7
Tail	primary	4a→5a	S5	24.4	27.6	3.2	10.1	10.2	0.1	-3.1
Tail	primary	8c→10e	S1	52.4	50.8	-1.6	48.6	47.8	-0.8	0.8
Tail	primary	8c→10e	S2	5.3	5.3	0	24.8	25.8	1	1
Tail	primary	8c→10e	S3	5.8	6.3	0.5	9.3	8.2	-1.1	-1.6
Tail	primary	8c→10e	S4	7.1	6.8	-0.3	1.4	1.6	0.2	0.5
Tail	primary	8c→10e	S5	29.4	30.8	1.4	15.9	16.6	0.7	-0.7
Tail	primary	9c→11f	S1	48.2	47	-1.2	49.4	48.3	-1.1	0.1
Tail	primary	9c→11f	S2	5.3	5.6	0.3	23.5	24.1	0.6	0.3
Tail	primary	9c→11f	S3	4.4	4.2	-0.2	7	5.7	-1.3	-1.1
Tail	primary	9c→11f	S4	9.4	8.3	-1.1	1.6	1.9	0.3	1.4
Tail	primary	9c→11f	S5	32.7	34.9	2.2	18.5	20	1.5	-0.7
Composite	secondary	4a→8c	S1	52.5	52.4	-0.1	48.3	48.6	0.3	0.4
Composite	secondary	4a→8c	S2	6.3	5.3	-1	27.6	24.8	-2.8	-1.8
Composite	secondary	4a→8c	S3	9.3	5.8	-3.5	12.2	9.3	-2.9	0.6
Composite	secondary	4a→8c	S4	7.5	7.1	-0.4	1.8	1.4	-0.4	0
Composite	secondary	4a→8c	S5	24.4	29.4	5	10.1	15.9	5.8	0.8

Table S8. Higher-*k* sensitivity analysis of APO WT HER2 descriptor profiles.

Descriptor profiles are shown for the k = 5–8 partitions of the same 918 APO WT HER2 structures used to define the shared coordinate. N is the number of structures assigned to each cluster and fraction = N/918. For k = 5, the operational groups are denoted S1–S5; k = 6–8 clusters are shown as higher-resolution sensitivity partitions. The four descriptor columns report within-cluster medians in Å. These higher-k partitions were used only for sensitivity analysis and were not interpreted as additional thermodynamic states.

k	Cluster	N	Fraction	DFG RMSD median (Å)	αC RMSD median (Å)	Lys51–Glu68 min distance median (Å)	P-loop RMSD median (Å)
5	S1	221	0.240740741	1.180731401	2.058021074	3.291124276	2.212953915
5	S2	291	0.316993464	1.256421096	2.513877038	3.31632417	3.692794659
5	S3	154	0.167755991	2.056559095	3.2042015	4.797408922	4.793421693
5	S4	92	0.100217865	3.328251839	4.701405987	10.30943871	2.86737097
5	S5	160	0.174291939	1.540892539	4.878016587	12.69831096	2.961755978
6	0	132	0.14379085	2.035215346	2.580031721	4.216827344	3.188220708
6	1	251	0.273420479	1.204939491	2.567758919	3.228645227	3.850650758
6	2	89	0.096949891	3.329485903	4.83098387	10.49452495	2.886769431
6	3	113	0.123093682	1.873477581	3.253279612	5.078584252	5.159011378
6	4	176	0.191721133	1.046276612	1.891608527	3.004360735	2.207277798
6	5	157	0.171023965	1.538137476	4.936367351	12.71989155	2.968142456
7	0	81	0.088235294	3.38809303	4.859255783	10.36372896	2.886769431
7	1	172	0.187363834	1.041273132	1.858195665	2.987036284	2.219087625
7	2	112	0.122004357	1.859581903	3.252392125	5.018087852	5.152734786
7	3	66	0.071895425	1.542188324	6.086708581	13.54209438	3.603054027
7	4	249	0.27124183	1.203589787	2.563970999	3.228645227	3.850650758
7	5	114	0.124183007	1.579541309	4.274058558	11.43784421	2.516601577
7	6	124	0.135076253	2.057437147	2.546278131	4.010340439	3.231458595
8	0	115	0.125272331	1.580710774	4.274991297	11.49604054	2.51747728
8	1	114	0.124183007	2.037497663	2.580031721	3.904975869	3.137065265
8	2	215	0.234204793	1.163767176	2.479700736	3.218230259	3.586524808
8	3	80	0.087145969	3.384888198	4.845119827	10.42912696	2.86737097
8	4	28	0.030501089	2.250112579	3.719939776	5.585398001	6.289400036
8	5	146	0.159041394	1.691406172	2.910956617	4.383717751	4.679327054
8	6	64	0.069716776	1.539097994	6.098039232	13.54209438	3.603054027
8	7	156	0.169934641	1.021442173	1.852243024	2.960148516	2.159388683

Table S9. Repeated-resampling robustness of OC2-derived continuous structural responses.

The master dataset contains repeated-resampling summaries for all six primary matched transformations: 3→4a, 3→8c, 3→9c, 4a→5a, 8c→10e, and 9c→11f. Direction retention reports the fraction of resamples retaining the dominant Γ direction.

Transition	Descriptor	Resampling iterations	Mean Δ WT	SD Δ WT	Mean Δ YVMA	SD Δ YVMA	Mean Γ	Median Γ	Γ SD	Γ 2.5%	Γ 97.5%	Dominant direction	Direction retention
**3→4a**	**DFG_RMSD_ref_A**	1000	0.065147	0.011628	0.062430	0.006601	-0.002717	-0.003058	0.013626	-0.029815	0.023694	decrease	0.602000
**3→4a**	**alphaC_RMSD_ref_A**	1000	-0.062610	0.018699	0.136925	0.017376	0.199535	0.199766	0.025887	0.147738	0.249837	increase	1.000000
**3→4a**	**Lys51_Glu68_min_distance_A**	1000	-1.317673	0.061518	-0.054124	0.018974	1.263549	1.262027	0.064893	1.133555	1.398211	increase	1.000000
**3→4a**	**Ploop_RMSD_ref_A**	1000	0.179144	0.025853	0.210313	0.016535	0.031169	0.032218	0.030867	-0.028782	0.086900	increase	0.825000
**3→4a**	**C5_shell_min_distance_A**	1000	-0.432834	0.007162	-0.422645	0.006662	0.010189	0.010038	0.009902	-0.007867	0.029825	increase	0.824000
**3→4a**	**hinge_MetN_to_ligand_acceptor_A**	1000	0.020310	0.001592	0.027230	0.001651	0.006920	0.007053	0.002338	0.002064	0.011224	increase	0.997000
**4a→5a**	**DFG_RMSD_ref_A**	1000	-0.015012	0.012126	0.033732	0.007518	0.048744	0.048815	0.014231	0.021913	0.075910	increase	0.999000
**4a→5a**	**alphaC_RMSD_ref_A**	1000	0.101149	0.022127	-0.082744	0.017108	-0.183893	-0.183040	0.027550	-0.238682	-0.130216	decrease	1.000000
**4a→5a**	**Lys51_Glu68_min_distance_A**	1000	0.211204	0.071015	0.007495	0.013051	-0.203708	-0.206083	0.072731	-0.340862	-0.048560	decrease	0.994000
**4a→5a**	**Ploop_RMSD_ref_A**	1000	0.051589	0.026334	0.048680	0.018889	-0.002909	-0.003529	0.031452	-0.061279	0.059022	decrease	0.531000
**4a→5a**	**C5_shell_min_distance_A**	1000	-0.003963	0.004228	-0.005707	0.005454	-0.001744	-0.002031	0.006999	-0.015054	0.011897	decrease	0.599000
**4a→5a**	**hinge_MetN_to_ligand_acceptor_A**	1000	-0.002373	0.002320	0.008061	0.002246	0.010434	0.010248	0.003215	0.004287	0.016879	increase	1.000000
**3→8c**	**DFG_RMSD_ref_A**	1000	-0.019991	0.009613	0.078126	0.007394	0.098117	0.098218	0.012172	0.074955	0.122377	increase	1.000000
**3→8c**	**alphaC_RMSD_ref_A**	1000	0.012393	0.015662	0.052781	0.018637	0.040388	0.040429	0.024577	-0.006849	0.089805	increase	0.948000
**3→8c**	**Lys51_Glu68_min_distance_A**	1000	-0.529801	0.072271	0.157894	0.028894	0.687695	0.680298	0.078206	0.538087	0.853249	increase	1.000000
**3→8c**	**Ploop_RMSD_ref_A**	1000	0.151526	0.015624	0.229186	0.016935	0.077660	0.078061	0.022783	0.031892	0.120289	increase	0.999000

Transition	Descriptor	Resampling iterations	Mean Δ WT	SD Δ WT	Mean Δ YVMA	SD Δ YVMA	Mean Γ	Median Γ	Γ SD	Γ 2.5%	Γ 97.5%	Dominant direction	Direction retention
**3→8c**	**C5_shell_min_distance_A**	1000	-0.014291	0.006828	-0.006803	0.005905	0.007488	0.007550	0.008983	-0.009423	0.024402	increase	0.784000
**3→8c**	**hinge_MetN_to_ligand_acceptor_A**	1000	-0.002202	0.000908	0.005914	0.001561	0.008117	0.008131	0.001775	0.004637	0.011516	increase	1.000000
**3→9c**	**DFG_RMSD_ref_A**	1000	-0.037395	0.011778	0.023050	0.006659	0.060445	0.061809	0.013586	0.027146	0.083155	increase	1.000000
**3→9c**	**alphaC_RMSD_ref_A**	1000	0.161584	0.019158	0.169954	0.016748	0.008370	0.008480	0.026011	-0.042528	0.056799	increase	0.639000
**3→9c**	**Lys51_Glu68_min_distance_A**	1000	0.708180	0.107201	0.309868	0.022474	-0.398313	-0.403792	0.110450	-0.585077	-0.170777	decrease	1.000000
**3→9c**	**Ploop_RMSD_ref_A**	1000	0.069509	0.014270	0.134147	0.016241	0.064638	0.064934	0.021767	0.017460	0.103977	increase	0.995000
**3→9c**	**C5_shell_min_distance_A**	1000	-0.142362	0.008645	-0.084388	0.006280	0.057974	0.057865	0.010936	0.036407	0.080481	increase	1.000000
**3→9c**	**hinge_MetN_to_ligand_acceptor_A**	1000	-0.007192	0.000986	-0.004536	0.001294	0.002656	0.002705	0.001644	-0.000619	0.005881	increase	0.946000
**8c→10e**	**DFG_RMSD_ref_A**	1000	-0.013051	0.016768	-0.029303	0.010265	-0.016253	-0.016184	0.020131	-0.056801	0.022044	decrease	0.784000
**8c→10e**	**alphaC_RMSD_ref_A**	1000	0.080781	0.024755	0.033868	0.031561	-0.046913	-0.047843	0.040885	-0.123492	0.034452	decrease	0.876000
**8c→10e**	**Lys51_Glu68_min_distance_A**	1000	0.285932	0.132158	0.035733	0.054266	-0.250199	-0.256759	0.142320	-0.527414	0.027089	decrease	0.960000
**8c→10e**	**Ploop_RMSD_ref_A**	1000	-0.006202	0.027882	-0.001646	0.032928	0.004555	0.004533	0.044254	-0.078839	0.096726	increase	0.544000
**8c→10e**	**C5_shell_min_distance_A**	1000	-0.154527	0.006714	-0.161729	0.007413	-0.007202	-0.007193	0.010009	-0.027606	0.012224	decrease	0.760000
**8c→10e**	**hinge_MetN_to_ligand_acceptor_A**	1000	-0.001574	0.002250	0.002313	0.002482	0.003887	0.003674	0.003310	-0.001964	0.010303	increase	0.881000
**9c→11f**	**DFG_RMSD_ref_A**	1000	-0.044583	0.016037	0.011747	0.011425	0.056330	0.056625	0.019586	0.017110	0.095777	increase	0.996000
**9c→11f**	**alphaC_RMSD_ref_A**	1000	-0.005510	0.042368	0.020041	0.034503	0.025551	0.025665	0.053901	-0.080284	0.136458	increase	0.683000
**9c→11f**	**Lys51_Glu68_min_distance_A**	1000	-0.503192	0.146217	0.033081	0.061352	0.536272	0.545367	0.160309	0.204207	0.827569	increase	1.000000
**9c→11f**	**Ploop_RMSD_ref_A**	1000	0.035541	0.029086	0.042155	0.030565	0.006615	0.004855	0.041621	-0.071406	0.088474	increase	0.548000
**9c→11f**	**C5_shell_min_distance_A**	1000	-0.103769	0.010605	-0.127079	0.008110	-0.023309	-0.022799	0.013118	-0.048490	0.001593	decrease	0.962000

Transition	Descriptor	Resampling iterations	Mean Δ WT	SD Δ WT	Mean Δ YVMA	SD Δ YVMA	Mean Γ	Median Γ	Γ SD	Γ 2.5%	Γ 97.5%	Dominant direction	Direction retention
**9c→11f**	**hinge_MetN_to_ligand_acceptor_A**	1000	-0.005543	0.001903	-0.003864	0.001728	0.001679	0.001701	0.002548	-0.003509	0.006579	increase	0.753000

Δ = median(test) − median(base); Γ = ΔYVMA − ΔWT. Each protein uses min(Nbase, Ntest, 1000) structures sampled without replacement over 1,000 iterations (seed 20260916). SD denotes sample standard deviation. Γ 2.5% and Γ 97.5% follow the original sorted-index percentile method. Corrected N37 hinge descriptors are used for 10e and 11f.

Table S10. OC2 descriptor-space domain assessment and reference sensitivity analysis of OC2-derived conclusions.

Section A reports nearest-centroid descriptor-space distances, actual OOD95/OOD99 counts and fractions for all 14 Equal-N conditions, and the sensitivity of six primary matched-pair responses to excluding OOD99 structures. Section B reports reference-selection evidence. Section C reports reference sensitivity for the same six primary pairs.

Protein	Compound	Condition	Median nearest-centroid distance	OOD95 threshold	OOD99 threshold	Domain assignment	N/source status	N / >OOD95 n (%) / >OOD99 n (%)
WT	3	WT-3	1.3294	1.9723	2.8339	in-domain by OOD99 fraction	nearest-centroid descriptor-distance QC; N=1000 covalent structures	N=1000; >OOD95: 77 (7.7%); >OOD99: 1 (0.1%)
WT	4a	WT-4a	1.244	1.9723	2.8339	in-domain by OOD99 fraction	nearest-centroid descriptor-distance QC; N=1000 covalent structures	N=1000; >OOD95: 71 (7.1%); >OOD99: 1 (0.1%)
WT	5a	WT-5a	1.2496	1.9723	2.8339	in-domain by OOD99 fraction	nearest-centroid descriptor-distance QC; N=1000 covalent structures	N=1000; >OOD95: 70 (7.0%); >OOD99: 1 (0.1%)
WT	8c	WT-8c	1.267	1.9723	2.8339	in-domain by OOD99 fraction	nearest-centroid descriptor-distance QC; N=1000 covalent structures	N=1000; >OOD95: 73 (7.3%); >OOD99: 4 (0.4%)
WT	9c	WT-9c	1.3022	1.9723	2.8339	in-domain by OOD99 fraction	nearest-centroid descriptor-distance QC; N=1000 covalent structures	N=1000; >OOD95: 82 (8.2%); >OOD99: 2 (0.2%)
WT	10e	WT-10e	1.2877	1.9723	2.8339	in-domain by OOD99 fraction	nearest-centroid descriptor-distance QC; N=1000 covalent structures	N=1000; >OOD95: 66 (6.6%); >OOD99: 0 (0.0%)
WT	11f	WT-11f	1.3109	1.9723	2.8339	in-domain by OOD99 fraction	nearest-centroid descriptor-distance QC; N=1000 covalent structures	N=1000; >OOD95: 72 (7.2%); >OOD99: 3 (0.3%)
YVMA	3	YVMA-3	1.2539	1.9723	2.8339	in-domain by OOD99 fraction	nearest-centroid descriptor-distance QC; N=1000 covalent structures	N=1000; >OOD95: 113 (11.3%); >OOD99: 8 (0.8%)
YVMA	4a	YVMA-4a	1.3132	1.9723	2.8339	in-domain by OOD99 fraction	nearest-centroid descriptor-distance QC; N=1000 covalent structures	N=1000; >OOD95: 138 (13.8%); >OOD99: 6 (0.6%)
YVMA	5a	YVMA-5a	1.2836	1.9723	2.8339	in-domain by OOD99 fraction	nearest-centroid descriptor-distance QC; N=1000 covalent structures	N=1000; >OOD95: 120 (12.0%); >OOD99: 11 (1.1%)
YVMA	8c	YVMA-8c	1.257	1.9723	2.8339	in-domain by OOD99 fraction	nearest-centroid descriptor-distance QC; N=1000 covalent structures	N=1000; >OOD95: 120 (12.0%); >OOD99: 6 (0.6%)
YVMA	9c	YVMA-9c	1.2683	1.9723	2.8339	in-domain by OOD99 fraction	nearest-centroid descriptor-distance QC; N=1000 covalent structures	N=1000; >OOD95: 109 (10.9%); >OOD99: 6 (0.6%)
YVMA	10e	YVMA-10e	1.2446	1.9723	2.8339	in-domain by OOD99 fraction	nearest-centroid descriptor-distance QC; N=1000 covalent structures	N=1000; >OOD95: 91 (9.1%); >OOD99: 8 (0.8%)
YVMA	11f	YVMA-11f	1.2976	1.9723	2.8339	in-domain by OOD99 fraction	nearest-centroid descriptor-distance QC; N=1000 covalent structures	N=1000; >OOD95: 128 (12.8%); >OOD99: 12 (1.2%)

All 14 conditions have OOD99 fractions below 5% (observed range 0.0–1.2%; N=1000 per condition before exclusion). After removing OOD99 structures, the retained original k=5 assignments were renormalized within each condition, without retraining or reassigning clusters. All 29 originally nonzero state-level Γ

values retained their signs; the original zero Γ for 3→9c/S4 became −0.109 percentage points. The largest absolute change in Γ was 0.619 percentage points. These checks address the influence of descriptor-space outliers on reported sampled representations; they do not certify physical-state identity.

Primary pair	Original Γ S1–S5 (pp)	OOD99-excluded Γ S1–S5 (pp)	Max |ΔΓ| (pp)	Same sign incl. zero	Interpretation
3→4a	-9.000; +4.700; +1.100; +2.100; +1.100	-9.270; +4.589; +1.012; +2.004; +1.665	0.565	5/5	All nonzero signs retained
3→8c	-8.600; +2.900; +1.700; +2.100; +1.900	-8.926; +2.858; +1.681; +2.081; +2.306	0.406	5/5	All nonzero signs retained
3→9c	-3.600; +1.600; +0.800; -0.000; +1.200	-3.606; +1.561; +0.781; -0.109; +1.373	0.173	4/5	One zero became negative
4a→5a	+5.200; -3.500; +0.700; +0.700; -3.100	+5.463; -3.284; +0.771; +0.505; -3.455	0.355	5/5	All nonzero signs retained
8c→10e	+0.800; +1.000; -1.600; +0.500; -0.700	+1.102; +1.080; -1.567; +0.433; -1.048	0.348	5/5	All nonzero signs retained
9c→11f	+0.100; +0.300; -1.100; +1.400; -0.700	+0.243; +0.445; -1.077; +1.708; -1.319	0.619	5/5	All nonzero signs retained

Section B. Reference-selection evidence

Structure	Core RMSD to WT mean (Å)	Original-selection rank	Fixed-frame core medoid rank	Functional-region medoid rank	Role
7JXH_298_0.pdb	0.9272	1/918	1/918	223/918	Reference A
7JXH_261_2.pdb	1.4125	396/918	411/918	339/918	Alternative reference

Section C. Reference sensitivity analysis of OC2-derived conclusions

Analysis claim	Descriptor	7JXH_298_0	7JXH_261_2	Sensitivity	Final interpretation
APO WT/YVMA	DFG RMSD (Å)	WT 1.5087; YVMA 1.0951	WT 1.5497; YVMA 1.3562	Paired median |Δ|: WT 0.533; YVMA 0.449 Å	Reference-relative; use with independent geometry
APO WT/YVMA	αC RMSD (Å)	WT 2.8725; YVMA 3.2781	WT 3.7629; YVMA 5.1796	Paired median |Δ|: WT 2.040; YVMA 2.063 Å	Reference-sensitive; not a direct displacement
APO WT/YVMA	Lys51–Glu68 (Å)	WT 4.3987; YVMA 3.2737	WT 4.3987; YVMA 3.2737	No change	Reference-invariant internal distance; retained
APO WT/YVMA	P-loop RMSD (Å)	WT 3.3401; YVMA 3.8351	WT 2.4265; YVMA 3.0568	Paired median |Δ|: WT 1.248; YVMA 1.006 Å	Reference-sensitive; use with independent geometry
3→4a	Primary; Γ(S1–S5), N=1000	-0.090, +0.047, +0.011, +0.021, +0.011	-0.079, -0.089, +0.097, +0.003, +0.068	max |ΔΓ| 13.6 pp; same sign 4/5	Reference-sensitive descriptive support; state-specific Γ not primary
3→8c	Primary; Γ(S1–S5), N=1000	-0.086, +0.029, +0.017, +0.021, +0.019	-0.045, -0.035, -0.022, +0.034, +0.068	max |ΔΓ| 6.4 pp; same sign 3/5	Reference-sensitive descriptive support; state-specific Γ not primary
3→9c	Primary; Γ(S1–S5), N=1000	-0.036, +0.016, +0.008, -0.000, +0.012	+0.014, +0.000, -0.056, +0.039, +0.003	max |ΔΓ| 6.4 pp; same sign 1/5	Reference-sensitive descriptive support; state-specific Γ not primary
4a→5a	Primary; Γ(S1–S5), N=1000	+0.052, -0.035, +0.007, +0.007, -0.031	+0.012, +0.020, -0.031, +0.007, -0.008	max |ΔΓ| 5.5 pp; same sign 3/5	Reference-sensitive descriptive support; state-specific Γ not primary
8c→10e	Primary; Γ(S1–S5), N=1000	+0.008, +0.010, -0.016, +0.005, -0.007	+0.045, +0.022, -0.038, -0.004, -0.025	max |ΔΓ| 3.7 pp; same sign 4/5	Reference-sensitive descriptive support; state-specific Γ not primary
9c→11f	Primary; Γ(S1–S5), N=1000	+0.001, +0.003, -0.011, +0.014, -0.007	+0.006, +0.006, -0.031, -0.004, +0.023	max |ΔΓ| 3.0 pp; same sign 3/5	Reference-sensitive descriptive support; state-specific Γ not primary

Reference A was selected automatically as the WT APO structure closest to the aligned mean stable core. The controlled sensitivity analysis used the same 1,797 APO structures, four descriptors, 212 stable-core Cα atoms, k=5, random seed 42 and 30 initializations. Reference A values were reproduced with a maximum absolute error of 2.4 × 10^{-14} Å. After APO-overlap cluster matching, 866 of 1,797 assignments changed (48.19%; ARI=0.1978).

Table S11. Condition-level covalent-adduct MD summary across three independent replicas.

All condition-level metrics use the common 0–200 ns analysis window. Replica-level values are retained to make between-replica variation explicit.

Protein	Compound	Metric	n replicas	Replica values	Median	Q1	Q3	Mean	SD
WT	10e	C5_shell_min_distance_A	3	3.8100;3.7602;3.7738	3.7738	3.767	3.7919	3.7813	0.0257
WT	10e	DFG_ca_rmsd_A	3	1.9120;2.1728;2.1000	2.1	2.006	2.1364	2.0616	0.1346
WT	10e	Lys51_Glu68_min_distance_A	3	9.1611;10.1935;10.8966	10.194	9.6773	10.545	10.084	0.8729
WT	10e	alphaC_ca_rmsd_A	3	2.2532;2.1979;1.7764	2.1979	1.9872	2.2256	2.0758	0.2608
WT	10e	hinge_distance_A	3	3.0618;3.0255;3.0827	3.0618	3.0436	3.0722	3.0567	0.0289
WT	10e	ligand_heavy_rmsd_A	3	1.4785;1.8408;1.6839	1.6839	1.5812	1.7624	1.6677	0.1817
WT	10e	p_loop_ca_rmsd_A	3	2.5903;3.2971;2.4496	2.5903	2.52	2.9437	2.779	0.4542
WT	10e	protein_ca_rmsd_A	3	3.5810;3.4170;3.1659	3.417	3.2915	3.499	3.388	0.2091
WT	11f	C5_shell_min_distance_A	3	3.6224;3.6234;3.6251	3.6234	3.6229	3.6242	3.6236	0.0014
WT	11f	DFG_ca_rmsd_A	3	1.3273;2.1358;1.9680	1.968	1.6477	2.0519	1.8104	0.4267
WT	11f	Lys51_Glu68_min_distance_A	3	13.7594;11.3646;11.3512	11.365	11.358	12.562	12.158	1.3865
WT	11f	alphaC_ca_rmsd_A	3	1.5772;2.3648;4.9524	2.3648	1.971	3.6586	2.9648	1.7658
WT	11f	hinge_distance_A	3	2.9857;2.9977;3.0159	2.9977	2.9917	3.0068	2.9998	0.0152
WT	11f	ligand_heavy_rmsd_A	3	2.0312;2.1389;2.2884	2.1389	2.085	2.2137	2.1528	0.1292
WT	11f	p_loop_ca_rmsd_A	3	2.6284;2.3993;3.4736	2.6284	2.5139	3.051	2.8338	0.5658
WT	11f	protein_ca_rmsd_A	3	2.3184;3.2979;3.6373	3.2979	2.8081	3.4676	3.0845	0.6848
WT	3	C5_shell_min_distance_A	3	3.9926;4.0373;4.0325	4.0325	4.0126	4.0349	4.0208	0.0245
WT	3	DFG_ca_rmsd_A	3	1.2984;1.4123;1.8702	1.4123	1.3554	1.6413	1.527	0.3027
WT	3	Lys51_Glu68_min_distance_A	3	9.7132;13.9628;13.0466	13.047	11.38	13.505	12.241	2.2364
WT	3	alphaC_ca_rmsd_A	3	3.0819;4.4886;4.2626	4.2626	3.6723	4.3756	3.9444	0.7554
WT	3	hinge_distance_A	3	3.0458;3.0394;3.4839	3.0458	3.0426	3.2649	3.1897	0.2548
WT	3	ligand_heavy_rmsd_A	3	2.8016;2.9434;2.1583	2.8016	2.48	2.8725	2.6344	0.4184
WT	3	p_loop_ca_rmsd_A	3	3.0589;3.4155;3.0020	3.0589	3.0305	3.2372	3.1588	0.2241
WT	3	protein_ca_rmsd_A	3	3.1242;3.9284;3.3881	3.3881	3.2561	3.6582	3.4802	0.4099
WT	4a	C5_shell_min_distance_A	3	3.6081;3.5448;3.5526	3.5526	3.5487	3.5804	3.5685	0.0345
WT	4a	DFG_ca_rmsd_A	3	3.3910;2.0771;2.0766	2.0771	2.0769	2.734	2.5149	0.7587
WT	4a	Lys51_Glu68_min_distance_A	3	10.7517;10.6462;10.6821	10.682	10.664	10.717	10.693	0.0536
WT	4a	alphaC_ca_rmsd_A	3	2.9853;1.7334;1.7546	1.7546	1.744	2.37	2.1578	0.7167
WT	4a	hinge_distance_A	3	3.1220;3.0407;3.0443	3.0443	3.0425	3.0831	3.069	0.0459
WT	4a	ligand_heavy_rmsd_A	3	1.9760;2.7076;2.6923	2.6923	2.3342	2.6999	2.4586	0.418
WT	4a	p_loop_ca_rmsd_A	3	2.3235;2.9170;2.9417	2.917	2.6202	2.9293	2.7274	0.35
WT	4a	protein_ca_rmsd_A	3	3.0662;2.2813;2.2842	2.2842	2.2828	2.6752	2.5439	0.4523
WT	5a	C5_shell_min_distance_A	3	3.5372;3.5715;3.5740	3.5715	3.5543	3.5728	3.5609	0.0206
WT	5a	DFG_ca_rmsd_A	3	1.4526;2.1181;1.7136	1.7136	1.5831	1.9159	1.7614	0.3353
WT	5a	Lys51_Glu68_min_distance_A	3	12.9432;11.8310;11.7393	11.831	11.785	12.387	12.171	0.6702
WT	5a	alphaC_ca_rmsd_A	3	2.5190;4.7700;4.6688	4.6688	3.5939	4.7194	3.9859	1.2714
WT	5a	hinge_distance_A	3	3.0493;3.1481;3.1890	3.1481	3.0987	3.1685	3.1288	0.0718

WT	5a	ligand_heavy_rmsd_A	3	1.8569;2.3331;1.1934	1.8569	1.5252	2.095	1.7945	0.5724
WT	5a	p_loop_ca_rmsd_A	3	2.8373;3.4716;2.1757	2.8373	2.5065	3.1544	2.8282	0.648
WT	5a	protein_ca_rmsd_A	3	3.2927;3.7930;3.2433	3.2927	3.268	3.5429	3.443	0.3041
WT	8c	C5_shell_min_distance_A	3	3.8764;4.0365;3.7944	3.8764	3.8354	3.9565	3.9024	0.1231
WT	8c	DFG_ca_rmsd_A	3	1.4400;1.3988;0.8474	1.3988	1.1231	1.4194	1.2287	0.3309
WT	8c	Lys51_Glu68_min_distance_A	3	12.4693;12.0941;14.1275	12.469	12.282	13.298	12.897	1.0821
WT	8c	alphaC_ca_rmsd_A	3	3.1509;3.8125;4.0799	3.8125	3.4817	3.9462	3.6811	0.4782
WT	8c	hinge_distance_A	3	3.0742;3.0737;3.0377	3.0737	3.0557	3.0739	3.0619	0.0209
WT	8c	ligand_heavy_rmsd_A	3	2.4646;1.7096;1.3996	1.7096	1.5546	2.0871	1.8579	0.5478
WT	8c	p_loop_ca_rmsd_A	3	3.4773;2.8360;2.7083	2.836	2.7721	3.1566	3.0072	0.4121
WT	8c	protein_ca_rmsd_A	3	3.6506;3.7711;2.9053	3.6506	3.2779	3.7108	3.4423	0.469
WT	9c	C5_shell_min_distance_A	3	3.8650;3.9119;3.6830	3.865	3.774	3.8885	3.82	0.1209
WT	9c	DFG_ca_rmsd_A	3	2.8874;1.8226;1.0576	1.8226	1.4401	2.355	1.9225	0.919
WT	9c	Lys51_Glu68_min_distance_A	3	13.8628;11.0349;12.8282	12.828	11.932	13.345	12.575	1.4308
WT	9c	alphaC_ca_rmsd_A	3	2.3988;3.8804;2.0479	2.3988	2.2233	3.1396	2.7757	0.9727
WT	9c	hinge_distance_A	3	2.9880;3.0610;2.9838	2.988	2.9859	3.0245	3.0109	0.0434
WT	9c	ligand_heavy_rmsd_A	3	1.8411;1.8134;2.0419	1.8411	1.8272	1.9415	1.8988	0.1247
WT	9c	p_loop_ca_rmsd_A	3	3.3658;2.7718;3.0804	3.0804	2.9261	3.2231	3.0727	0.2971
WT	9c	protein_ca_rmsd_A	3	2.6600;3.0960;3.4295	3.096	2.878	3.2628	3.0618	0.3859
YVMA	10e	C5_shell_min_distance_A	3	3.7581;3.7404;3.7875	3.7581	3.7492	3.7728	3.762	0.0238
YVMA	10e	DFG_ca_rmsd_A	3	0.8016;2.2641;0.7122	0.8016	0.7569	1.5329	1.2593	0.8713
YVMA	10e	Lys51_Glu68_min_distance_A	3	20.8624;22.7816;19.3084	20.862	20.085	21.822	20.984	1.7398
YVMA	10e	alphaC_ca_rmsd_A	3	7.2766;5.8329;5.2140	5.8329	5.5235	6.5548	6.1078	1.0584
YVMA	10e	hinge_distance_A	3	3.0933;3.0723;3.0759	3.0759	3.0741	3.0846	3.0805	0.0112
YVMA	10e	ligand_heavy_rmsd_A	3	1.1643;1.5181;1.1625	1.1643	1.1634	1.3412	1.2816	0.2048
YVMA	10e	p_loop_ca_rmsd_A	3	2.7139;2.8389;2.4251	2.7139	2.5695	2.7764	2.6593	0.2122
YVMA	10e	protein_ca_rmsd_A	3	2.8024;2.7607;2.6932	2.7607	2.7269	2.7816	2.7521	0.0551
YVMA	11f	C5_shell_min_distance_A	3	3.6379;3.7515;3.8196	3.7515	3.6947	3.7855	3.7363	0.0918
YVMA	11f	DFG_ca_rmsd_A	3	0.7375;2.1618;1.7341	1.7341	1.2358	1.948	1.5445	0.7308
YVMA	11f	Lys51_Glu68_min_distance_A	3	22.4055;21.6461;22.0695	22.07	21.858	22.238	22.04	0.3805
YVMA	11f	alphaC_ca_rmsd_A	3	5.7513;5.2373;4.0690	5.2373	4.6532	5.4943	5.0192	0.8621
YVMA	11f	hinge_distance_A	3	3.0881;3.0730;3.0150	3.0730	3.0440	3.0805	3.0587	0.0386
YVMA	11f	ligand_heavy_rmsd_A	3	1.8193;2.0373;2.6672	2.0373	1.9283	2.3522	2.1746	0.4403
YVMA	11f	p_loop_ca_rmsd_A	3	2.1568;3.2020;3.4576	3.202	2.6794	3.3298	2.9388	0.6892
YVMA	11f	protein_ca_rmsd_A	3	2.5843;2.7224;2.5415	2.5843	2.5629	2.6533	2.6161	0.0945
YVMA	3	C5_shell_min_distance_A	3	3.9216;4.0756;4.0623	4.0623	3.992	4.0689	4.0198	0.0853
YVMA	3	DFG_ca_rmsd_A	3	1.5921;1.4361;2.1322	1.5921	1.5141	1.8622	1.7201	0.3653
YVMA	3	Lys51_Glu68_min_distance_A	3	22.5356;22.5909;21.0716	22.536	21.804	22.563	22.066	0.8616
YVMA	3	alphaC_ca_rmsd_A	3	6.3912;4.1232;4.4345	4.4345	4.2789	5.4129	4.983	1.2295
YVMA	3	hinge_distance_A	3	3.0713;3.1501;3.1243	3.1243	3.0978	3.1372	3.1152	0.0402
YVMA	3	ligand_heavy_rmsd_A	3	1.9998;2.9296;1.6685	1.9998	1.8342	2.4647	2.1993	0.6538

YVMA	3	p_loop_ca_rmsd_A	3	2.4141;5.4511;3.3002	3.3002	2.8571	4.3757	3.7218	1.5618
YVMA	3	protein_ca_rmsd_A	3	2.7999;3.3914;2.3320	2.7999	2.5659	3.0957	2.8411	0.5309
YVMA	4a	C5_shell_min_distance_A	3	3.5114;3.3861;3.4071	3.4071	3.3966	3.4592	3.4349	0.0671
YVMA	4a	DFG_ca_rmsd_A	3	2.1608;2.3596;1.6321	2.1608	1.8965	2.2602	2.0508	0.376
YVMA	4a	Lys51_Glu68_min_distance_A	3	21.4885;22.2420;23.4474	22.242	21.865	22.845	22.393	0.9881
YVMA	4a	alphaC_ca_rmsd_A	3	4.2542;6.4916;4.8421	4.8421	4.5481	5.6669	5.196	1.1599
YVMA	4a	hinge_distance_A	3	3.1665;3.2295;3.2564	3.2295	3.198	3.2429	3.2175	0.0461
YVMA	4a	ligand_heavy_rmsd_A	3	1.4915;1.2571;1.3928	1.3928	1.325	1.4422	1.3805	0.1177
YVMA	4a	p_loop_ca_rmsd_A	3	3.0484;1.1729;1.8181	1.8181	1.4955	2.4333	2.0131	0.9528
YVMA	4a	protein_ca_rmsd_A	3	2.5472;2.3239;2.7286	2.5472	2.4356	2.6379	2.5332	0.2027
YVMA	5a	C5_shell_min_distance_A	3	3.4455;3.4284;3.4583	3.4455	3.4369	3.4519	3.4441	0.015
YVMA	5a	DFG_ca_rmsd_A	3	2.5887;0.8928;1.2247	1.2247	1.0587	1.9067	1.5687	0.8988
YVMA	5a	Lys51_Glu68_min_distance_A	3	23.7762;20.0558;21.8501	21.85	20.953	22.813	21.894	1.8606
YVMA	5a	alphaC_ca_rmsd_A	3	5.4127;3.9742;3.8992	3.9742	3.9367	4.6935	4.4287	0.853
YVMA	5a	hinge_distance_A	3	3.2517;3.1926;3.3760	3.2517	3.2222	3.3138	3.2734	0.0936
YVMA	5a	ligand_heavy_rmsd_A	3	1.5524;1.5211;1.4161	1.5211	1.4686	1.5368	1.4965	0.0714
YVMA	5a	p_loop_ca_rmsd_A	3	2.5013;2.3616;2.4864	2.4864	2.424	2.4939	2.4498	0.0767
YVMA	5a	protein_ca_rmsd_A	3	2.8240;2.2847;2.7073	2.7073	2.496	2.7656	2.6053	0.2837
YVMA	8c	C5_shell_min_distance_A	3	3.8994;3.9640;4.0378	3.964	3.9317	4.0009	3.9671	0.0693
YVMA	8c	DFG_ca_rmsd_A	3	2.2911;2.2457;2.0385	2.2457	2.1421	2.2684	2.1918	0.1347
YVMA	8c	Lys51_Glu68_min_distance_A	3	22.6057;21.1699;23.6127	22.606	21.888	23.109	22.463	1.2277
YVMA	8c	alphaC_ca_rmsd_A	3	4.7817;6.0897;6.5335	6.0897	5.4357	6.3116	5.8016	0.9107
YVMA	8c	hinge_distance_A	3	3.2321;3.1292;3.1317	3.1317	3.1304	3.1819	3.1643	0.0587
YVMA	8c	ligand_heavy_rmsd_A	3	1.2550;1.4729;1.4350	1.435	1.345	1.454	1.3876	0.1164
YVMA	8c	p_loop_ca_rmsd_A	3	2.4082;2.3544;2.3179	2.3544	2.3361	2.3813	2.3602	0.0454
YVMA	8c	protein_ca_rmsd_A	3	2.2913;2.8899;2.5461	2.5461	2.4187	2.718	2.5758	0.3004
YVMA	9c	C5_shell_min_distance_A	3	3.8505;3.8026;3.7729	3.8026	3.7877	3.8266	3.8087	0.0392
YVMA	9c	DFG_ca_rmsd_A	3	2.2439;2.2991;2.4806	2.2991	2.2715	2.3899	2.3412	0.1238
YVMA	9c	Lys51_Glu68_min_distance_A	3	22.7386;22.7662;23.2137	22.766	22.752	22.99	22.906	0.2667
YVMA	9c	alphaC_ca_rmsd_A	3	4.5614;3.5896;5.8714	4.5614	4.0755	5.2164	4.6741	1.1451
YVMA	9c	hinge_distance_A	3	3.1141;3.0747;3.0701	3.0747	3.0724	3.0944	3.0863	0.0242
YVMA	9c	ligand_heavy_rmsd_A	3	1.9684;2.0169;2.2062	2.0169	1.9927	2.1116	2.0638	0.1257
YVMA	9c	p_loop_ca_rmsd_A	3	2.7925;3.2060;2.5107	2.7925	2.6516	2.9992	2.8364	0.3497
YVMA	9c	protein_ca_rmsd_A	3	2.5885;2.4367;2.7264	2.5885	2.5126	2.6574	2.5839	0.1449

Table S12. Matched-perturbation changes in covalent-adduct MD readouts.

Δ = mean(B) − mean(A) for each A→B perturbation. The table includes the six primary perturbations.

Role	Protein	Transition	Metric	Base replica values	Test replica values	Base mean	Test mean	Δ (B−A)	Replica variability	Interpretation flag
primary	WT	3→4a	C5_shell_min_distance_A	3.9926;4.0373;4.0325	3.6081;3.5448;3.5526	4.0208	3.5685	-0.4523	0.0345	clear_difference
primary	WT	3→4a	DFG_ca_rmsd_A	1.2984;1.4123;1.8702	3.3910;2.0771;2.0766	1.527	2.5149	0.9879	0.7587	clear_difference
primary	WT	3→4a	Lys51_Glu68_min_distance_A	9.7132;13.9628;13.0466	10.7517;10.6462;10.6821	12.241	10.693	-1.5475	2.2364	modest_difference
primary	WT	3→4a	alphaC_ca_rmsd_A	3.0819;4.4886;4.2626	2.9853;1.7334;1.7546	3.9444	2.1578	-1.7866	0.7554	clear_difference
primary	WT	3→4a	hinge_distance_A	3.0458;3.0394;3.4839	3.1220;3.0407;3.0443	3.1897	3.069	-0.1207	0.2548	modest_difference
primary	WT	3→4a	ligand_heavy_rmsd_A	2.8016;2.9434;2.1583	1.9760;2.7076;2.6923	2.6344	2.4586	-0.1758	0.4184	similar
primary	WT	3→4a	p_loop_ca_rmsd_A	3.0589;3.4155;3.0020	2.3235;2.9170;2.9417	3.1588	2.7274	-0.4314	0.35	clear_difference
primary	WT	3→4a	protein_ca_rmsd_A	3.1242;3.9284;3.3881	3.0662;2.2813;2.2842	3.4802	2.5439	-0.9363	0.4523	clear_difference
primary	WT	3→8c	C5_shell_min_distance_A	3.9926;4.0373;4.0325	3.8764;4.0365;3.7944	4.0208	3.9024	-0.1184	0.1231	high_replica_variability
primary	WT	3→8c	DFG_ca_rmsd_A	1.2984;1.4123;1.8702	1.4400;1.3988;0.8474	1.527	1.2287	-0.2982	0.3309	modest_difference
primary	WT	3→8c	Lys51_Glu68_min_distance_A	9.7132;13.9628;13.0466	12.4693;12.0941;14.1275	12.241	12.897	0.6561	2.2364	similar
primary	WT	3→8c	alphaC_ca_rmsd_A	3.0819;4.4886;4.2626	3.1509;3.8125;4.0799	3.9444	3.6811	-0.2633	0.7554	similar
primary	WT	3→8c	hinge_distance_A	3.0458;3.0394;3.4839	3.0742;3.0737;3.0377	3.1897	3.0619	-0.1278	0.2548	modest_difference
primary	WT	3→8c	ligand_heavy_rmsd_A	2.8016;2.9434;2.1583	2.4646;1.7096;1.3996	2.6344	1.8579	-0.7765	0.5478	clear_difference
primary	WT	3→8c	p_loop_ca_rmsd_A	3.0589;3.4155;3.0020	3.4773;2.8360;2.7083	3.1588	3.0072	-0.1516	0.4121	similar
primary	WT	3→8c	protein_ca_rmsd_A	3.1242;3.9284;3.3881	3.6506;3.7711;2.9053	3.4802	3.4423	-0.0379	0.469	similar
primary	WT	3→9c	C5_shell_min_distance_A	3.9926;4.0373;4.0325	3.8650;3.9119;3.6830	4.0208	3.82	-0.2008	0.1209	clear_difference
primary	WT	3→9c	DFG_ca_rmsd_A	1.2984;1.4123;1.8702	2.8874;1.8226;1.0576	1.527	1.9225	0.3956	0.919	modest_difference
primary	WT	3→9c	Lys51_Glu68_min_distance_A	9.7132;13.9628;13.0466	13.8628;11.0349;12.8282	12.241	12.575	0.3344	2.2364	similar
primary	WT	3→9c	alphaC_ca_rmsd_A	3.0819;4.4886;4.2626	2.3988;3.8804;2.0479	3.9444	2.7757	-1.1687	0.9727	clear_difference
primary	WT	3→9c	hinge_distance_A	3.0458;3.0394;3.4839	2.9880;3.0610;2.9838	3.1897	3.0109	-0.1788	0.2548	modest_difference
primary	WT	3→9c	ligand_heavy_rmsd_A	2.8016;2.9434;2.1583	1.8411;1.8134;2.0419	2.6344	1.8988	-0.7356	0.4184	clear_difference
primary	WT	3→9c	p_loop_ca_rmsd_A	3.0589;3.4155;3.0020	3.3658;2.7718;3.0804	3.1588	3.0727	-0.0861	0.2971	similar
primary	WT	3→9c	protein_ca_rmsd_A	3.1242;3.9284;3.3881	2.6600;3.0960;3.4295	3.4802	3.0618	-0.4184	0.4099	modest_difference
primary	WT	4a→5a	C5_shell_min_distance_A	3.6081;3.5448;3.5526	3.5372;3.5715;3.5740	3.5685	3.5609	-0.0076	0.0345	similar
primary	WT	4a→5a	DFG_ca_rmsd_A	3.3910;2.0771;2.0766	1.4526;2.1181;1.7136	2.5149	1.7614	-0.7535	0.7587	high_replica_variability
primary	WT	4a→5a	Lys51_Glu68_min_distance_A	10.7517;10.6462;10.6821	12.9432;11.8310;11.7393	10.693	12.171	1.4778	0.6702	clear_difference
primary	WT	4a→5a	alphaC_ca_rmsd_A	2.9853;1.7334;1.7546	2.5190;4.7700;4.6688	2.1578	3.9859	1.8282	1.2714	clear_difference
primary	WT	4a→5a	hinge_distance_A	3.1220;3.0407;3.0443	3.0493;3.1481;3.1890	3.069	3.1288	0.0598	0.0718	modest_difference
primary	WT	4a→5a	ligand_heavy_rmsd_A	1.9760;2.7076;2.6923	1.8569;2.3331;1.1934	2.4586	1.7945	-0.6642	0.5724	clear_difference
primary	WT	4a→5a	p_loop_ca_rmsd_A	2.3235;2.9170;2.9417	2.8373;3.4716;2.1757	2.7274	2.8282	0.1008	0.648	similar
primary	WT	4a→5a	protein_ca_rmsd_A	3.0662;2.2813;2.2842	3.2927;3.7930;3.2433	2.5439	3.443	0.8991	0.4523	clear_difference
primary	WT	8c→10e	C5_shell_min_distance_A	3.8764;4.0365;3.7944	3.8100;3.7602;3.7738	3.9024	3.7813	-0.1211	0.1231	high_replica_variability
primary	WT	8c→10e	DFG_ca_rmsd_A	1.4400;1.3988;0.8474	1.9120;2.1728;2.1000	1.2287	2.0616	0.8329	0.3309	clear_difference
primary	WT	8c→10e	Lys51_Glu68_min_distance_A	12.4693;12.0941;14.1275	9.1611;10.1935;10.8966	12.897	10.084	-2.8132	1.0821	clear_difference
primary	WT	8c→10e	alphaC_ca_rmsd_A	3.1509;3.8125;4.0799	2.2532;2.1979;1.7764	3.6811	2.0758	-1.6053	0.4782	clear_difference

primary	WT	8c→10e	hinge_distance_A	3.0742;3.0737;3.0377	3.0618;3.0255;3.0827	3.0619	3.0567	-0.0052	0.0289	similar
primary	WT	8c→10e	ligand_heavy_rmsd_A	2.4646;1.7096;1.3996	1.4785;1.8408;1.6839	1.8579	1.6677	-0.1902	0.5478	modest_difference
primary	WT	8c→10e	p_loop_ca_rmsd_A	3.4773;2.8360;2.7083	2.5903;3.2971;2.4496	3.0072	2.779	-0.2282	0.4542	modest_difference
primary	WT	8c→10e	protein_ca_rmsd_A	3.6506;3.7711;2.9053	3.5810;3.4170;3.1659	3.4423	3.388	-0.0544	0.469	similar
primary	WT	9c→11f	C5_shell_min_distance_A	3.8650;3.9119;3.6830	3.6224;3.6234;3.6251	3.82	3.6236	-0.1963	0.1209	clear_difference
primary	WT	9c→11f	DFG_ca_rmsd_A	2.8874;1.8226;1.0576	1.3273;2.1358;1.9680	1.9225	1.8104	-0.1122	0.919	similar
primary	WT	9c→11f	Lys51_Glu68_min_distance_A	13.8628;11.0349;12.8282	13.7594;11.3646;11.3512	12.575	12.158	-0.4169	1.4308	similar
primary	WT	9c→11f	alphaC_ca_rmsd_A	2.3988;3.8804;2.0479	1.5772;2.3648;4.9524	2.7757	2.9648	0.1891	1.7658	similar
primary	WT	9c→11f	hinge_distance_A	2.9880;3.0610;2.9838	2.9857;2.9977;3.0159	3.0109	2.9998	-0.0112	0.0434	similar
primary	WT	9c→11f	ligand_heavy_rmsd_A	1.8411;1.8134;2.0419	2.0312;2.1389;2.2884	1.8988	2.1528	0.2540	0.1292	clear_difference
primary	WT	9c→11f	p_loop_ca_rmsd_A	3.3658;2.7718;3.0804	2.6284;2.3993;3.4736	3.0727	2.8338	-0.2389	0.5658	modest_difference
primary	WT	9c→11f	protein_ca_rmsd_A	2.6600;3.0960;3.4295	2.3184;3.2979;3.6373	3.0618	3.0845	0.0227	0.6848	similar
primary	YVMA	3→4a	C5_shell_min_distance_A	3.9216;4.0756;4.0623	3.5114;3.3861;3.4071	4.0198	3.4349	-0.585	0.0853	clear_difference
primary	YVMA	3→4a	DFG_ca_rmsd_A	1.5921;1.4361;2.1322	2.1608;2.3596;1.6321	1.7201	2.0508	0.3307	0.376	modest_difference
primary	YVMA	3→4a	Lys51_Glu68_min_distance_A	22.5356;22.5909;21.0716	21.4885;22.2420;23.4474	22.066	22.393	0.3266	0.9881	similar
primary	YVMA	3→4a	alphaC_ca_rmsd_A	6.3912;4.1232;4.4345	4.2542;6.4916;4.8421	4.983	5.196	0.213	1.2295	similar
primary	YVMA	3→4a	hinge_distance_A	3.0713;3.1501;3.1243	3.1665;3.2295;3.2564	3.1152	3.2175	0.1022	0.0461	clear_difference
primary	YVMA	3→4a	ligand_heavy_rmsd_A	1.9998;2.9296;1.6685	1.4915;1.2571;1.3928	2.1993	1.3805	-0.8188	0.6538	clear_difference
primary	YVMA	3→4a	p_loop_ca_rmsd_A	2.4141;5.4511;3.3002	3.0484;1.1729;1.8181	3.7218	2.0131	-1.7087	1.5618	clear_difference
primary	YVMA	3→4a	protein_ca_rmsd_A	2.7999;3.3914;2.3320	2.5472;2.3239;2.7286	2.8411	2.5332	-0.3079	0.5309	modest_difference
primary	YVMA	3→8c	C5_shell_min_distance_A	3.9216;4.0756;4.0623	3.8994;3.9640;4.0378	4.0198	3.9671	-0.0528	0.0853	modest_difference
primary	YVMA	3→8c	DFG_ca_rmsd_A	1.5921;1.4361;2.1322	2.2911;2.2457;2.0385	1.7201	2.1918	0.4716	0.3653	clear_difference
primary	YVMA	3→8c	Lys51_Glu68_min_distance_A	22.5356;22.5909;21.0716	22.6057;21.1699;23.6127	22.066	22.463	0.3967	1.2277	similar
primary	YVMA	3→8c	alphaC_ca_rmsd_A	6.3912;4.1232;4.4345	4.7817;6.0897;6.5335	4.983	5.8016	0.8187	1.2295	modest_difference
primary	YVMA	3→8c	hinge_distance_A	3.0713;3.1501;3.1243	3.2321;3.1292;3.1317	3.1152	3.1643	0.0491	0.0587	modest_difference
primary	YVMA	3→8c	ligand_heavy_rmsd_A	1.9998;2.9296;1.6685	1.2550;1.4729;1.4350	2.1993	1.3876	-0.8117	0.6538	clear_difference
primary	YVMA	3→8c	p_loop_ca_rmsd_A	2.4141;5.4511;3.3002	2.4082;2.3544;2.3179	3.7218	2.3602	-1.3616	1.5618	high_replica_variability
primary	YVMA	3→8c	protein_ca_rmsd_A	2.7999;3.3914;2.3320	2.2913;2.8899;2.5461	2.8411	2.5758	-0.2653	0.5309	modest_difference
primary	YVMA	3→9c	C5_shell_min_distance_A	3.9216;4.0756;4.0623	3.8505;3.8026;3.7729	4.0198	3.8087	-0.2112	0.0853	clear_difference
primary	YVMA	3→9c	DFG_ca_rmsd_A	1.5921;1.4361;2.1322	2.2439;2.2991;2.4806	1.7201	2.3412	0.6211	0.3653	clear_difference
primary	YVMA	3→9c	Lys51_Glu68_min_distance_A	22.5356;22.5909;21.0716	22.7386;22.7662;23.2137	22.066	22.906	0.8401	0.8616	high_replica_variability
primary	YVMA	3→9c	alphaC_ca_rmsd_A	6.3912;4.1232;4.4345	4.5614;3.5896;5.8714	4.983	4.6741	-0.3088	1.2295	similar
primary	YVMA	3→9c	hinge_distance_A	3.0713;3.1501;3.1243	3.1141;3.0747;3.0701	3.1152	3.0863	-0.0289	0.0402	modest_difference
primary	YVMA	3→9c	ligand_heavy_rmsd_A	1.9998;2.9296;1.6685	1.9684;2.0169;2.2062	2.1993	2.0638	-0.1355	0.6538	similar
primary	YVMA	3→9c	p_loop_ca_rmsd_A	2.4141;5.4511;3.3002	2.7925;3.2060;2.5107	3.7218	2.8364	-0.8854	1.5618	modest_difference
primary	YVMA	3→9c	protein_ca_rmsd_A	2.7999;3.3914;2.3320	2.5885;2.4367;2.7264	2.8411	2.5839	-0.2572	0.5309	modest_difference
primary	YVMA	4a→5a	C5_shell_min_distance_A	3.5114;3.3861;3.4071	3.4455;3.4284;3.4583	3.4349	3.4441	0.0092	0.0671	similar
primary	YVMA	4a→5a	DFG_ca_rmsd_A	2.1608;2.3596;1.6321	2.5887;0.8928;1.2247	2.0508	1.5687	-0.4821	0.8988	modest_difference
primary	YVMA	4a→5a	Lys51_Glu68_min_distance_A	21.4885;22.2420;23.4474	23.7762;20.0558;21.8501	22.393	21.894	-0.4986	1.8606	similar
primary	YVMA	4a→5a	alphaC_ca_rmsd_A	4.2542;6.4916;4.8421	5.4127;3.9742;3.8992	5.196	4.4287	-0.7673	1.1599	modest_difference
primary	YVMA	4a→5a	hinge_distance_A	3.1665;3.2295;3.2564	3.2517;3.1926;3.3760	3.2175	3.2734	0.056	0.0936	modest_difference

primary	YVMA	4a→5a	ligand_heavy_rmsd_A	1.4915;1.2571;1.3928	1.5524;1.5211;1.4161	1.3805	1.4965	0.1161	0.1177	high_replica_variability
primary	YVMA	4a→5a	p_loop_ca_rmsd_A	3.0484;1.1729;1.8181	2.5013;2.3616;2.4864	2.0131	2.4498	0.4366	0.9528	modest_difference
primary	YVMA	4a→5a	protein_ca_rmsd_A	2.5472;2.3239;2.7286	2.8240;2.2847;2.7073	2.5332	2.6053	0.0721	0.2837	similar
primary	YVMA	8c→10e	C5_shell_min_distance_A	3.8994;3.9640;4.0378	3.7581;3.7404;3.7875	3.9671	3.762	-0.2051	0.0693	clear_difference
primary	YVMA	8c→10e	DFG_ca_rmsd_A	2.2911;2.2457;2.0385	0.8016;2.2641;0.7122	2.1918	1.2593	-0.9325	0.8713	clear_difference
primary	YVMA	8c→10e	Lys51_Glu68_min_distance_A	22.6057;21.1699;23.6127	20.8624;22.7816;19.3084	22.463	20.984	-1.4786	1.7398	modest_difference
primary	YVMA	8c→10e	alphaC_ca_rmsd_A	4.7817;6.0897;6.5335	7.2766;5.8329;5.2140	5.8016	6.1078	0.3062	1.0584	similar
primary	YVMA	8c→10e	hinge_distance_A	3.2321;3.1292;3.1317	3.0933;3.0723;3.0759	3.1643	3.0805	-0.0838	0.0587	clear_difference
primary	YVMA	8c→10e	ligand_heavy_rmsd_A	1.2550;1.4729;1.4350	1.1643;1.5181;1.1625	1.3876	1.2816	-0.106	0.2048	modest_difference
primary	YVMA	8c→10e	p_loop_ca_rmsd_A	2.4082;2.3544;2.3179	2.7139;2.8389;2.4251	2.3602	2.6593	0.2991	0.2122	clear_difference
primary	YVMA	8c→10e	protein_ca_rmsd_A	2.2913;2.8899;2.5461	2.8024;2.7607;2.6932	2.5758	2.7521	0.1763	0.3004	modest_difference
primary	YVMA	9c→11f	C5_shell_min_distance_A	3.8505;3.8026;3.7729	3.6379;3.7515;3.8196	3.8087	3.7363	-0.0723	0.0918	modest_difference
primary	YVMA	9c→11f	DFG_ca_rmsd_A	2.2439;2.2991;2.4806	0.7375;2.1618;1.7341	2.3412	1.5445	-0.7967	0.7308	clear_difference
primary	YVMA	9c→11f	Lys51_Glu68_min_distance_A	22.7386;22.7662;23.2137	22.4055;21.6461;22.0695	22.906	22.04	-0.8658	0.3805	clear_difference
primary	YVMA	9c→11f	alphaC_ca_rmsd_A	4.5614;3.5896;5.8714	5.7513;5.2373;4.0690	4.6741	5.0192	0.3451	1.1451	similar
primary	YVMA	9c→11f	hinge_distance_A	3.1141;3.0747;3.0701	3.0881;3.0730;3.0150	3.0863	3.0587	-0.0276	0.0386	modest_difference
primary	YVMA	9c→11f	ligand_heavy_rmsd_A	1.9684;2.0169;2.2062	1.8193;2.0373;2.6672	2.0638	2.1746	0.1108	0.4403	similar
primary	YVMA	9c→11f	p_loop_ca_rmsd_A	2.7925;3.2060;2.5107	2.1568;3.2020;3.4576	2.8364	2.9388	0.1024	0.6892	similar
primary	YVMA	9c→11f	protein_ca_rmsd_A	2.5885;2.4367;2.7264	2.5843;2.7224;2.5415	2.5839	2.6161	0.0322	0.1449	similar

Table S13. Unified protein and ligand atom mapping used for NAC, hinge, C5-shell and covalent analyses.

Reactive cysteine numbering follows the structure-specific numbering: Cys805 in 7JXH (WT) and Cys809 in 8U8X (YVMA).

Protein	PDB	Compound	Lineage	Reactive Cys	S atom	Electrophilic C	Adjacent C	Hinge reference atom	C5-shell atom definition	Selection method	Mapping confidence	Used analyses
WT	7JXH	3		Cys805	SG	C5	C6	N17	compound-specific C5 moiety atom set	OC2 descriptor columns plus covalent QC mapping	confirmed	NAC/hinge/C5/tail
WT	7JXH	4a		Cys805	SG	C5	C6	N22	compound-specific C5 moiety atom set	OC2 descriptor columns plus covalent QC mapping	confirmed	NAC/hinge/C5/tail
WT	7JXH	5a	4a->5a	Cys805	SG	C31	C30	N41	compound-specific C5 moiety atom set	OC2 descriptor columns plus covalent QC mapping	confirmed	NAC/hinge/C5/tail
WT	7JXH	8c		Cys805	SG	C5	C6	N16	compound-specific C5 moiety atom set	OC2 descriptor columns plus covalent QC mapping	confirmed	NAC/hinge/C5/tail
WT	7JXH	9c		Cys805	SG	C5	C6	N16	compound-specific C5 moiety atom set	OC2 descriptor columns plus covalent QC mapping	confirmed	NAC/hinge/C5/tail
WT	7JXH	10e	8c->10e	Cys805	SG	C27	C26	N37	compound-specific C5 moiety atom set	OC2 descriptor columns plus covalent QC mapping	confirmed	NAC/hinge/C5/tail
WT	7JXH	11f	9c->11f	Cys805	SG	C27	C26	N37	compound-specific C5 moiety atom set	OC2 descriptor columns plus covalent QC mapping	confirmed	NAC/hinge/C5/tail
YVMA	8U8X	3		Cys809	SG	C5	C6	N17	compound-specific C5 moiety atom set	OC2 descriptor columns plus covalent QC mapping	confirmed	NAC/hinge/C5/tail
YVMA	8U8X	4a		Cys809	SG	C5	C6	N22	compound-specific C5 moiety atom set	OC2 descriptor columns plus covalent QC mapping	confirmed	NAC/hinge/C5/tail

YVMA	8U8X	5a	4a->5a	Cys809	SG	C31	C30	N41	compound-specific C5 moiety atom set	OC2 descriptor columns plus covalent QC mapping	confirmed	NAC/hinge/C5/tail
YVMA	8U8X	8c		Cys809	SG	C5	C6	N16	compound-specific C5 moiety atom set	OC2 descriptor columns plus covalent QC mapping	confirmed	NAC/hinge/C5/tail
YVMA	8U8X	9c		Cys809	SG	C5	C6	N16	compound-specific C5 moiety atom set	OC2 descriptor columns plus covalent QC mapping	confirmed	NAC/hinge/C5/tail
YVMA	8U8X	10e	8c->10e	Cys809	SG	C27	C26	N37	compound-specific C5 moiety atom set	OC2 descriptor columns plus covalent QC mapping	confirmed	NAC/hinge/C5/tail
YVMA	8U8X	11f	9c->11f	Cys809	SG	C27	C26	N37	compound-specific C5 moiety atom set	OC2 descriptor columns plus covalent QC mapping	confirmed	NAC/hinge/C5/tail

Table S14. Final covalent-adduct topology validation across the 14 HER2–inhibitor systems.

This table verifies the constructed covalent-adduct state and parameter assignment fields recorded in the master dataset; it is not a validation of reaction kinetics or reaction energetics.

Protein	Compound	Cys residue	Covalent bond	Bond exists	Bond order	Bond length reference (Å)	Electrophilic double-bond state correct	Adduct connectivity verified	Atom types assigned	Partial charges assigned	Bond params assigned	Angle params assigned	Torsion params assigned	Overall QC
WT	3	Cys805	Sγ-Cβ	YES	single	1.8909	YES	YES	YES	YES	YES	YES	YES	PASS
WT	4a	Cys805	Sγ-Cβ	YES	single	1.8997	YES	YES	YES	YES	YES	YES	YES	PASS
WT	5a	Cys805	Sγ-Cβ	YES	single	1.9078	YES	YES	YES	YES	YES	YES	YES	PASS
WT	8c	Cys805	Sγ-Cβ	YES	single	1.8863	YES	YES	YES	YES	YES	YES	YES	PASS
WT	9c	Cys805	Sγ-Cβ	YES	single	1.8807	YES	YES	YES	YES	YES	YES	YES	PASS
WT	10e	Cys805	Sγ-Cβ	YES	single	1.8925	YES	YES	YES	YES	YES	YES	YES	PASS
WT	11f	Cys805	Sγ-Cβ	YES	single	1.8841	YES	YES	YES	YES	YES	YES	YES	PASS
YVMA	3	Cys809	Sγ-Cβ	YES	single	1.8856	YES	YES	YES	YES	YES	YES	YES	PASS
YVMA	4a	Cys809	Sγ-Cβ	YES	single	1.8978	YES	YES	YES	YES	YES	YES	YES	PASS
YVMA	5a	Cys809	Sγ-Cβ	YES	single	1.9036	YES	YES	YES	YES	YES	YES	YES	PASS
YVMA	8c	Cys809	Sγ-Cβ	YES	single	1.884	YES	YES	YES	YES	YES	YES	YES	PASS
YVMA	9c	Cys809	Sγ-Cβ	YES	single	1.8845	YES	YES	YES	YES	YES	YES	YES	PASS
YVMA	10e	Cys809	Sγ-Cβ	YES	single	1.8893	YES	YES	YES	YES	YES	YES	YES	PASS
YVMA	11f	Cys809	Sγ-Cβ	YES	single	1.8864	YES	YES	YES	YES	YES	YES	YES	PASS

Table S15. Hinge-geometry summary across the covalent OC2-generated structural sets.

Distances were measured between the hinge Met backbone N and the compound-specific hinge acceptor. The fraction reports structures with a distance of 3.5 Å or less.

Protein/PDB	Compound	Hinge acceptor	N	Median distance (Å)	Fraction ≤3.5 Å
7JXH	3	N17	1072	3.050	98.97%
7JXH	4a	N22	1121	3.070	94.92%
7JXH	5a	N41	1096	3.068	95.26%
7JXH	8c	N16	1079	3.048	98.89%
7JXH	9c	N16	1147	3.043	99.39%
7JXH	10e	N37	1814	3.047	98.95%
7JXH	11f	N37	1765	3.037	99.49%
8U8X	3	N17	1109	3.049	98.02%
8U8X	4a	N22	1131	3.076	94.87%
8U8X	5a	N41	1087	3.083	93.47%
8U8X	8c	N16	1094	3.054	98.72%
8U8X	9c	N16	1097	3.044	99.45%
8U8X	10e	N37	1818	3.057	98.73%
8U8X	11f	N37	1708	3.040	98.95%

Table S16. SMILES strings of the seven compounds included in the structural analyses.

SMILES strings were taken directly from the molecular-formula/SMILES CSV supplied with the published HER2 inhibitor study.

Compound	SMILES
3	CN(C)C/C=C/C(=O)NC1C(C)=C2C(=NC=NC2=CC=1)NC3=CC(Cl)=C(OCC4=NC=CC=C4)C=C3
4a	CN(C)C/C=C/C(=O)NC1C(C2=CC=CC=C2)=C3C(=NC=NC3=CC=1)NC4C=C(Cl)C(OCC5N=CC=CC=5)=CC=4
5a	C1C=NC(=CC=1)COC2C(Cl)=CC(=CC=2)NC3C4=C(C5=CC=CC=C5)C(NC(=O)/C=C/[C@@H]6N(C)CCC6)=CC=C4N=CN=3
8c	CN(C)C/C=C/C(=O)NC1C2=C3C(=NC=NC3=CC=1)N(CC2)C4=CC(Cl)=C(OCC5=NC=CC=C5)C=C4
9c	CN(C)C/C=C/C(=O)NC1C2=C3C(=NC=NC3=CC=1)N(C=C2)C4=CC(Cl)=C(OCC5=NC=CC=C5)C=C4
10e	C1C=NC(=CC=1)COC2C(Cl)=CC(=CC=2)N3C4C5=C(CC3)C(NC(=O)/C=C/[C@@H]6N(C)CCC6)=CC=C5N=CN=4
11f	C1C=NC(=CC=1)COC2C(Cl)=CC(=CC=2)N3C4C5=C(C=C3)C(NC(=O)/C=C/[C@@H]6N(C)CCC6)=CC=C5N=CN=4

Table S17. Published experimental potency values for the seven compounds in WT HER2 and HER2 YVMA assays.

WT values are biochemical HER2 kinase IC50 measurements; YVMA values are Ba/F3-HER2YVMA antiproliferative IC50 measurements. Values are reported in nM.

Compound	WT HER2 kinase IC_{50} (nM)	Ba/F3-HER2YVMA IC_{50} (nM)
3	12.87	26.22
4a	2.67	5.66
5a	6.03	14.06
8c	1.55	10.80
9c	2.73	9.69
10e	2.96	1.49
11f	2.13	3.82

Source: Yang L, Li Y, Du Y, et al. J. Med. Chem. 2024, 67, 5662–5682. DOI: 10.1021/acs.jmedchem.3c02302. Because the WT and YVMA values were obtained with different assay modalities, they are retained as parallel phenotype axes rather than treated as directly matched measurements.

Table S18. External experimental-structure coverage of the WT and YVMA apo OC-generated structural sets.

Protein	Template	External ref.	Core Cα	N OC	Template RMSD (Å)	OC mean (Å)	SD (Å)	OC median (Å)	Best OC	Best RMSD (Å)	Improvement (Å)	OC < template, n (%)
WT	7JXH	3RCD	232	918	1.568	2.024	0.300	1.986	7JXH_212_1.pdb	1.402	0.166	26 (2.83%)
WT	7JXH	7PCD	238	918	1.580	1.853	0.202	1.822	7JXH_128_3.pdb	1.437	0.142	53 (5.77%)
YVMA	8U8X	8VB5	209	879	1.675	1.517	0.247	1.473	8U8X_294_1.pdb	0.923	0.751	673 (76.56%)

RMSDs were calculated after alignment on the comparison-specific common/fixed kinase-core Cα set. Improvement is reported as the positive magnitude of the decrease from the starting-template RMSD to the best OC RMSD. These values are used as coverage diagnostics rather than estimates of equilibrium populations or free energies. For 8VB5, the reported comparison uses the QC-defined fixed 209-Cα kinase-core correspondence.

OC2 input protein sequences

The exact protein sequences used as inputs for OC2 generation are reported below for reproducibility. The HER2^{WT} and HER2^{YVMA} inputs contained 322 and 326 residues, respectively, and differed only by the four-residue YVMA insertion.

HER2^{WT} (7JXH-based input; 322 aa):

SGAMPNQAQMRILKETELRKVKVLGSGAFGTVYKGIWIPDGENVKIPVAIKVLRENTSPKANKEILDEAYVMAGVGSPYVSRLLGICLTSTVQLVTQLM
PYGCLLDHVRENRGRLGSQDLLNWCMQIAKGMSYLEDVRLVHRDLAARNVLVKSPNHVKITDFGLARLLDIDETEYHADGGKVPIKWMALESILRRRF
THQSDVWSYGVTVWELMTFGAKPYDGIPAREIPDLLEKGERLPQPPICTIDVYMIMVKCWMIDSECRPRFRELVSEFSRMARDPQRFVVIQNEDLGPASP
LDSTFYRSLLEDDDMGDLVDAEEYL

HER2^{YVMA} (8U8X-based input; 326 aa):

SGAMPNQAQMRILKETELRKVKVLGSGAFGTVYKGIWIPDGENVKIPVAIKVLRENTSPKANKEILDEAYVMAYVMAGVGSPYVSRLLGICLTSTVQLV
TQLMPYGCLLDHVRENRGRLGSQDLLNWCMQIAKGMSYLEDVRLVHRDLAARNVLVKSPNHVKITDFGLARLLDIDETEYHADGGKVPIKWMALESI
LRRRFTHQSDVWSYGVTVWELMTFGAKPYDGIPAREIPDLLEKGERLPQPPICTIDVYMIMVKCWMIDSECRPRFRELVSEFSRMARDPQRFVVIQNEDL
GPASPLDSTFYRSLLEDDDMGDLVDAEEYL

S3. Supplementary Figures

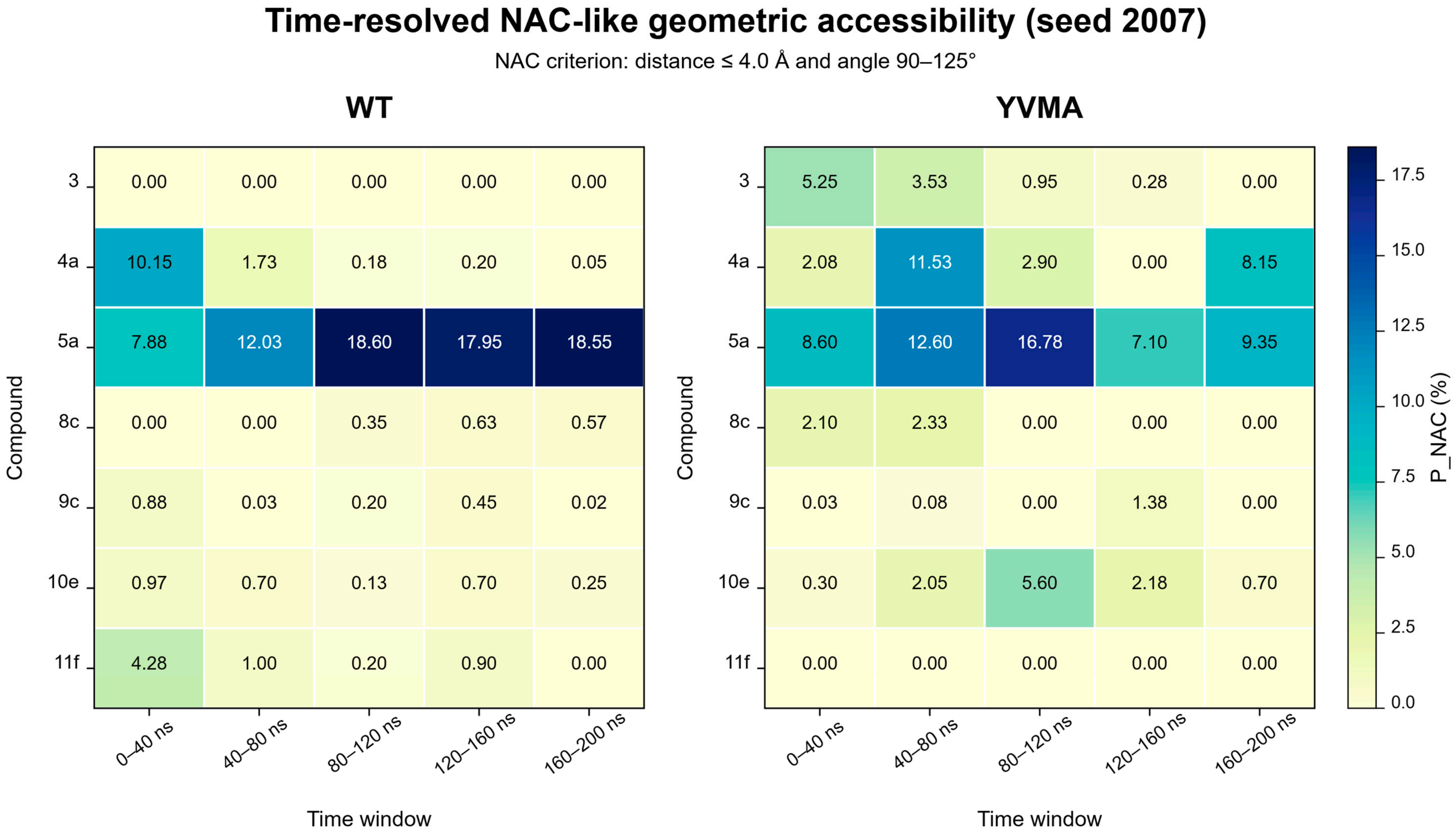

Figure S1. Time-resolved reaction-compatible geometry in pre-reaction MD replica seed 2007. Heatmaps show P_NAC (%) in five consecutive 40 ns windows (0–40, 40–80, 80–120, 120–160, and 160–200 ns) for WT HER2 and HER2 YVMA under the primary reaction-compatible geometry criterion (Sγ–Cβ distance ≤4.0 Å and Sγ–Cβ–Cα angle 90–125°). Windowed values were used to visualize temporal heterogeneity within the trajectory and were not treated as independent replicates.

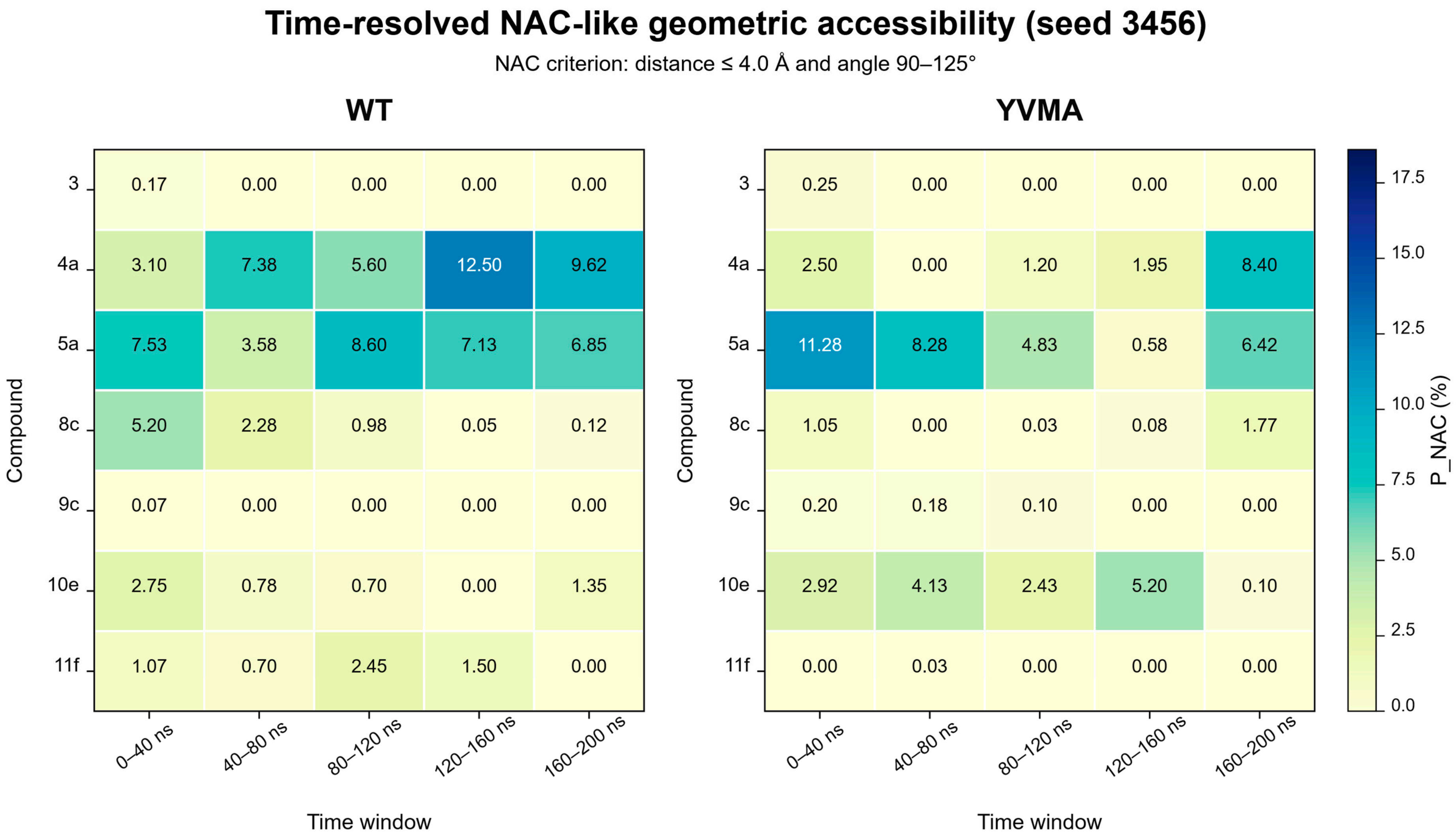

Figure S2. Time-resolved reaction-compatible geometry in pre-reaction MD replica seed 3456. Heatmaps show P_NAC (%) in five consecutive 40 ns windows (0–40, 40–80, 80–120, 120–160, and 160–200 ns) for WT HER2 and HER2 YVMA under the primary reaction-compatible geometry criterion (Sγ–Cβ distance ≤4.0 Å and Sγ–Cβ–Cα angle 90–125°). Windowed values were used to visualize temporal heterogeneity within the trajectory and were not treated as independent replicates.

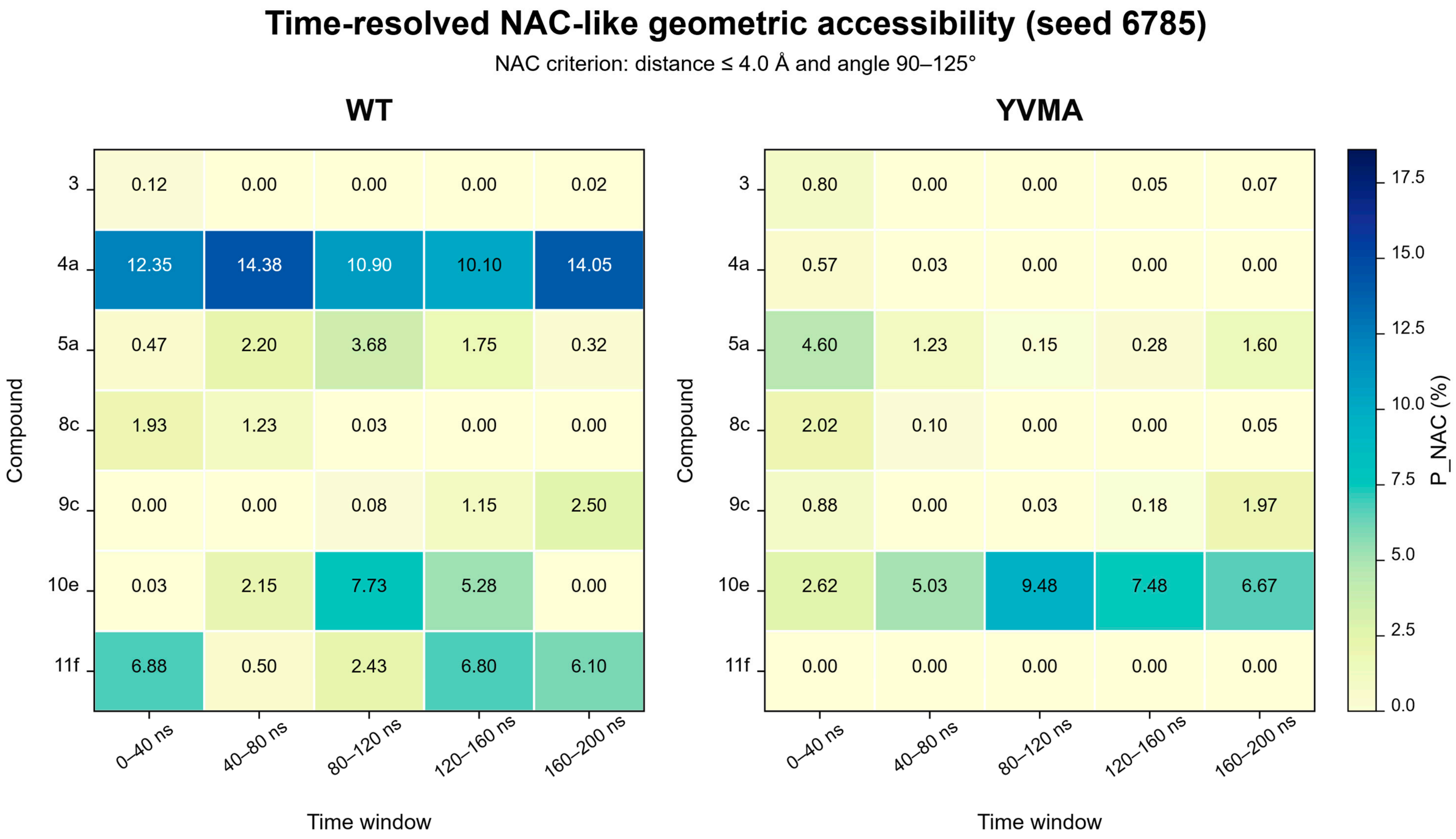

Figure S3. Time-resolved reaction-compatible geometry in pre-reaction MD replica seed 6785. Heatmaps show P_NAC (%) in five consecutive 40 ns windows (0–40, 40–80, 80–120, 120–160, and 160–200 ns) for WT HER2 and HER2 YVMA under the primary reaction-compatible geometry criterion (Sγ–Cβ distance ≤4.0 Å and Sγ–Cβ–Cα angle 90–125°). Windowed values were used to visualize temporal heterogeneity within the trajectory and were not treated as independent replicates.

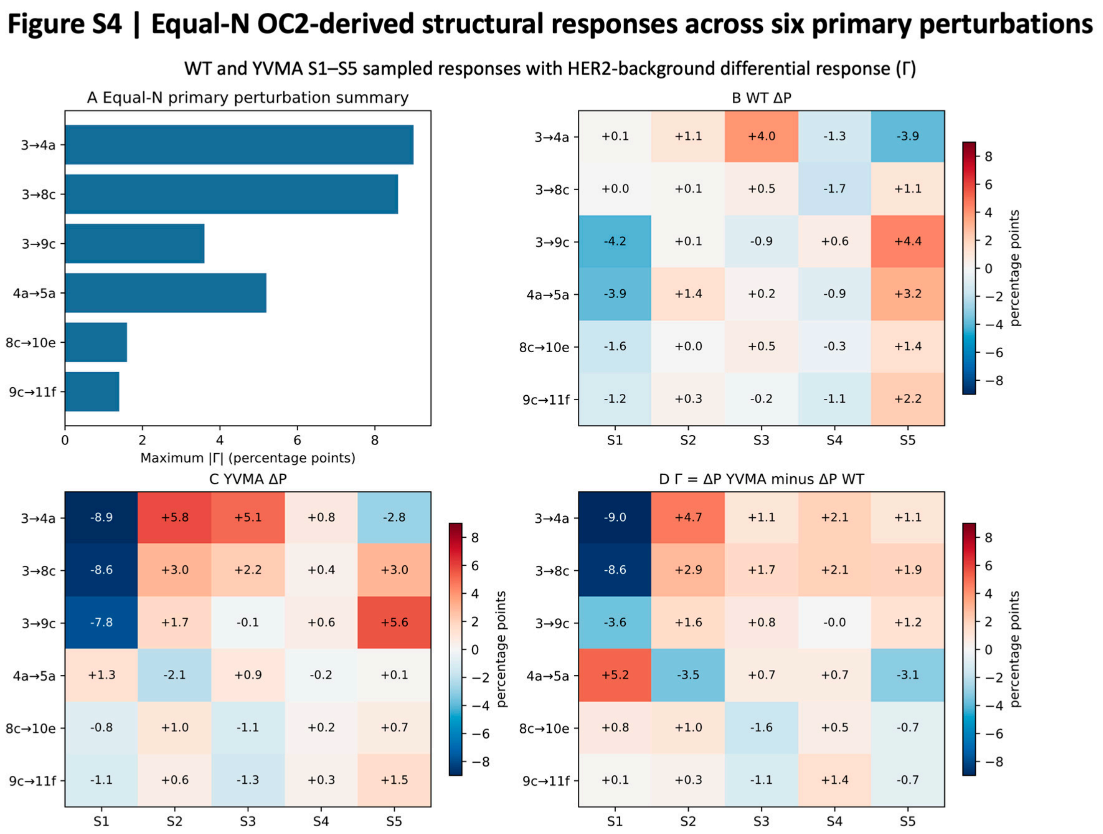

Figure S4. OC2 perturbation-response map derived from covalently linked HER2–inhibitor OC2-generated structural sets. Panel A summarizes perturbation-level maximum Γ values. Panels B and C show WT and YVMA differential responses across the operational k=5 representation. Panel D shows protein-background dependence (Γ = ΔP(YVMA) – ΔP(WT)).

Figure S5 | Compound-level covalent HOLO OC2 sampled structural representation

Covalent descriptor-space distributions across all seven compounds in WT and YVMA

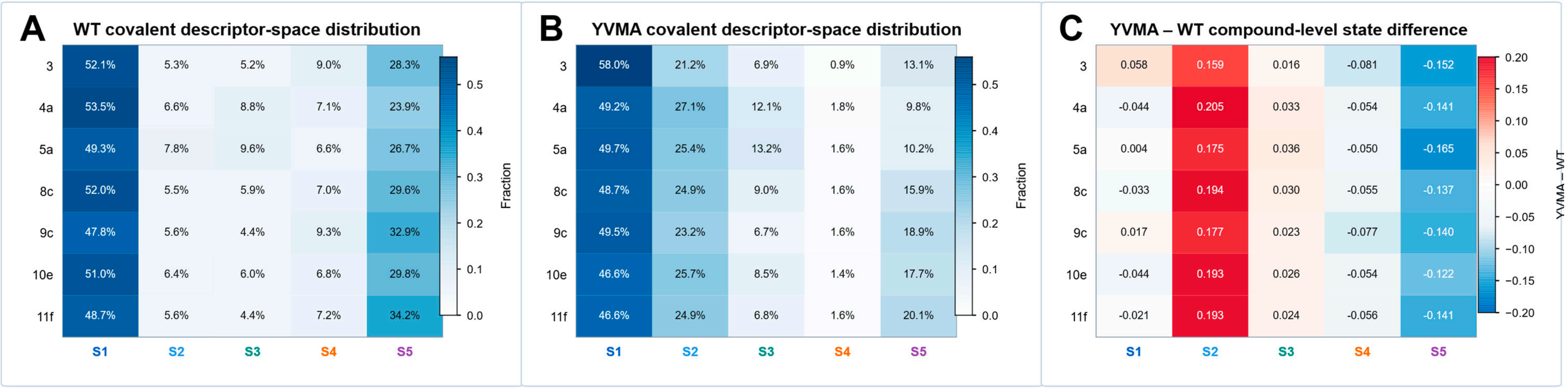

Figure S5 reports compound-level descriptor-space distributions; it is distinct from the perturbation-level ΔP/T analysis in OC2.

Figure S5. Compound-level covalent OC2 sampled representations in WT and YVMA backgrounds. The figure compares sampled representation fractions across compounds and reports YVMA–WT differences within the same operational coordinate.

Figure S6 | Robustness of OC2-derived HER2-background differential responses

Comparison of full-data and Equal-N Γ under the primary and alternative apo WT references

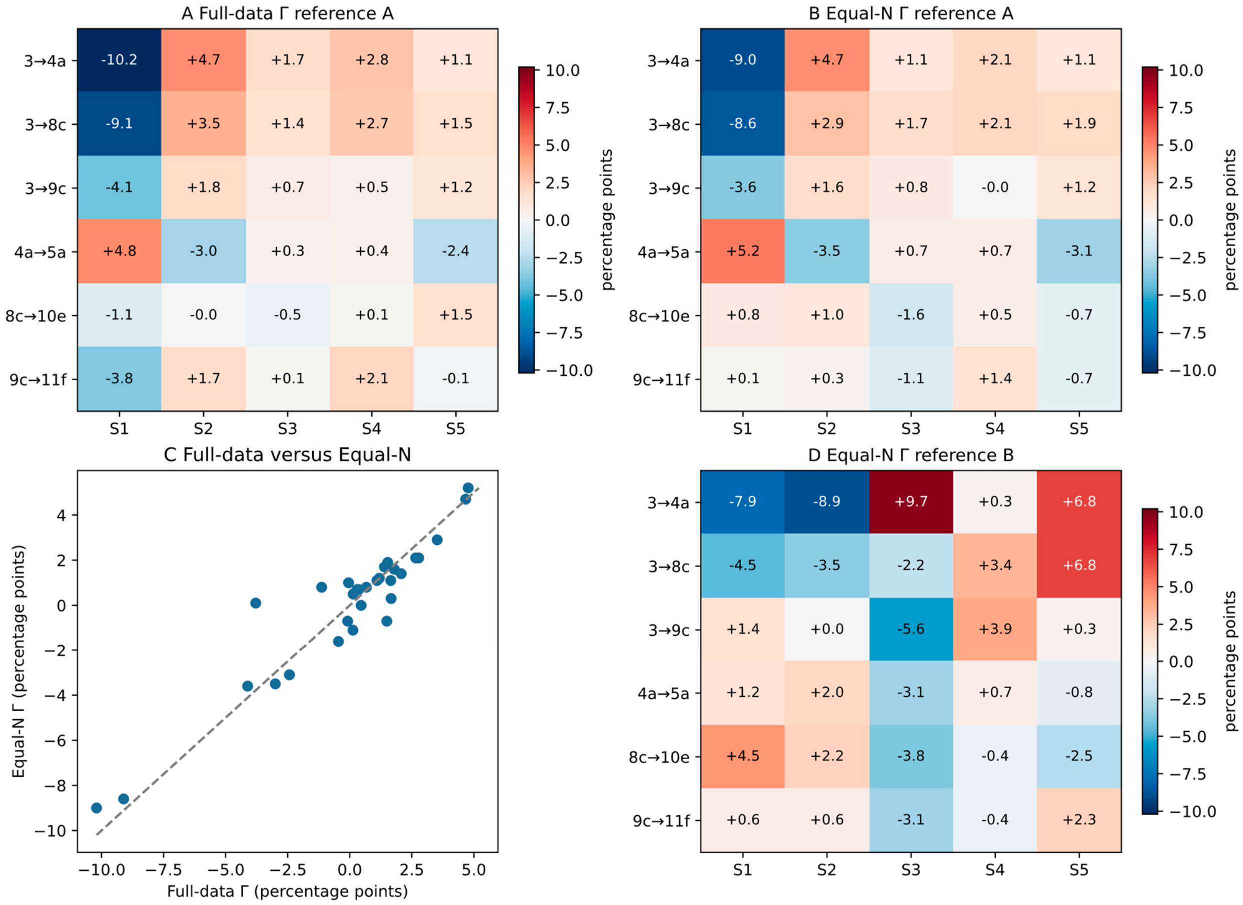

Figure S6. Sensitivity analysis of covalent OC2-derived responses. Heatmaps compare the full-data Γ matrix with the Equal-N control, and the scatter plot summarizes concordance between the two analyses.

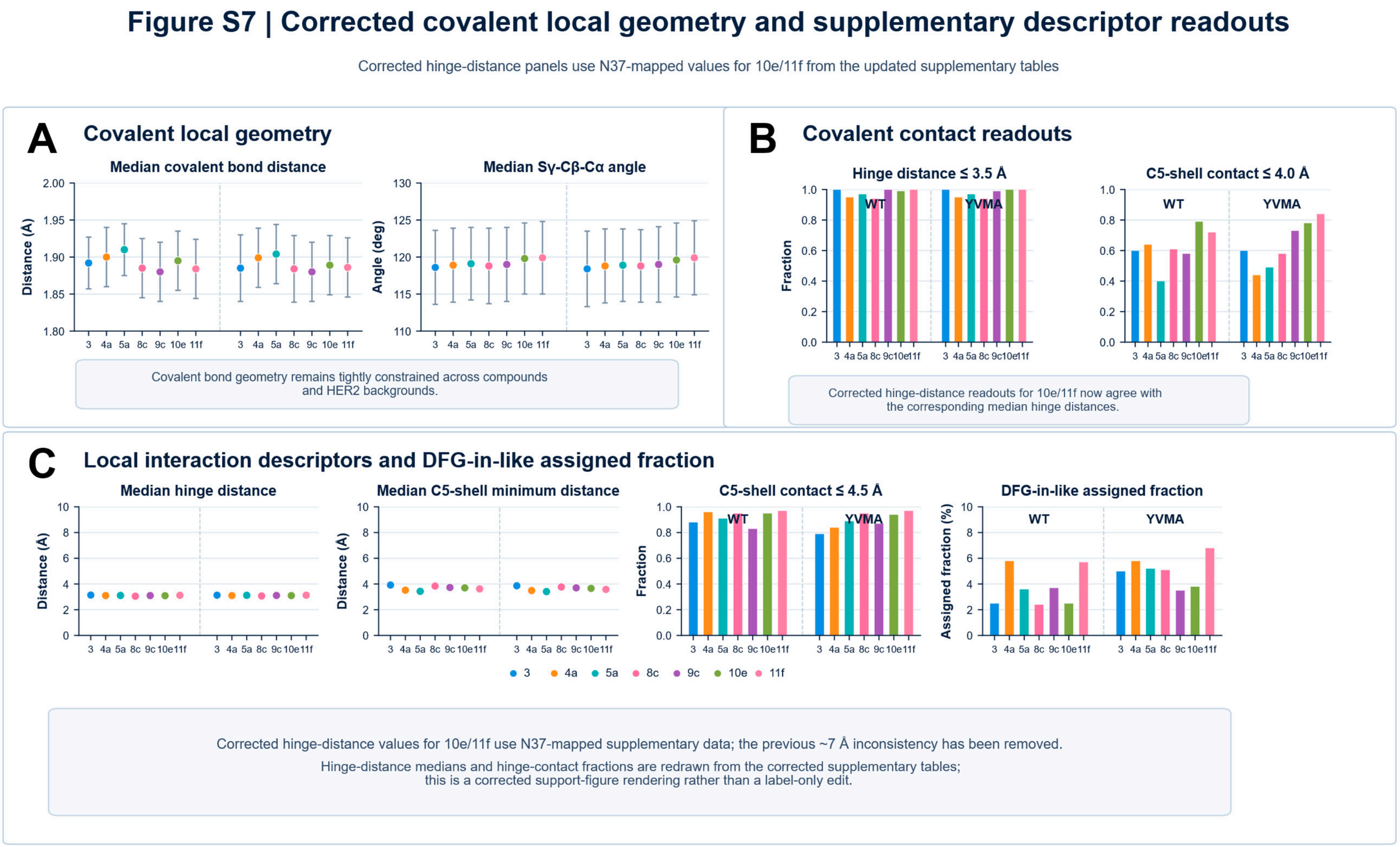

Figure S7. Supplementary local structural descriptors derived from covalent OC2-generated structural sets, including covalent local geometry, covalent contact readouts, local interaction descriptors, and DFG-region geometric descriptors.

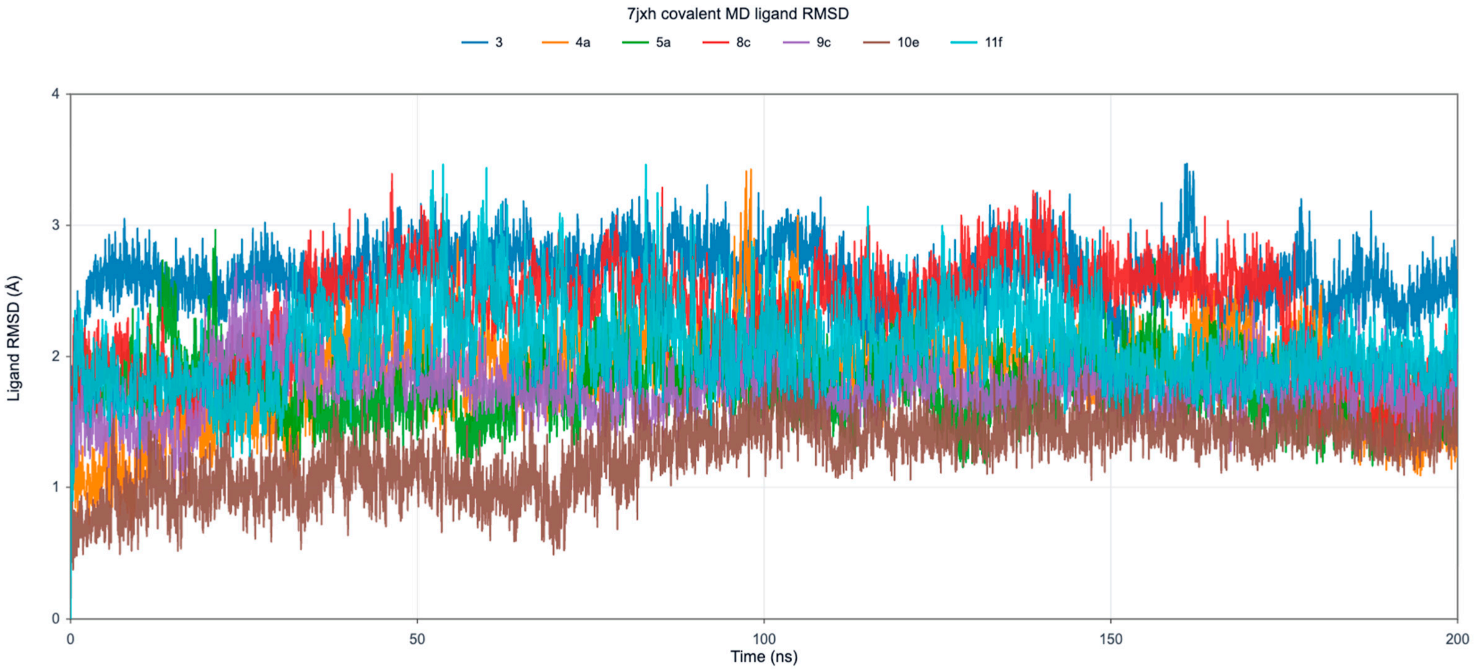

Figure S8. HER2WT (7JXH) covalent-adduct MD ligand RMSD time series.

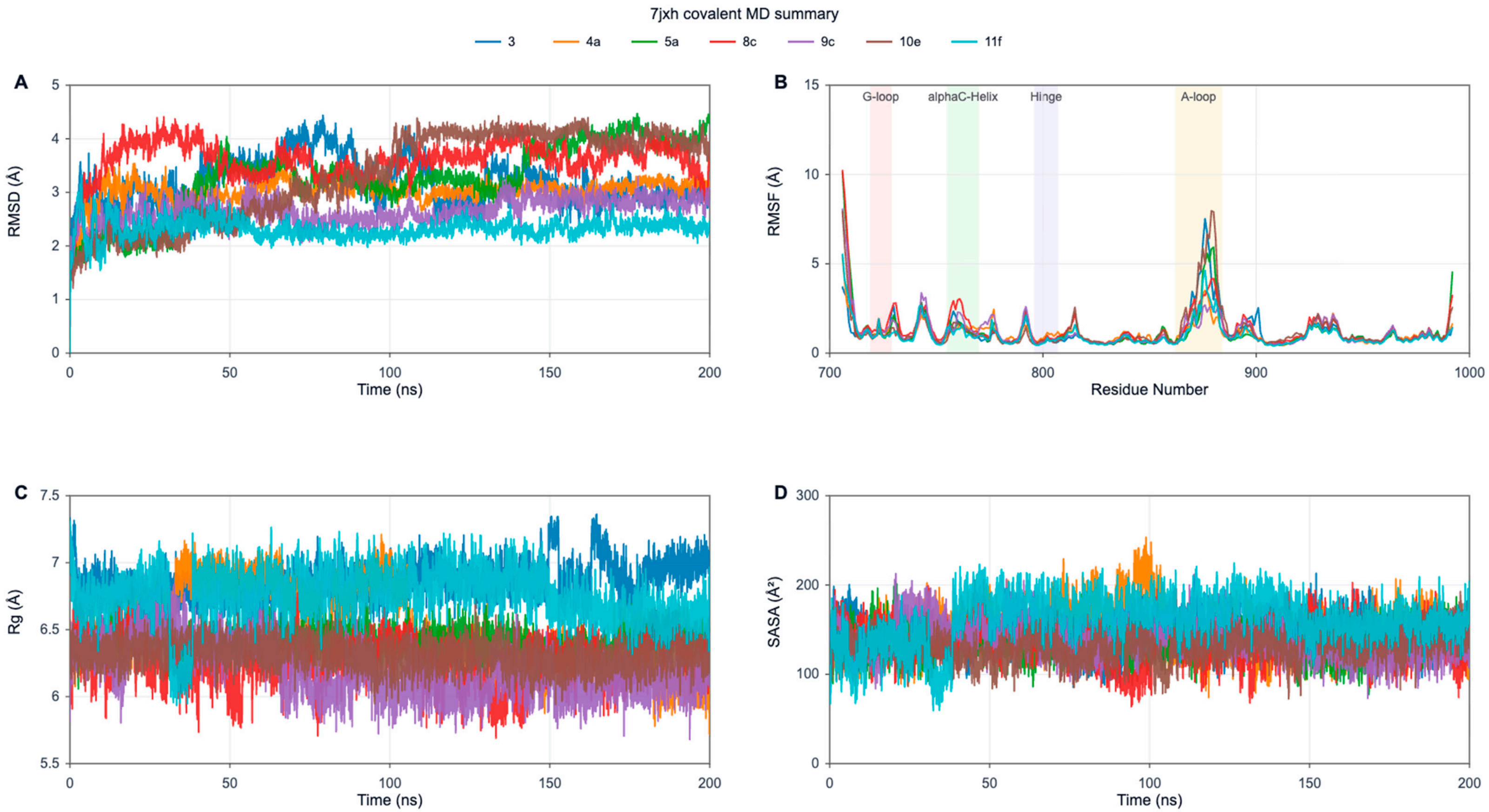

Figure S9. HER2WT (7JXH) covalent-adduct MD summary, including RMSD, RMSF, radius of gyration, and SASA.

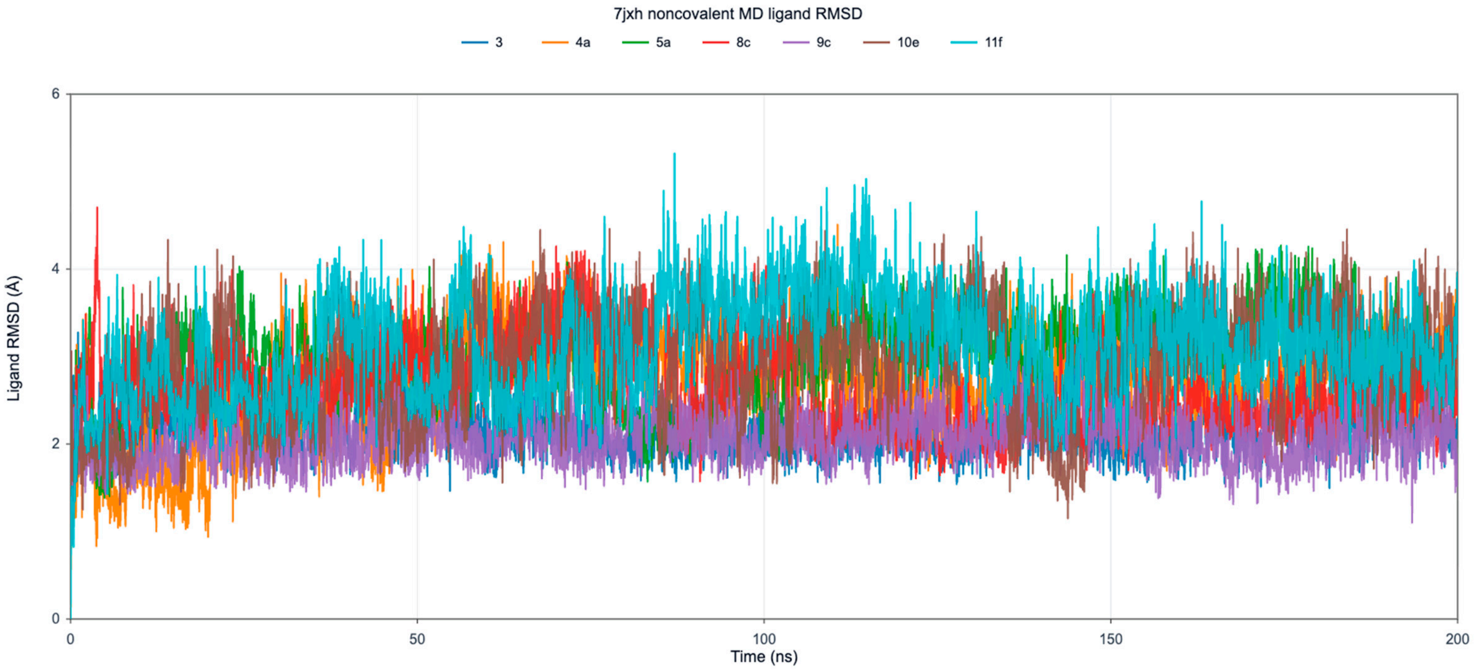

Figure S10. HER2WT (7JXH) noncovalent MD ligand RMSD time series.

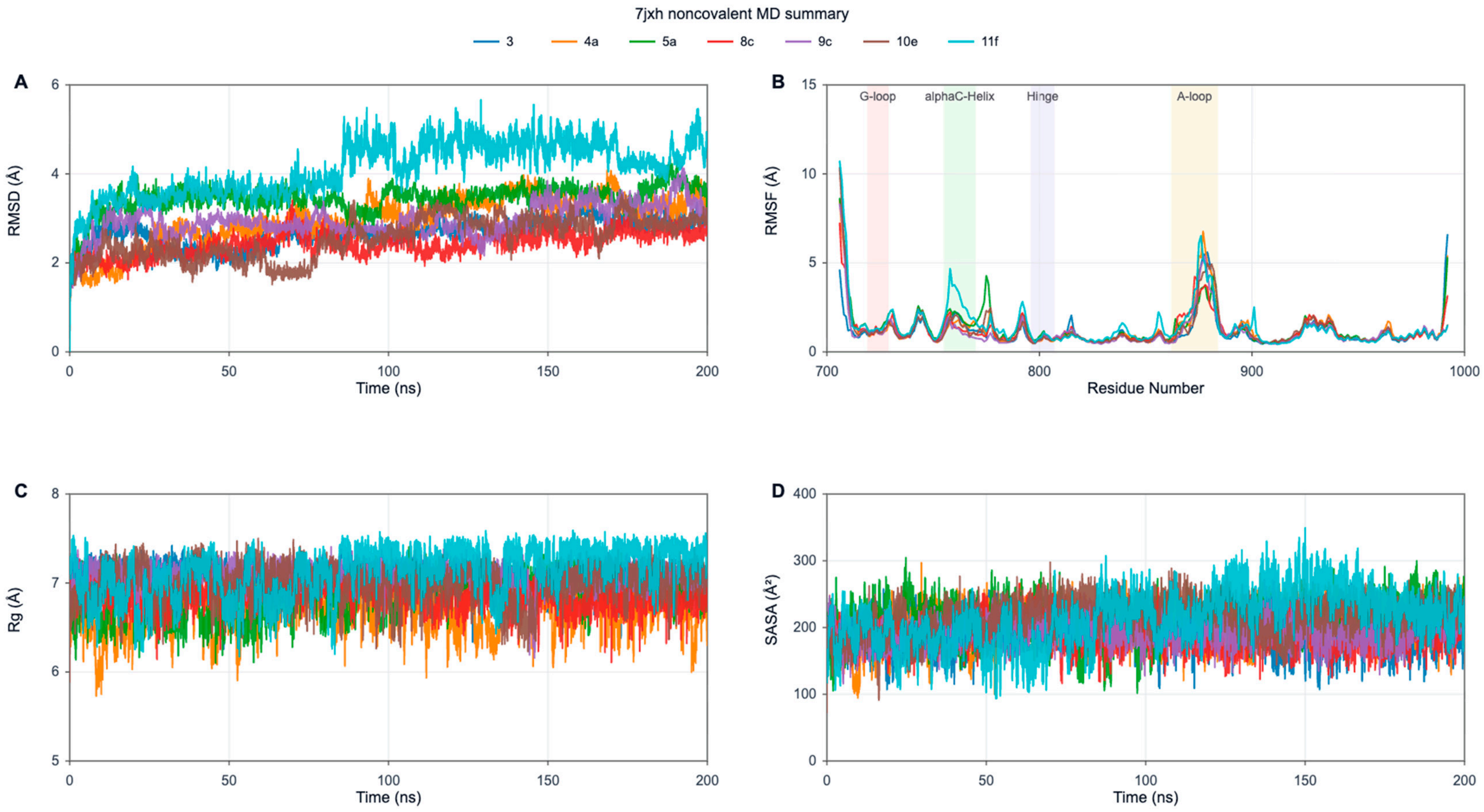

Figure S11. HER2WT (7JXH) noncovalent MD summary, including RMSD, RMSF, radius of gyration, and SASA.

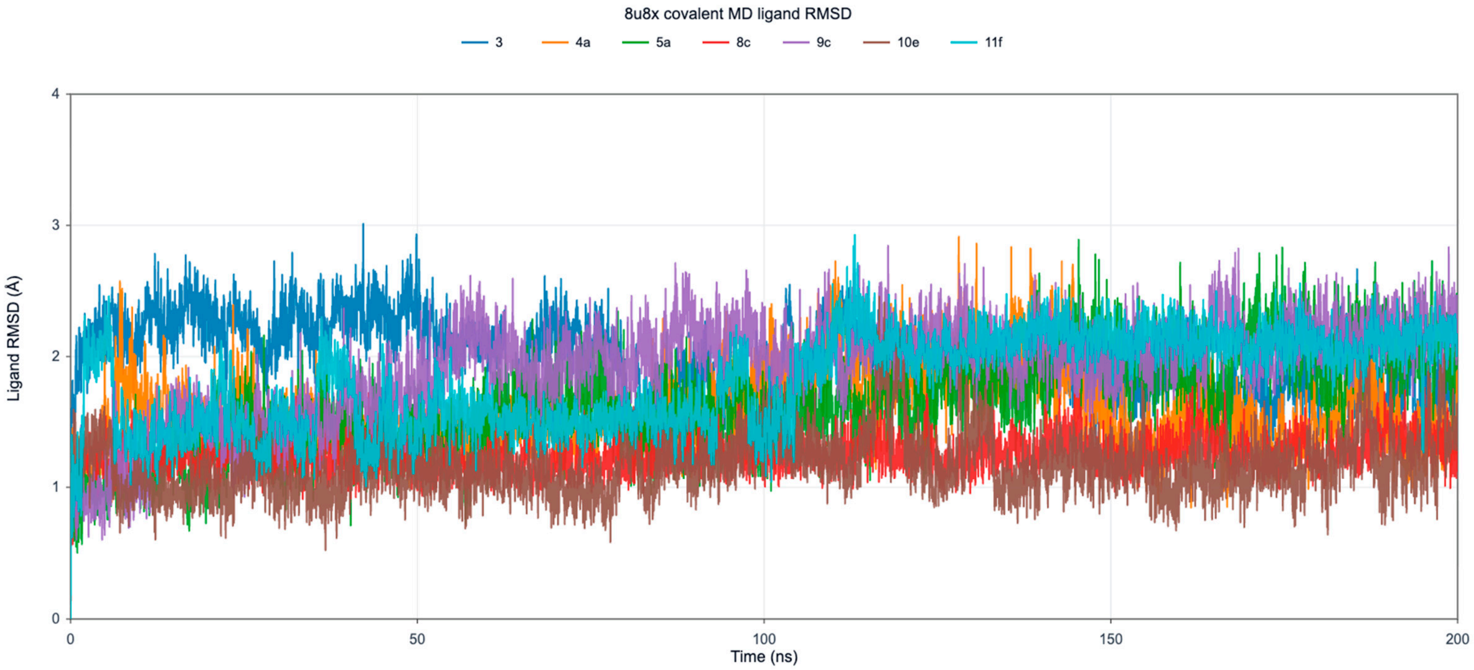

Figure S12. HER2YVMA (8U8X) covalent-adduct MD ligand RMSD time series.

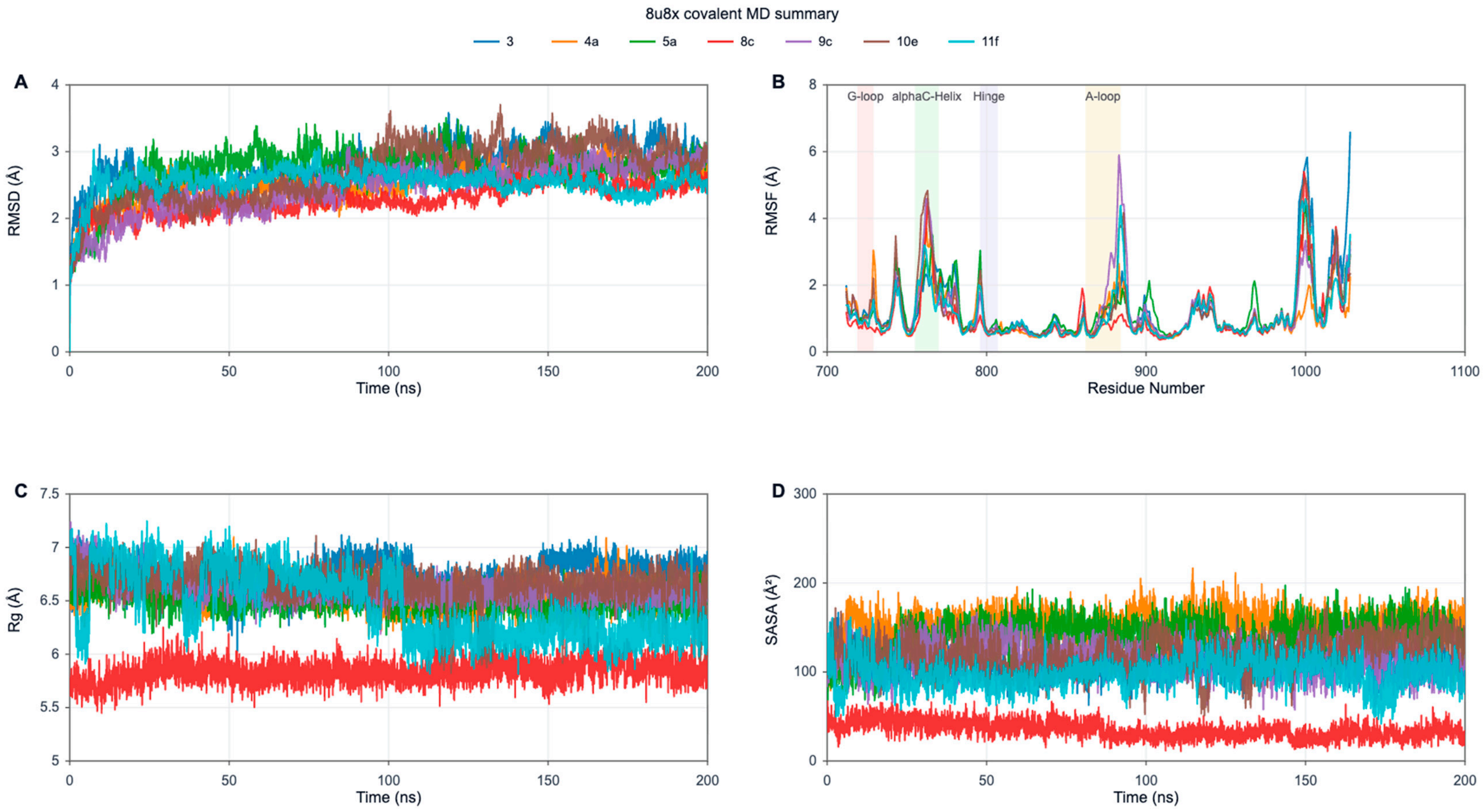

Figure S13. HER2YVMA (8U8X) covalent-adduct MD summary, including RMSD, RMSF, radius of gyration, and SASA.

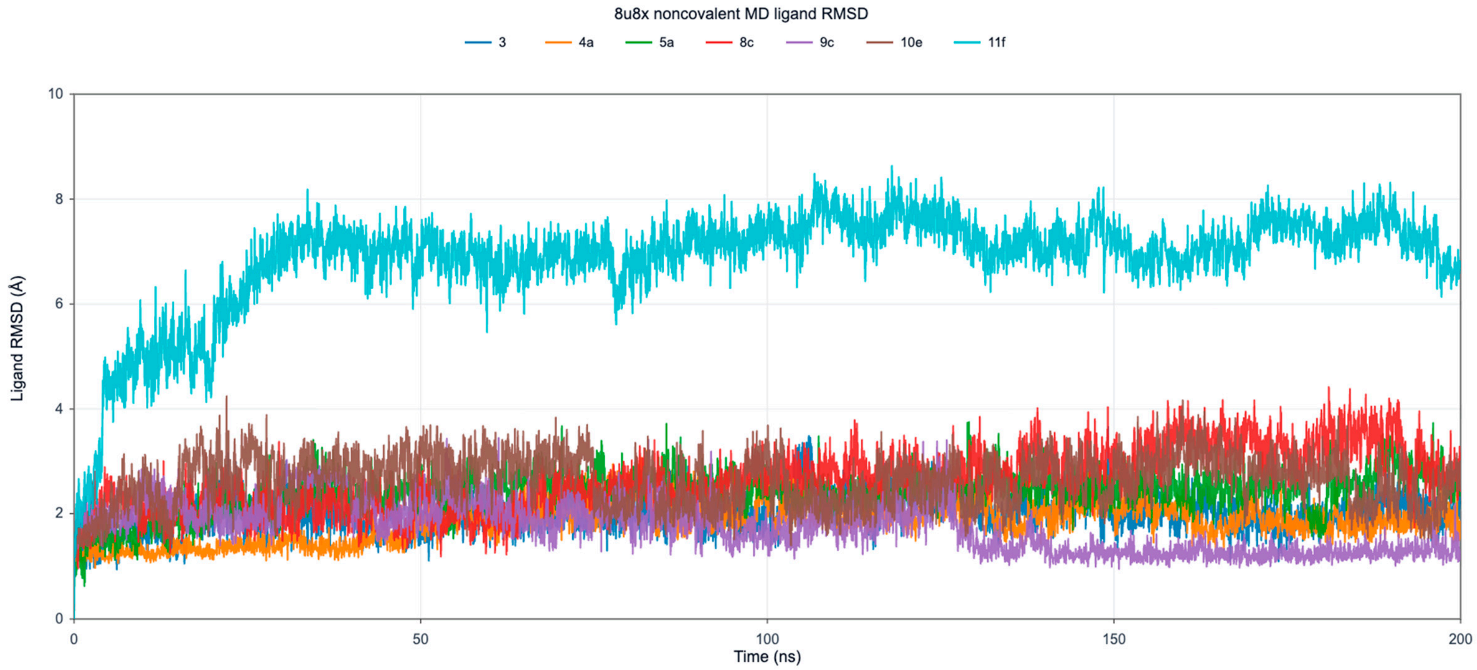

Figure S14. HER2YVMA (8U8X) noncovalent MD ligand RMSD time series.

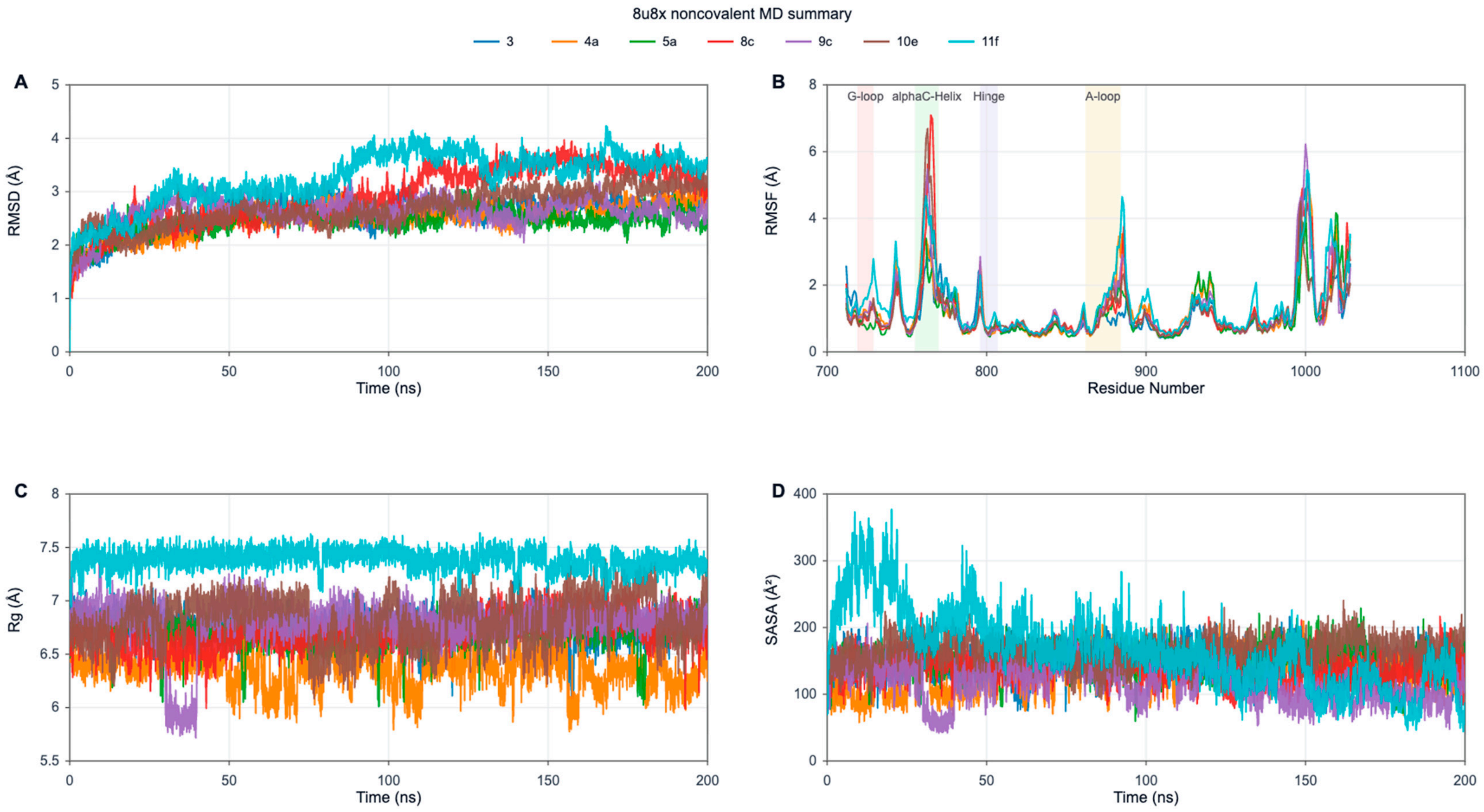

Figure S15. HER2YVMA (8U8X) noncovalent MD summary, including RMSD, RMSF, radius of gyration, and SASA.

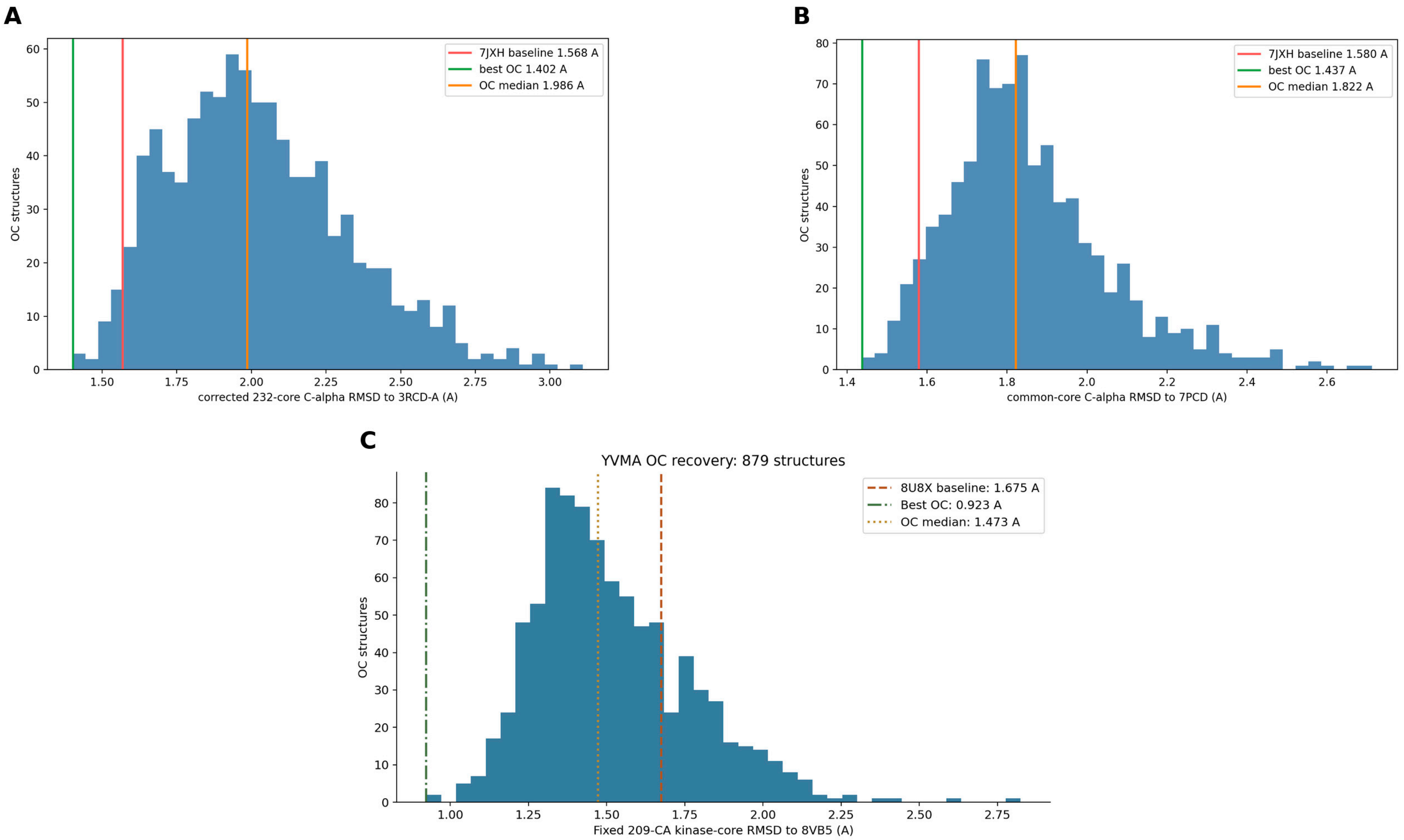

Figure S16. External experimental-structure coverage of the apo OC-generated structural sets. (A) WT versus 3RCD using the corrected 232-Cα core. (B) WT versus 7PCD using the 238-Cα common core. (C) YVMA versus 8VB5 using the fixed 209-Cα kinase core. Vertical lines mark the starting-template RMSD, best OC structure, and OC median. Distributions are interpreted as conformational-coverage diagnostics rather than equilibrium populations.

Interpretation framework: reaction-compatible geometry is used to describe pre-reactive geometric accessibility; OC2 analyses use continuous structural descriptors as primary readouts and k=5 as an operational sampled representation; covalent-adduct MD analyses characterize final-adduct accommodation.

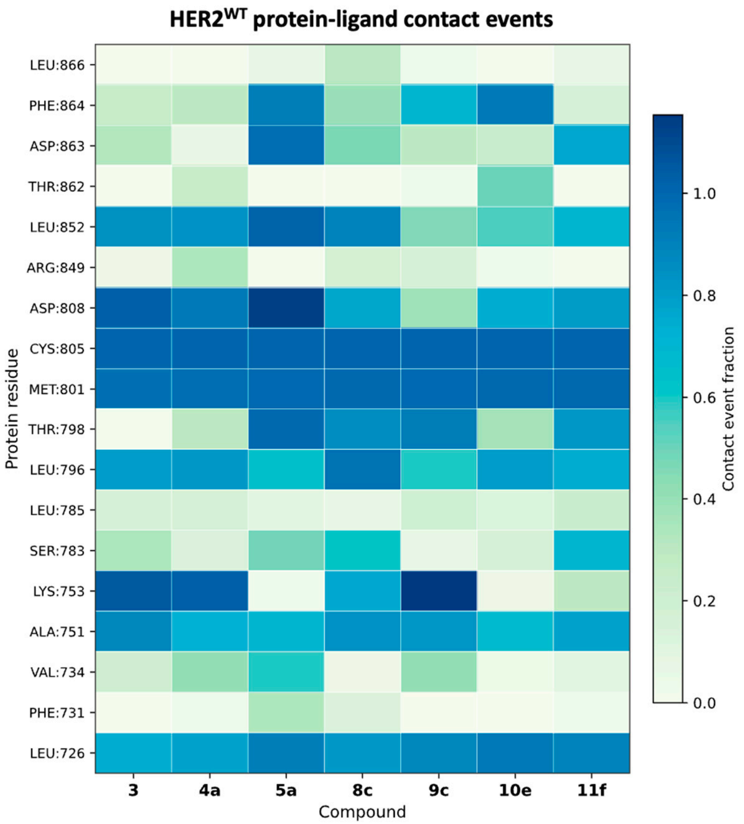

Figure S17. HER2^{WT} (7JXH) covalent-adduct MD protein-ligand contact-event heatmap for replica seed 2007. Rows denote protein residues and columns denote compounds 3, 4a, 5a, 8c, 9c, 10e, and 11f; color intensity represents the trajectory-derived contact-event metric shown in the heatmap.

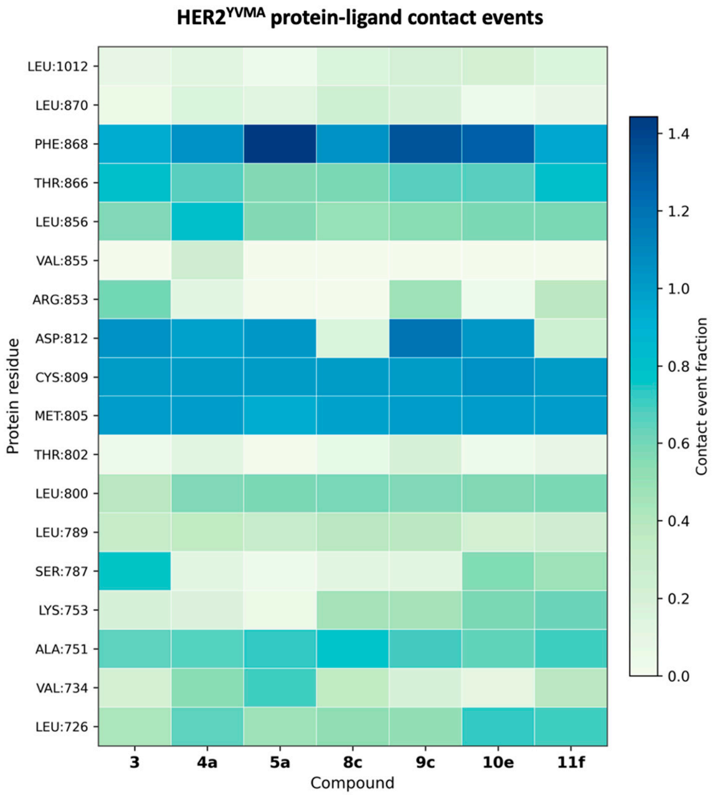

Figure S18. HER2^{YVMA} (8U8X) covalent-adduct MD protein-ligand contact-event heatmap for replica seed 2007. Rows denote protein residues and columns denote compounds 3, 4a, 5a, 8c, 9c, 10e, and 11f; color intensity represents the trajectory-derived contact-event metric shown in the heatmap.

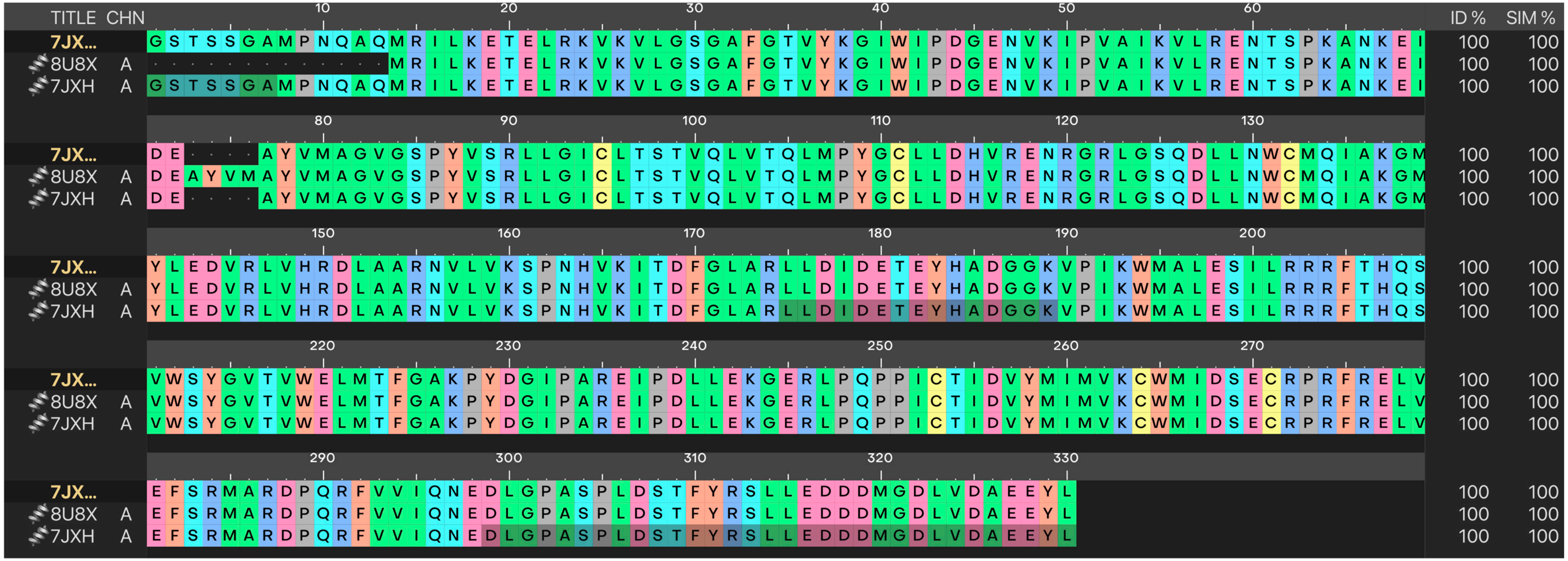

Figure S19. Full sequence alignment of the HER2^{WT} (7JXH) and HER2^{YVMA} (8U8X) constructs used in the Maestro-based workflows, showing the complete modeled sequences and the four-residue YVMA insertion in the HER2YVMA construct.
